# A systems biology pipeline identifies new immune and disease related molecular signatures and networks in human cells during microgravity exposure

**DOI:** 10.1038/srep25975

**Published:** 2016-05-17

**Authors:** Sayak Mukhopadhyay, Rohini Saha, Anbarasi Palanisamy, Madhurima Ghosh, Anupriya Biswas, Saheli Roy, Arijit Pal, Kathakali Sarkar, Sangram Bagh

**Affiliations:** 1 Biophysics and Structural Genomics Division, Saha Institute of Nuclear Physics, Kolkata, 700064, India; 2 Department of Biological Sciences, Presidency University, Kolkata, 700073, India

## Abstract

Microgravity is a prominent health hazard for astronauts, yet we understand little about its effect at the molecular systems level. In this study, we have integrated a set of systems-biology tools and databases and have analysed more than 8000 molecular pathways on published global gene expression datasets of human cells in microgravity. Hundreds of new pathways have been identified with statistical confidence for each dataset and despite the difference in cell types and experiments, around 100 of the new pathways are appeared common across the datasets. They are related to reduced inflammation, autoimmunity, diabetes and asthma. We have identified downregulation of NfκB pathway via Notch1 signalling as new pathway for reduced immunity in microgravity. Induction of few cancer types including liver cancer and leukaemia and increased drug response to cancer in microgravity are also found. Increase in olfactory signal transduction is also identified. Genes, based on their expression pattern, are clustered and mathematically stable clusters are identified. The network mapping of genes within a cluster indicates the plausible functional connections in microgravity. This pipeline gives a new systems level picture of human cells under microgravity, generates testable hypothesis and may help estimating risk and developing medicine for space missions.

The future plan of manned mission to Mars and asteroids^1^ requires astronauts to spend years in space. Microgravity is one of the most prominent health hazards for astronauts^2^,^3^. During today’s space missions, a short to moderate microgravity exposure (days to months) induces several physiological changes in human body including bone and muscle loss, puffiness in the face, change in cardiovascular physiology, catecholamine cardiomyopathy, insufficient blood flow in the brain, genitourinary issues and disturbance in neurovestibular system^2^,^3^,^4^,^5^,^6^,^7^. Further, microgravity induces deregulation of human immune systems^8^,^9^. Multiple gene expression studies showed microgravity-induced signature of early inhibition in T cell activation^10^, impaired endothelial cell function^11^, cellular senescence^12^, alteration of genes related to cell cycle^13^,^14^, cell adhesion^11^, oxidative phosphorylation^14^ and apoptosis^14^. It has been showed that the reduced immunity may result from inhibition of NF- κB/Rel pathway, downregulation of early T cell activation genes, IFN- ϒ and EL-2Rα genes^15^ and impairment of Jun-N-terminal kinase activity^9^. The compromised immune system increases the risk of infection by pathogen like salmonella, virulence of which is increased in microgravity^16^. Salmonella infection among astronauts is a well-known health hazard documented starting from Apollo and Skylab missions^16^,^17^.

Further, microgravity alters level of micro RNAs (miRNAs), many of which are related with inflammation^18^ and multiple cancer types^13^,^18^,^19^. However, the studies showed controversial inference based on the expression of different microRNAs. For example, expression of hsa-miR-423-5p and hsa-miR-222 in microgravity suggest the induction of breast cancer, whereas expression of hsa-miR-141 suggests the decrease in the same^19^. Similar controversial miRNA expression pattern was observed for leukaemia and lung cancer^18^,^19^. Further, as a single miRNA is related with several cancer types and opposite results in miRNA alternation are observed among studies^13^,^18^,^19^, there is uncertainty to identify specific cancer signatures, if any, associated with microgravity. No cancer related signatures and inflammation signature were identified in normal human cells through gene expression data alone. Thus the connection of cancer induction with microgravity is undefined and no assessment reports included microgravity-associated cancer as a risk factor. However, the ambitious plan for sending humans to Mars and asteroids requires a thorough understanding about the effect of microgravity at the cellular level to estimate the risk for all potential diseases and health conditions and develop protocols against any adverse effect of space on the astronauts. In spite of its prevalence, a detailed molecular systems level picture on how various molecular pathways in human cells get affected by microgravity is largely unknown.

In the previous microgravity studies, the transcriptomics data of human cells were analysed by differential gene expression analysis, followed by passive pathway mapping^14^,^15^,^18^,^20^. Differential gene expression analysis relies on arbitrary cut-off value (>1.5–2 folds) in fold change of individual genes. It may overlook the pathway level picture due to absence of genes with lower expression values. For example, this gene centric method cannot identify the downregulation of oxidative phosphorylation pathway in diabetes, where the mean decrease in member genes’ expression is about 1.2 folds^21^,^22^. This is specifically critical for the situation like microgravity, which results an overall low fold change in the global gene expression compare to other perturbation like cancers^23^,^24^. Further, previous studies relied on KEGG, GO databases and a few manually curated ontologies for pathway analysis, missing a huge number of disease and immunity related pathways. The assignments of the pathways were also arbitrary. Pathways were assigned even when the fraction of mapped genes were as low as 2% of the whole pathway^14^,^20^.

In this work, by integrating a different set of systems biology tools and databases, we have analysed the effect of microgravity on more than 8000 molecular pathways on normal human cells from published global gene expression data. We have identified new pathways, mechanism and plausible regulatory and functional connections across the gene networks in microgravity, which cannot be identified by conventional analysis.

## The gene expression data

The global gene expression datasets from 5 important works were mined from ArrayExpress. Three out of those five experiments were performed in space-flight conditions. Dataset with accession number E-GEOD-38836^15^ represents International Space Station (ISS) study of human T-cells, E-GEOD-43582^14^ represents Progress 40 P space flight mission (ESA-SPHINX) with human umbilical vein endothelial cells (HUVEC) and E-GEOD-54213 [unpublished] represents space shuttle STS-135 study of human endothelial cells. Datasets E-GEOD-4209^20^ and E-GEOD-57418^18^ represent ground based simulated microgravity studies with activated human T-cells and human peripheral blood lymphocyte (PBMC) respectively. To our knowledge, no other normal human cell gene expression study under microgravity condition is available in the public domain.

### Database for human molecular pathways

Those 8000 molecular pathways, which covers almost all known aspects of human cell including positional (chromosomal) gene set, chemical and genetic perturbation, canonical pathways (cellular, metabolic and disease pathways from KEGG, Biocarta and Reactome), cancer gene neighbourhoods, cancer modules, oncogenic signatures, immunogenic signature and hallmark gene sets, were extracted from Molecular Signature Database (MSigDB), which brought together pathways from all standard databases on human. However, we have excluded GO classification, as it appears too broad and less specific.

### Identification of pathways and leading genes

**Figure 1:**
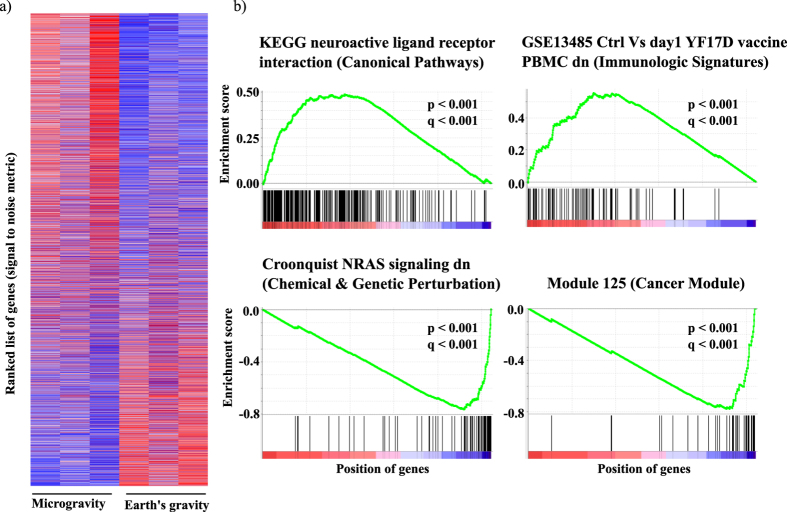
GSEA analysis of human cell during microgravity exposure. (**a**) Genome wide expression profile of E-GEOD-4209 for three biological replicates in microgravity and normal gravity. The heatmap represents ranked genes, which is created by signal-to-noise matric (fold change in expression between microgravity and normal gravity divided by the standard deviation in gene expression among the replicates). (**b**) Enrichment score of four representative, statistically significant pathways from four different gene set modules. The top and bottom figures represent upregulated and downregulated pathways respectively. The heatmap of the ranked gene list is shown at the bottom of each pathway. The black lines within the heatmap represent the position of the pathway genes in that ranked list.

We have applied Gene Set Enrichment Analysis (GSEA)^21^, which determines the likeliness of any specific molecular pathways (gene set) to be involved with microgravity directly from the global gene expression data, in a statistically significant way. The p value and FDR q value of entire gene set are calculated in GSEA. Thus, unlike passive pathway mapping in differential gene expression analysis, each altered pathway in GSEA is chosen or discarded based on statistical parameters. In GSEA, from the global gene expression data, a ranked list of genes is prepared according to the difference in gene expression values between microgravity and normal gravity divided by the standard deviation between biological replicates (Fig. 1a). A running sum statistics estimates the likeliness of genes from a specific molecular pathway to be appeared in top or bottom of the list in a statistically significant way (Fig. 1b). Details of the method can be found in methods section. Next we have identified leading edge genes (Supplementary Table S4) before enrichment score (ES) peak values (Fig. 1b). Leading edge genes contributed the most for a pathway to be altered in microgravity.

### Extracting gene expression patterns and networks in microgravity

We have applied an unsupervised non-negative matrix factorization coupled with a consensus-clustering algorithm (NMFC)^25^,^26^ to extract the genes (leading edge genes) with similar expression patterns in microgravity. NMFC was found superior to extract the functional meaningful subset of genes from genome scale transcriptomics data compare to other clustering algorithm including principal component analysis, K means, self organizing maps, singular value decomposition and hierarchical clustering^26^,^27^,^28^. The genes with similar expression patterns within a mathematically stable cluster suggest plausible regulatory or functional connections in microgravity. Those genes within a single cluster were further mapped on STRING network databases^29^ to evaluate the functional networks. Further details about the NMFC and network mapping can be found in results and method sections.

## Results

### Molecular pathway analysis using gene set enrichment analysis (GSEA)

**Figure 2:**
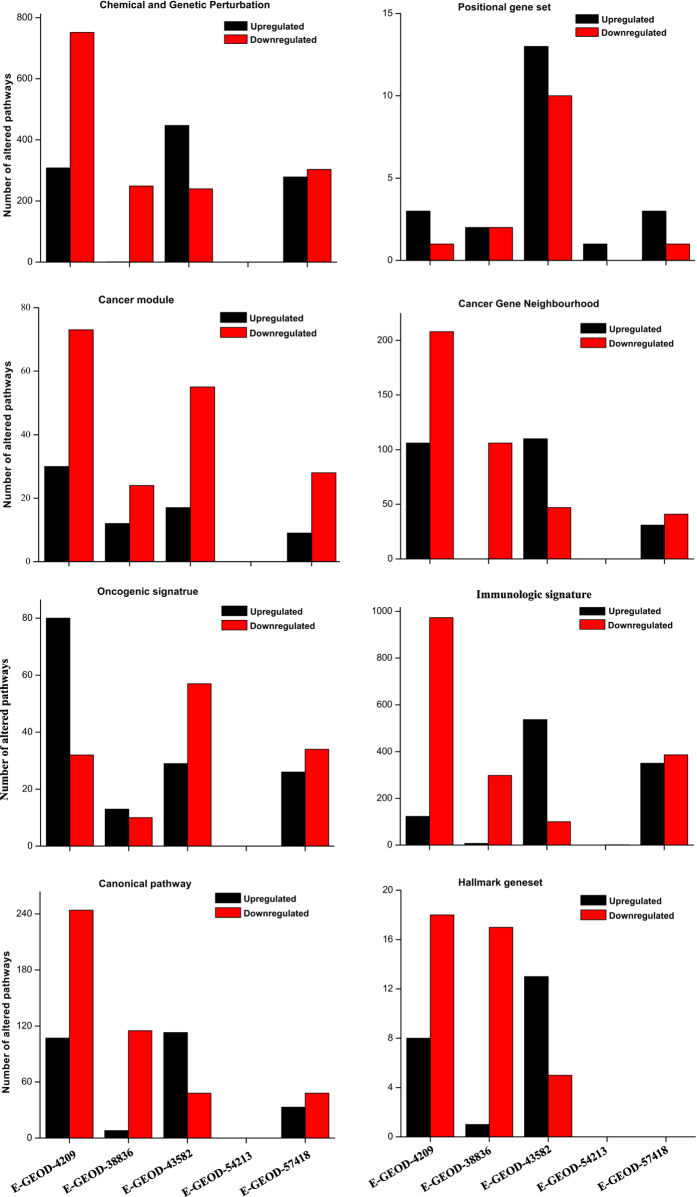
Number of statistically significant (p < 0.001, q < 0.001) altered molecular pathways identified by GSEA on different studies. Each bar chart represents one of the eight gene set modules from molecular signature database (MSigDB).

During microgravity exposure, a large number of molecular pathways from each study are found to be upregulated or downregulated in a statistically significant way (p < 0.01, q < 0.25) (Fig. 2). A total number of 1976, 560, 1224, 0, and 836 pathways were altered spanning all the modules for E-GEOD-4209, E-GEOD-38836, E-GEOD-43582, E-GEOD-54213 and E-GEOD-57418 respectively. We have excluded E-GEOD-54213 (unpublished data) from main analysis, as it shows no statistically significant altered pathway. GSEA shows ability to extract more pathways with statistical confidence than differential gene expression analysis. For example, reference^14^ (E-GEOD-43582) identifies only 8 KEGG pathways without any quantitative confidence. Using GSEA we have identified total 45 altered KEGG pathways (26 upregulated and 19 downregulated) with p < 0.05 and high normalized enrichment score (NES) value (see Supplementary Table S1 for details). Further, we have analysed thousands of pathways from various modules (cancer modules, oncogenic signature, chemical and genetic perturbation etc.), which were not analysed before. More than 98% of those identified pathways are found to be new for each datasets compared to the corresponding published results. Thousand of pathways are altered (Fig. 2) in our analysis whereas tens of pathways are reported in previous publications^14^,^15^,^18^,^20^.

**Figure 3:**
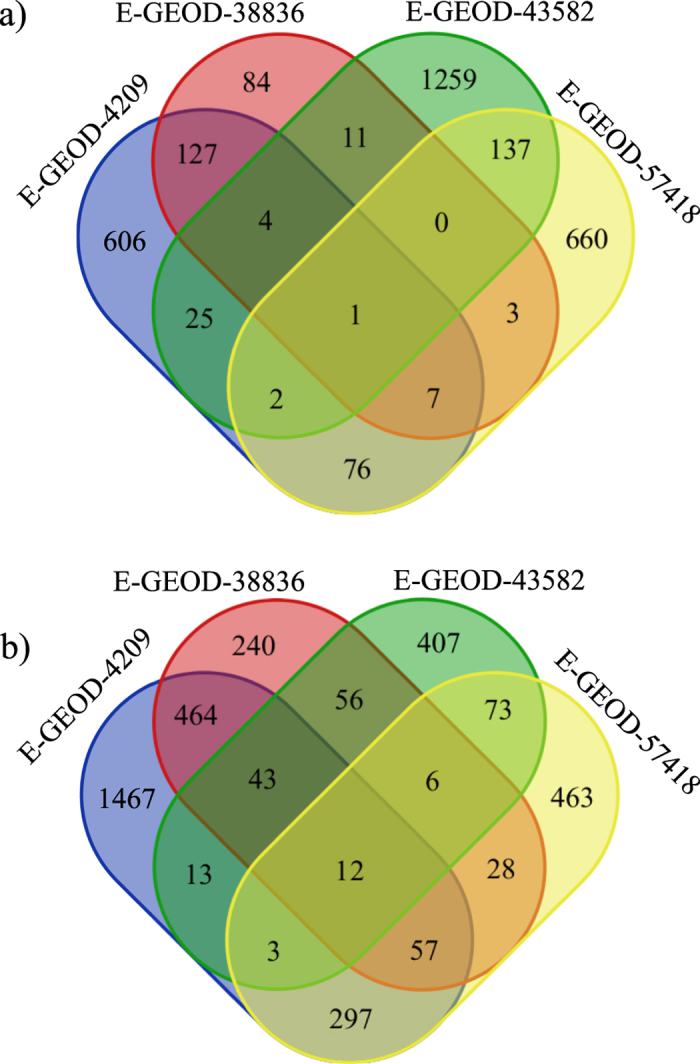
Venn diagrams represent the common altered pathways in microgravity for (**a**) upregulated pathways and (**b**) downregulated pathways. All altered pathways from 8 gene set modules are combined in this diagram.

Next, we have compared the GSEA identified pathways from all 4 datasets and identified more than 100 altered pathways (high NES values and p < 0.05), which overlapped among at least three data sets (Fig. 3 and Supplementary Table S2). Around 100 of those overlapped pathways were identified as novel molecular signatures, which were not reported in any of those source publications (see Supplementary Table S2, the novel pathways found were are marked as ‘This Study Only’). The short description of all the overlapping pathways can be found in Supplementary Table S3. We further classified those overlapped pathways based on specific immunity and disease related functions. All those functions and their molecular signatures (pathways) are tabulated in Table 1.

### Novel molecular signatures related to immunity

Those novel, overlapping pathways can be divided in two categories. In first category, the earlier reported pathways in one study are found in other datasets, where the pathways were never reported. Reference^15^ showed that one of the reasons for impaired immunity was the downregulation of tumour necrosis factor (TNF) mediated NF- κB /Rel pathways. We have identified the downregulation of the same pathway (appeared as Hallmark_TNFA_signaling_via_NF- κB in our analysis, Table 1 and Supplementary Table S2) by analysing data of reference^15^. In addition, we identified the same pathway in two other studies E-GEOD-43582^14^ and E-GEOD-4209^20^, where this pathway was not reported. Similarly, the pathway responsible for generation of second messenger molecules in T-cell regulation (TCR) signalling (Reactome second messenger molecules signalling pathway, Table 1 and Supplementary Table S2) is repressed in microgravity for all four studies. However, only one study^18^, (E-GEOD-57418) suggested the same without any statistical confidence. Those messenger molecules are associated with the activation of both NF- κB and PKC pathways along with calcium mobilization.

In the second category, we found completely new pathways and class of functions, which were not reported in any of the studies with source datasets. We identified the downregulation NF- κB pathway via Notch1 signalling (Vilimas Notch1 targets up, Table 1 and Supplementary Table S2) as a new route for NF- κB deregulation. In addition we have found another new pathway, the downregulated genes of LPS induced stimulation of DC macrophage (Zhou inflammatory response live up, Table 1 and Supplementary Table S2), as an associated signature. It was shown that Notch1 mediated NF-κB pathway induced LPS stimulated macrophage activation^30^. We found 2 completely new pathways, which could be seen as associated molecular signatures in connection with the reduced second messenger molecules. The, reduced allograft rejection (KEGG allograft rejection, Table 1 and Supplementary Table S2) and reduced NFAT (nuclear factor for activated T cell) pathway (PID_NFAT_TF pathway, Table 1 and Table S2) in our analysis are most likely connected with the downregulation of the second messenger molecules, as blocking of this pathway prevents graft rejection in transplanted patients^31^ and calcium mobilization is required for NFAT activation^32^.

To exclude the possibility that this new route (Vilimas Notch1 targets up) for NF- κB pathway deregulation appeared in our analysis is due to the presence of common genes from Hallmark_TNFA_signaling_via_NF- κB and Reactome generation of second messenger molecules, we compared the leading edge genes of three pathways for each of the experiments. The results showed a negligible overlaps (Supplementary Fig. S1).

We have identified reduced autoimmunity and LPS induced gene expression as two new classes of signature in microgravity. Those functional classes are supported by multiple novel pathways (Table 1). Further we have found multiple novel and specific signatures, which suggests the reduced inflammatory response as a functional class (Table 1). We have also identified repressed intestinal immune network for immunoglobulin A (IGA) production and cytokine-cytokine receptor interactions, which were not reported before in the context of microgravity. Several other signatures for reduced immunity were also observed. A comprehensive list is tabulated in Table 1.

### Novel molecular signatures related to cancer

Radiation induced cancer risks (and death risk) for astronauts in international space station were estimated for various cancers including breast cancer, leukaemia and lung cancer^33^. Though experiments showed alternation of microRNAs related to cancers in microgravity as discussed in the introduction, till now, no microgravity study on healthy human cells showed induction of cancer signature in gene expression study. We were curious whether there is any cancer signature is embedded in the gene expression data, which were obscured in conventional analysis. We found strong signature on induction of liver cancer and leukaemia as evident by several molecular pathways (under cancer in Table 1). In addition we have identified signatures related to lung cancer, breast cancer, ovarian cancer and head and neck cancer (under cancer in Table 1). We have also found induction of oncogenic KRAS signalling pathway (KRAS.300_UP.V1_UP and KRAS.600_UP.V1_UP, Table 1 and Supplementary Table S2). In space, there is always a possibility of radiation exposure even within ISS or in-flight experiments. Therefore, those cancers related signatures might not be unlikely, where the cells were studied in space (E-GEOD-38836 and E-GEOD-43582). However, it is intriguing that the cell cultured in earth based microgravity simulator (E-GEOD-4209 and E-GEOD-57418) without any radiation exposure also showed those cancer signature in our analysis (Supplementary Table S2). Thus our study suggests that microgravity alone may induce several cancer related signatures. Further, we have found the signatures about increased drug response on B lymphoma, liver cancer, breast cancer and non-small lung cancer in microgravity (Table 1).

### Other novel molecular signatures

Our results showed reduction of diabetic signature through multiple gene sets, which includes the upregulation of the repressed genes in pancreatic islet upon HNF1A knockout (Servitja islet HNF1A targets dn, Table 1) and downregulation of type I and type II diabetic genes (KEGG Type I diabetes mellitus, GSE9006 healthy Vs type 2 diabetes, Table 1). Similarly reduced asthma, blood differentiation (under other diseases, Table 1) and post-transcription processes are also identified (please see under cellular and metabolic pathways in Table 1) as new signatures.

### Consensus non-negative matrix factorization (CNMF) clustering and protein network mapping show plausible regulatory and functional connections among genes

**Figure 4:**
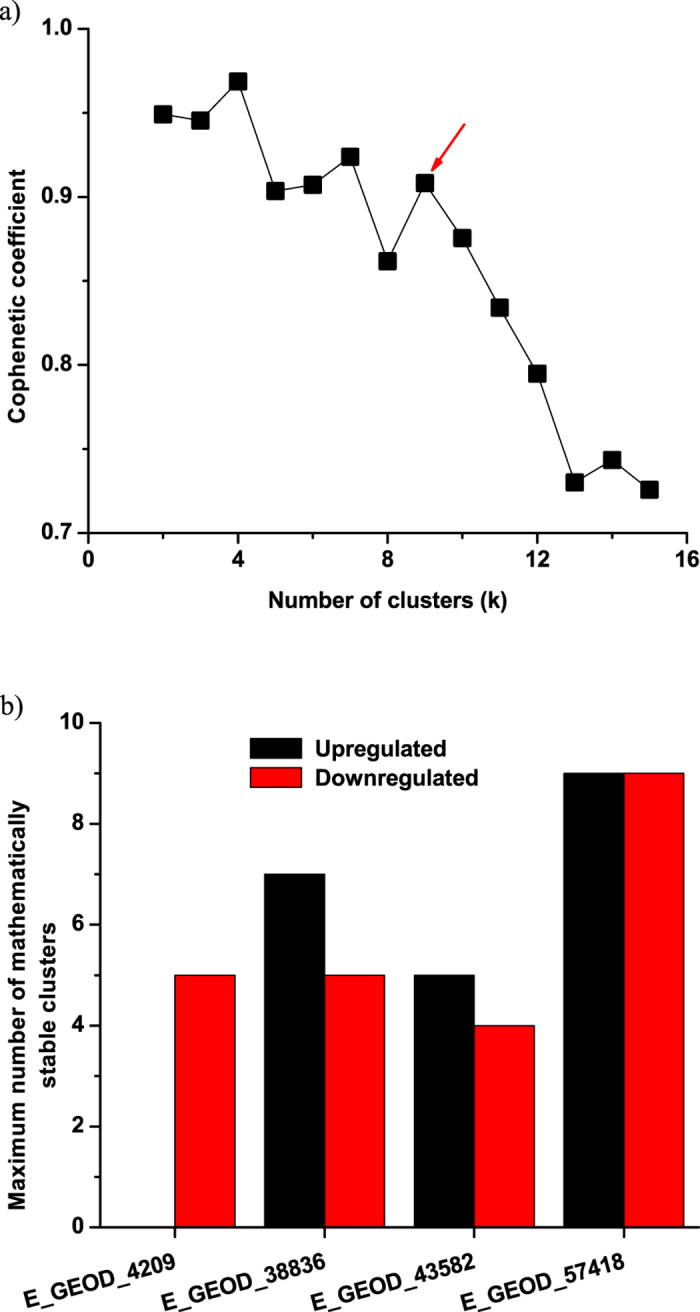
Consensus non-negative matrix factorization (NMFC) clustering on leading edge genes. (**a**) Cophenetic coefficient as a function of number of clusters for downregulated leading edge genes from canonical pathways for E-GEOD-38836. The arrow indicates the maximum number of mathematically stable clusters possible in this example. (**b**) Maximum number of mathematically stable clusters plausible for other experiments. Clusters with upregulated and downregulated genes are shown in black and red bars respectively.

**Figure 5:**
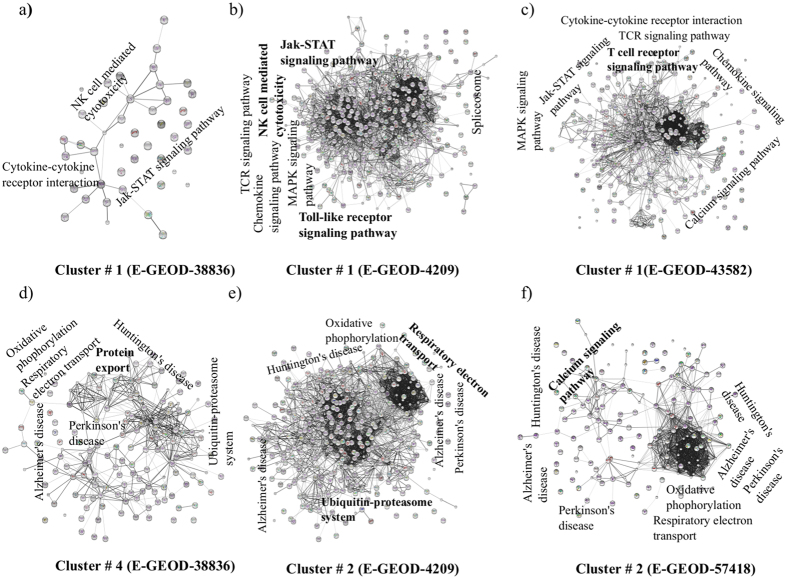
Protein-protein association (PPA) networks. Networks (**a–f**) represent NMFC clusters from various datasets of downregulated genes from canonical pathways in microgravity. Each node represents a protein and the line connecting the nodes (edge) represents the functional association. The relative thickness of each line signifies the confidence level of such association.

To understand the regulatory or functional networks among the leading edge genes, we have clustered them based on their expression patterns using an unsupervised non-negative matrix factorization (NMF)^25^, coupled with a consensus clustering (NMFC)^26^. NMFC is applied on the combined leading edge genes from upregulated and downregulated gene sets separately, from canonical pathway modules. The maximum number of mathematically stable clusters was estimated from the cophenetic coefficient as a function of number of clusters. Figure 4a shows one of such plots for upregulated leading edge genes from E-GEOD_57418. In this figure, a sharp and continuous decline is observed after the number of clusters exceeds 9 (shown by arrow). This number can be taken as the maximum possible number of mathematically stable clusters. The stable numbers of clusters for each experiment are shown in Fig. 4b and cophenetic plots for each experimental condition are shown in Supplementary Fig. S2. The genes from each stable cluster are further mapped on the STRING protein network database. The resultant networks showed densely interactive and localized set of protein-protein interactions. Those interactions were enriched with KEGG and Reactome pathways (the two constitutive member databases in Canonical Pathways in MSigDB) with a built in tool in the STRING. Enrichment with p value < 10^−5^ were considered for further analysis. Figure 5 represents a set of such PPA networks. The functional annotations with statistical parameters of all the networks are tabulated in Supplementary Table S5.

The small but tightly connected PPA networks within a cluster suggest regulatory and/or functional connections among genes and provide more specific and detailed functionality. In PPA networks, we mainly observed i) distributed functions, where same functions are distributed among multiple clusters for an experiment (dastaset) and ii) unique functions, which are confined only within a specific cluster for a dataset. Example of unique functions include Neurotrophin signalling pathway, respiratory electron transport and activation of claspin, which are only found in cluster 1, 2 and 4 respectively, among all the 5 downregulated clusters from E-GEOD-4209 (Supplementary Table S5). All unique and distributed functions can be found in Supplementary Table S5. The appearance of unique functions among clusters, which resulted from unsupervised clustering, suggests a set of genetic networks respond to microgravity with distinct gene expression patterns. As cell types and experimental conditions are different for the datasets, the functional enrichment of clusters differs among experiments. However, the association of the similar functions, among multiple experiments is of particular interest as they indicate towards plausible regulatory connections in response to microgravity. There are few instances in our results, which demands further attention.

We have already mentioned that GSEA identifies upregulated olfactory transduction pathway in microgravity. However, PPA networks pinpoint it as olfactory receptor G protein trimer complex, which appeared in upregulated clusters of multiple experiments (cluster#6 E-GEOD-38836, cluster#1 and 4 E-GEOD-43582, Upregulated Canonical Pathways, Supplementary Table S5), Next, a network level association between Jak-STAT, cytokine-cytokine receptor interaction, TCR signalling pathway, NK cell mediated cytotoxicity and chemokine signalling is appeared in multiple datasets (Fig. 5a–c).

Immunology studies, not related with microgravity, showed that the cytokine-cytokine receptor interaction and Jak-STAT signalling pathway are directly connected through a cascade of biochemical reaction^34^. Hyper -activation of this cascade results inflammation and asthma^34^. It is interesting that this proportional relation is observed in our analysis and those disease pathways are found to be downregulated. This functional network interface was never reported in the context of microgravity. Further, a close association among oxidative phosphorylation/respiratory electron transport, neurodegenerative conditions (Alzheimer’s disease, Huntington’s disease and Parkinson’s disease) and ubiquitin-proteasome system are appeared in clusters of three experiments (Fig. 5d–f). Oxidative phosphorylation/respiratory electron transports are shown related with neurodegenerative condition^35^ and with ubiquitin-proteasome system^36^ but togetherness of those three functions with similar gene expression patterns never reported in response to microgravity. Thus the interface among various functions (Fig. 5a–f) within a genetic network may guide us towards better understanding how microgravity influence several of the cellular functions through a small set of genes and warrant further investigations.

## Discussion

An important aspect of this pipeline is to identify microgravity induced pathways with statistical confidence and presence of multiple signatures for a functional class. In this way the reduction of generation of second messenger molecules, which was merely suggested by one study^18^, appeared as a statistical significant pathway in all the 4 datasets in our analysis along with multiple new pathways as associated molecular signatures (shown in the result section). Microgravity generates low shear stress around the cells in liquid media^37^,^38^. It was particularly found that low shear stress (<1 dynes/cm^2^) reduces the amount of second messenger molecule IP3 by 20%^39^ and also influence the Ca^2+^ mobilization^40^ in mammalian cells. Therefore, the inhibition of immune systems may directly originate from microgravity induced low sheer stress on the cells through the reduced amount of second messenger molecules (all 4 studies showed this new pathway in our analysis), which negatively influence the NF-κB pathway. In addition, the PPA network shows the reduction of Jak-STAT signalling pathway (Fig. 5a–c), which has a proportional relationship with fluid shear stress^41^. This reduction may directly be related with the reduced inflammation and asthma through cytokine-cytokine receptor interaction^34^ and all of those pathways were found downregulated in our results. Based on this, we hypothesize that the low shear stress in microgravity is responsible for partially impaired immunity.

Our new results support functional observations from other studies, where no gene expression was performed and shed some light on couple of microgravity-induced issues, where the genetic basis is still unknown. This analysis showed reduction of the several LPS induced gene expression signature (See Table 1 under Reduction in LPS induced gene expression and immunogenic signature in Supplementary Table S2). Several of the cytokines production was shown reduced after LPS stimulation in microgravity^8^. This analysis showed the presence of several signatures of autoimmune repression (Table 1), which were never reported. A recent study speculated microgravity could be a measure for fighting autoimmune disease^9^. Further, tissue engineering model in microgravity showed the enhanced survival, better secretory profile and better insulin normalization of beta islet cells compare to ground control^42^. Several of the reduced diabetic signatures are appeared in our results (Table 1). The change in the smelling behaviours among astronauts in space was documented and attenuation of olfactory components as a result of microgravity induced upward shift of body fluid was postulated as plausible reason^43^. Our results identify the upregulation of olfactory signal transduction pathways, suggesting molecular genetic origin of changed smelling behaviour in space.

Apart from the new and overlapping pathways, we have showed that how gene expression patterns are possibly connected with functional behaviour at microgravity. An emphasis was given to extract similar types of gene expression patterns across the dataset. To our knowledge, this is the first study in this direction. We were able to show i)association between Jak-STAT, cytokine-cytokine receptor interaction, TCR signalling pathway, NK cell mediated cytotoxicity and chemokine signalling and ii) a close association among oxidative phosphorylation/respiratory electron transport, neurodegenerative conditions (Alzheimer’s disease, Huntington’s disease and Parkinson’s disease) and ubiquitin-proteasome system across multiple datasets (see result section for details). We have shown from the literature that few of those pathways are connected in human cells but never reported in context of microgravity. Those patterns (cluster/gene network) in gene expression are derived from unsupervised mathematical analysis without imposing any prior functional knowledge. Thus the identification of common functional interfaces from those gene networks across multiple data-sources suggests the plausible functional and regulatory connections among genes in microgravity and how microgravity may influence several functions through a small set of genes.

In this analysis the data sets were taken for 3 different cell types. Microgravity may work differently on different cell types and experimental conditions. This is evident in our results as major fraction of the pathways altered for each dataset are non-overlapping (Fig. 3). However, the question remains that what could be the minimum set of cellular pathways, if any, influenced by the microgravity irrespective of the cell types. Our results shed some light in this aspect. Though the four studies were performed in different experimental conditions with different cell types, microgravity conditions (simulated and spaceflight), media, fluid shear around cells, hydrostatic pressure, temperature, aeration, reactor and different experimental groups, around 100 pathways were overlap among alteast 3 (out of 4) experiments. Apart from the overlapping pathways, we further identified, across the datasets, common interface of multiple functions in gene networks. Those gene networks were derived from mathematical pattern recognition (unsupervised clustering) without any prior functional knowledge. Thus the common functional interface within a gene network (cluster) across multiple datasets, suggest that the overlapping pathways identified from various cell types are not arbitrary. Those examples atleast suggest that multiple pathways are perturbed in microgravity by similar way, irrespective of cell types. However, similar pathway may not lead to the similar functions in all cell types and should be validated by functional experiments. The previous studies mostly suggested the microgravity induced pathways without statistical confidence or a set of broad functional categories missing the specific details. This prevents to pinpoint a pathway from several others and the details for future experimental design. Our analytical pipeline has generated a set of specific and testable hypotheses, which are subjects of near future experimentation. The analysis predicts the i) reduction of second messenger molecules as a fundamental reason for T cell regulation dysfunction and ii) the increased effectiveness of the drugs (specifically Tamoxifen and Rapamycin, see Becker Tamoxifen resistance up; Peng Rapamycin response dn under cancer in Table 1) on cancers in microgravity. Two important future experiments may include i) measuring T cell regulation in T cells as a function of amount of second messenger molecules and the associated gene expression in microgravity and ii) the effect of drugs on various cancer cell types in microgravity. Further our results suggest that in microgravity, the mRNA processing gets partially impaired (Table 1), which can be tested on varieties of cell types to understand the effect of microgravity on fundamental cellular processes.

## Conclusion

Here we represent an analytical pipeline, which gives an integrated molecular systems level picture of healthy human cells under microgravity. Adequate cellular and human level data for assessing the health risks for interplanetary travel are not available till date^3^. Enough human level data may not be available in near future as the number of astronauts flown to space is low. Therefore, assessment of health risks from molecular signatures may serve as a key criterion for future manned space mission. In addition, experiments in space are costly business. Therefore, getting maximum level of insight from a space experiment is of a crucial importance. Our approach identifies statistically significant pathways directly from the gene expression data, even in the low fold change regime, which cannot be possible in conventional differential gene expression analysis followed by passive pathway mapping. The pan molecular pathways analysis, encompassing almost all known aspects of human disease and immunity, followed by comparative analysis identifies a set of new altered molecular signatures, which are appeared across studies. Most functions are supported by multiple pathways and associated (downstream) molecular pathways. Thus it gives a high degree of confidence. Further, unsupervised clustering suggests the plausible regulatory connections among altered genes in microgravity. Our results suggest a set of specific hypotheses, which can be tested directly in an earth based microgravity simulator or in space flight condition and may help assessing risks and developing new medicine for microgravity induced health hazards.

## Methods

### Gene set enrichment analysis (GSEA)

GenePattern, a platform for multiple genomic analyses was used for performing Gene Set Enrichment Analysis (GSEA). The module was run with global gene expression datasets in an appropriate file (.GCT) format. A separate file (.CLS) was developed to differentiate the gene expression data between 1g and microgravity. The molecular pathways from MSigDB are linked with GenePattern server. First we generated a ranked list of genes (Fig. 1a) by applying ‘signal-to-noise’ metric, which can be obtained by (A_microg_ − A_1g_)/ (SD_microg_ + SD_1g_). Here, A_microg_, A_1g_ represent the average expression of genes in microgravity and normal gravity condition, respectively. SD_microg_ and SD_1g_ represent standard deviation associated with microgravity and normal gravity gene expression data. A running sum statistics (GSEA algorithm) with a weighted Kolmogorov-Smirnov like scoring scheme^21^ was applied on the ranked list to determine if the genes from a gene set were distributed either top or the bottom of it. This statistics estimates a score (enrichment score or ES) for each gene set, which increases when a gene from a gene set hits the same gene in the ranked list. The score is evaluated by running down the ranked gene list (RGL) and if genes from a particular gene set (GSet) gives positive hit in the RGL, the score would increase, otherwise decrease. The probabilities of positive hit and negative hit (miss) can be estimated from the following equations.


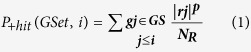



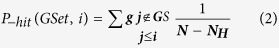


where, g_j_ denotes the jth gene in RGL = {g_i_, …, g_N_}, r(g_j_) = r_j_ p = 1 and


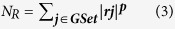


The highest deviation of P_+hit_ − P_−hit_ from ‘0’ gives the estimation of enrichment score (ES). The GSet permutation type was set to a value of 1000, i.e. the GSets would be permuted randomly 1000 times among all gene sets (molecular pathways) to estimate the statistical significance (null hypothesis testing). The false discovery rate (FDR-q value) was estimated from the distribution of the p values. GSEA analysis was run for atleast three times to check the consensus in ES, NES, p value and q value among various runs. The molecular pathways in MSigDBare manually checked in detail for its interpretation in the context. One category in MSigDB is cancer modules. Every module is related with its clinical annotation through its module map in (http://robotics.stanford.edu/~erans/cancer/index.html). For each of the module, we have assigned the clinical annotation, when it is highly significant (p < 10^−10^) and when the minimum hit of the module genes on cancer pathways is atleast 50%.

### Leading edge gene analysis

Each common statistically significant pathway for individual experiments also tells about the genes, which are most significant (high NES value) in a given pathway. The genes before the peak (highest NES value) are the leading edge genes. Those genes were extracted from the GSEA results and tabulated for further analyses.

### Clustering of leading edge genes with non-negative matrix factorization consensus (NMFC)

The consensus non-negative matrix factorization (NMFC) clustering is run in GenePattern. It requires only positive entry in gene expression data table. Therefore we checked for negative elements on each expression sets for NMF and we found none. The expression data of leading edge genes combining all statistically significant gene sets from canonical pathways, which is a collections of 1330 gene sets in MSigDB, were chosen for NMFC run. The upregulated and downregulated leading edge genes have been run separately. Based on the gene expression patterns of the multiple cell samples between microgravity and normal gravity, NMF clusters the genes. NMF consider the gene expression dataset as a positive matrix M of the size R X C, with N number of clusters. Then its iteratively computes for matrices Y and Z so that M = YZ, with Y X N and N X Z sizes. In each step, iteration was updated to find a minimum in an appropriate function. The details of the NMF can be found in refs 25,26. The iteration number per clustering was set as 2000 times. On top of the NMF, a consensus clustering was run to evaluate the mathematical stability of the NMF clusters. We sequentially ran the consensus clustering by setting the number of clusters from 2 to 20. The cophenetic coefficient associated with the each distribution of the clusters was estimated and the rate of the change of this coefficient estimated the highest number of plausible stable clusters. The cluster, after which the cophenetic coefficient drops sharply, denotes the maximum possible number of mathematically stable clusters. The associated genes for each stable clusters were tabulated for further analysis.

### Protein-protein associated networks of clustered genes

The genes from each stable cluster were mapped on STRING network database (version 9.1) to study protein-protein association networks. The list of genes from each clusters were uploaded and assigned *Homo sapiens* as target organism on STRING. Further the enrichment score and associated p values for functional annotation from KEGG and Reactome pathway database were determined from the in-built functional tools. As KEGG and Reactome databases are common in STRING and canonical pathways in MSigDB, we have chosen canonical pathways for NMFC.

## Additional Information

**How to cite this article**: Mukhopadhyay, S. *et al*. A systems biology pipeline identifies new immune and disease related molecular signatures and networks in human cells during microgravity exposure. *Sci. Rep.* **6**, 25975; doi: 10.1038/srep25975 (2016).

## Supplementary Material

Supplementary Information

## Figures and Tables

**Table 1:** Microgravity induced functions are supported by multiple molecular pathways.

**Function**	Supporting up-regulated gene-sets*	Supporting downregulated gene-sets*
**Immunity**		
Repressed immunity		KEGG intestinal immune network for IGA production; Biocarta inflame pathway; Biocarta cytokine pathway; KEGG cytokine cytokine receptor interaction; PID NFAT TF pathway; Zhou inflammatory response live up; Vilimas Notch1 targets up; Goldrath antigen response; Hallmark TNFA signaling via NFκB; GSE17721 0.5 h Vs 4 h CPG BMDM up; GSE3982 B cell Vs EFF memory CD4 T-cell up;Biocarta NKT pathway; Reactome second messenger molecules signaling pathway
Reduced inflammatory response		Biocarta inflame pathway; Galindo immune response to enterotoxin; Tian TNF signaling not via NFκB; Seki inflammatory response LPS up; GSE9988 LPS Vs LPS and anti TREM1 monocyte dn; GSE14000 unstim Vs 16 h LPS DC up
Repressed autoimmunity signature		KEGG allograft rejection; KEGG autoimmune thyroid disease; KEGG graft Vs host disease; KEGG TypeI diabetis mellitus; Reactome PD1 signaling
Reduction in LPS induced gene expression		GSE9988 LPS_Vs vehicle treated monocyte up;_GSE2706 unstim Vs 2 h LPS DC dn; GSE9988 low LPS Vs vehicle treated monocyte up; GSE9988 low LPS Vs ctrl treated monocyte up; LOW_LPS_VS_CTRL_TREATED_MONOCYTE_UP, GSE9988_LPS_VS_CTRL_TREATED_MONOCYTE_UP, SEKI_INFLAMMATORY_RESPONSE_LPS_UP
Blood cell differentiation signature (inhibition)		Mori mature B lymphocyte up; Oswald hematopoetic stem cell in collagen gel up; Basso CD40 signaling up
**Cancer**		
Induced liver cancer		Module 75; Module 46
Induced B Lymphoma, diffuse large B cell lymphoma, leukaemia	Module 47	Module 6; Module 123; Verhaak AML with NPM1 mutated dn
Induced Lung carcinoid and reduced pro-survival		Phong TNF tergets up
ESR positive breast cancer	Doane breast cancer ESR1 dn	
Induction of Head and Neck cancer	Rickman head and neck cancer C	
Oncogenic signature	KRAS300_UP.V1_UP; KRAS600_UP.V1_UP	Amit delayed early genes
Ovarian cancer growth		Lu EZH2 targets up
Increased responsiveness to cancer treatment	Heller HDAC Targets silenced by methylation up	Kobayashi EGFR signaling 6 hr dn; Becker Tamoxifen resistance up; Peng Rapamycin response dn, Lee liver cancer survival dn
Induction of apoptosis		GSE37416 CTRL Vs 12 h F Tularessis L Vs neutrophil dn; Brocke apoptosis reversed by IL6; Dairkee TERT targets up
**Other disease signatures**		
Reduced asthma signature	Bosco epithelial differentiation module	KEGG asthma
Reduced diabetes signature	Servitja islet HNF1A targets dn	KEGG Type I diabetes mellitus; GSE9006 healthy Vs type 2 diabetes PBMC at DX up
Psychiatric		Kim all disorders duration corr dn; Stark prefrontal cortex 22Q11 deletion dn
**Cellular and metabolic pathways**		
Reduced oxidative phosphorylation		Reactome TCA cycle; Reactome respiratory electron transport; ATP synthesis by chemiosmotic coupling and heat produced by uncoupling protein; Module152; Mootha VOXPHOS; Hallmark oxidative phosphorylation
Reduced post transcription		Module 114; Reactome mRNA processing; LI DCP2 bound mRNA
Increased SLC-mediated transmembrane transport	Reactome SLC mediated transmembrane transport	

^*^The description of the gene sets can be found in Supplementary Table 2.
