# Supplementary material for: A systems biology pipeline identifies new immune and disease related molecular signatures and networks in human cells during microgravity exposure
Source: Sci Rep. 2016 May 17;6:25975. doi: 10.1038/srep25975 (PMC4868995; doi:10.1038/srep25975)
Supplement: Supplementary Information [file srep25975-s1.pdf]

Supplementary Information

A systems biology pipeline identifies new immune and disease related molecular signatures and networks in human cells during microgravity exposure

Sayak Mukhopadhyay[1,a], Rohini Saha[1,a], Anbarasi Palanisamy [1], Madhurima Ghosh[1], Anupriya Biswas[2], Saheli Roy[2], Arijit Pal[1], Kathakali Sarkar[1]and Sangram Bagh[1]*

1 Biophysics and Structural Genomics Division, Saha Institute of Nuclear Physics, Kolkata, 700064, India
2 Department of Biological Sciences, Presidency University, Kolkata, 700073, India
a These authors have contributed equally to this work

* To whom correspondence should be addressed
Sangram Bagh, PhD
Biophysics and Structural Genomics Division
Saha Institute of Nuclear Physics
Sector-I, Block-AF, Bidhannagar
Kolkata 700064, India
Phone: (+91)-33-2337-5345-49 Ext: 4625
E-mail: sangram.bagh@saha.ac.in

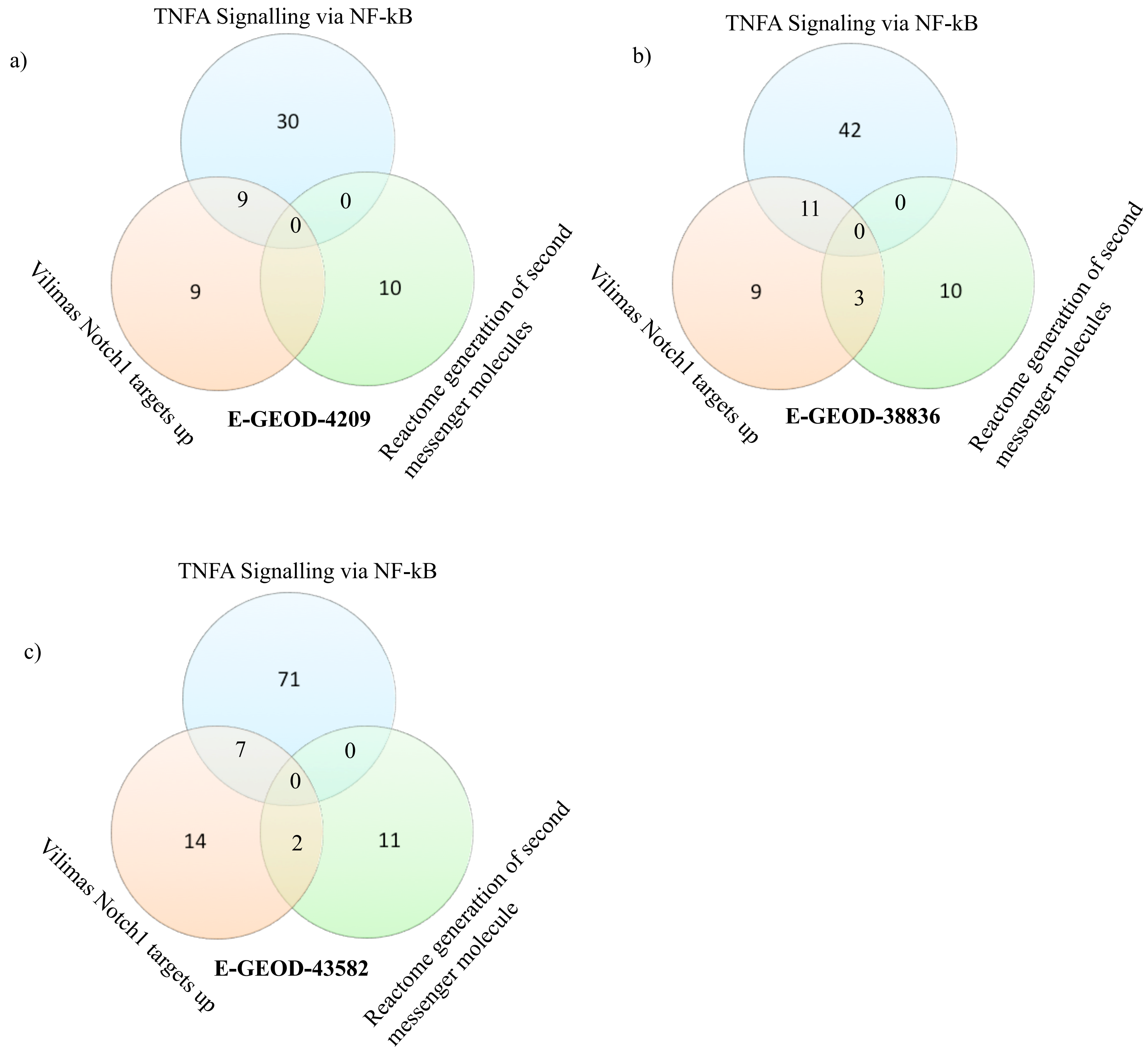

Supplementary Figure S1. Overlapping leading edge genes between three pathways, which affect the regulation of NF-kB pathway.

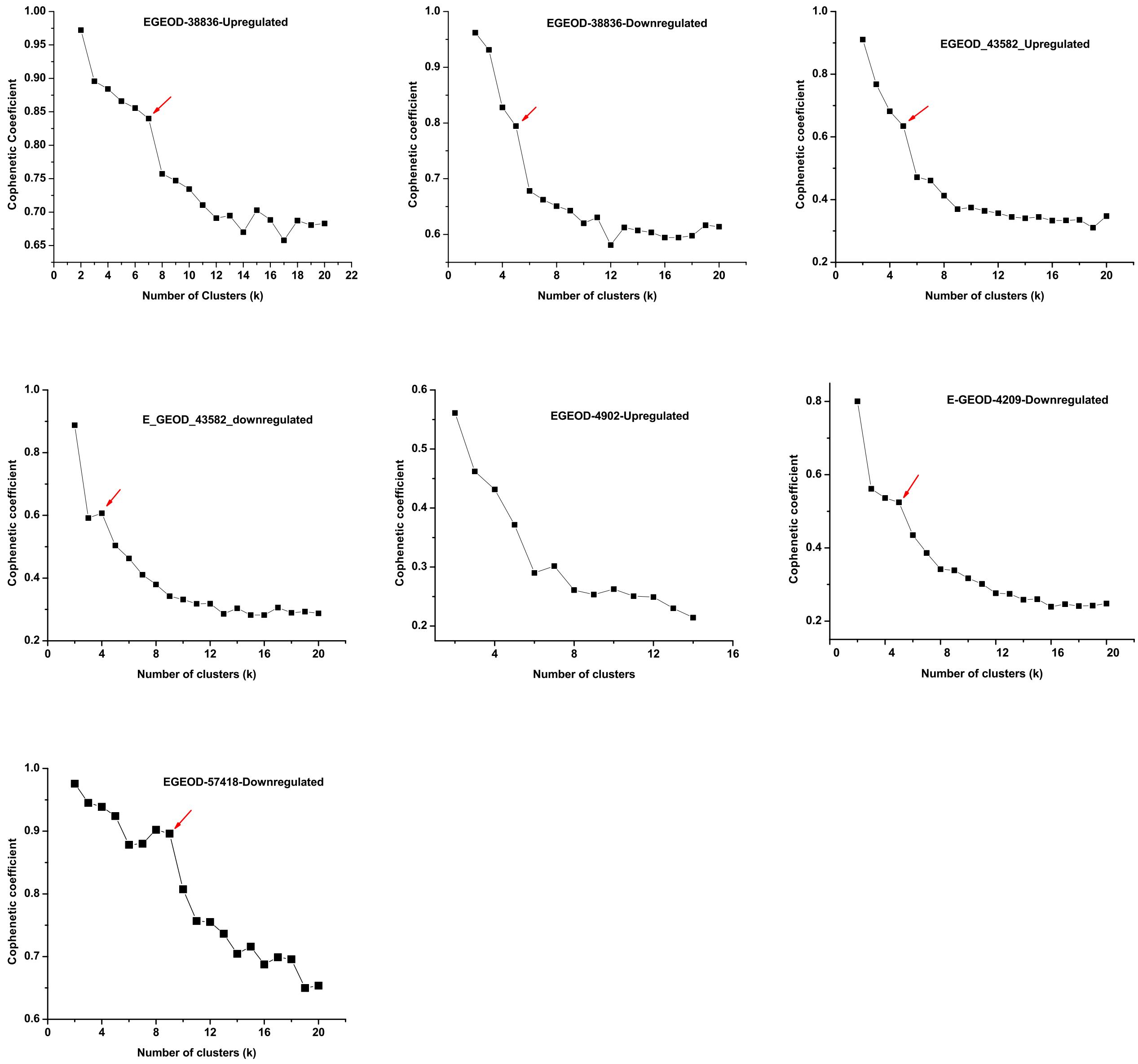

Supplementary Figure S2. Cophenetic coefficient as function of number of clusters. The maximum possible number of mathematically stable clusters is indicated with an arrow in each plot.

Suplementary Table S1. GSEA with KEGG

Upregulated KEGG pathways in E-GEOD-43582 by GSEA	NES	p value	q value
KEGG_ABC_TRANSPORTERS	1.5371544	0.02841918	0.15972634
KEGG_AMINOACYL_TRNA_BIOSYNTHESIS	1.5919701	0.01067616	0.11897065
KEGG_ASCORBATE_AND_ALDARATE_METABOLISM	1.5905842	0.03272727	0.11896445
KEGG_BASAL_TRANSCRIPTION_FACTORS	1.6450139	0.01457195	0.08438345
KEGG_BASE_EXCISION_REPAIR	1.6883452	0.01230229	0.06502499
KEGG_CELL_CYCLE	2.0528526	<0.001	0.00201357
KEGG_DNA_REPLICATION	2.4068143	<0.001	<0.0001
KEGG_GLYCEROLIPID_METABOLISM	1.4472281	0.04145078	0.22810024
KEGG_GLYCOSYLPHOSPHATIDYLINOSITOL_GPI_ANCHOR_BIOSYN	1.9903554	0.00177305	0.00401596
KEGG_HOMOLOGOUS_RECOMBINATION	2.111701	<0.001	7.17E-04
KEGG_LYSOSOME	1.3951455	0.03276131	0.27994174
KEGG_MISMATCH_REPAIR	2.1678996	<0.001	2.13E-04
KEGG_N_GLYCAN_BIOSYNTHESIS	1.7969545	<0.001	0.02778414
KEGG_NUCLEOTIDE_EXCISION_REPAIR	2.1792207	<0.001	1.92E-04
KEGG_OLFACTORY_TRANSDUCTION	1.3613214	0.00994318	0.30722424
KEGG_ONE_CARBON_POOL_BY_FOLATE	1.48253	0.04963235	0.1981213
KEGG_OOCYTE_MEIOSIS	1.6564622	0.00168919	0.07677514
KEGG_P53_SIGNALING_PATHWAY	1.4092189	0.04676259	0.26277024
KEGG_PENTOSE_AND_GLUCURONATE_INTERCONVERSIONS	1.7792071	0.00561798	0.03050728
KEGG_PEROXISOME	1.6430537	0.00344234	0.0846228
KEGG_PROGESTERONE_MEDIATED_OOCYTE_MATURATION	1.5459824	0.00991736	0.15496084
KEGG_PYRIMIDINE_METABOLISM	1.4103856	0.0247117	0.26421168
KEGG_SELENOAMINO_ACID_METABOLISM	1.6695464	0.00948767	0.07186186
KEGG_STARCH_AND_SUCROSE_METABOLISM	1.7428765	0.00357143	0.04269232
KEGG_TASTE_TRANSDUCTION	2.3500557	<0.001	<0.0001
KEGG_UBIQUITIN_MEDIATED_PROTEOLYSIS	1.7050434	0.00162602	0.05871949
**Downregulated KEGG pathways in E-GEOD-43582by GSEA**			
KEGG_ALLOGRAFT_REJECTION	-1.9176508	<0.001	0.05072754
KEGG_ARACHIDONIC_ACID_METABOLISM	-1.6956009	0.00230415	0.1281111
KEGG_ASTHMA	-1.933172	0.00226244	0.04988514
KEGG_AUTOIMMUNE_THYROID_DISEASE	-1.8415047	<0.001	0.06386254
KEGG_CALCIUM_SIGNALING_PATHWAY	-1.5539435	<0.001	0.19537006
KEGG_CARDIAC_MUSCLE_CONTRACTION	-1.6792786	<0.001	0.1291458
KEGG_CHEMOKINE_SIGNALING_PATHWAY	-1.4417932	0.0056338	0.3102306
KEGG_CYTOKINE_CYTOKINE_RECEPTOR_INTERACTION	-1.8166839	<0.001	0.06323686
KEGG_DILATED_CARDIOMYOPATHY	-1.5368422	0.00501253	0.21578678
KEGG_FRUCTOSE_AND_MANNOSE_METABOLISM	-1.488362	0.03009259	0.26286906
KEGG_GRAFT_VERSUS_HOST_DISEASE	-1.4312685	0.04608295	0.3212639
KEGG_HEMATOPOIETIC_CELL_LINEAGE	-1.634285	0.00260417	0.14568602
KEGG_HYPERTROPHIC_CARDIOMYOPATHY_HCM	-1.6253177	0.00724638	0.1509699
KEGG_INTESTINAL_IMMUNE_NETWORK_FOR_IGA_PRODUCTION	-1.8449609	0.00232019	0.06835774
KEGG_LINOLEIC_ACID_METABOLISM	-1.495403	0.03962704	0.25468117
KEGG_MAPK_SIGNALING_PATHWAY	-1.3843179	<0.001	0.3835811
KEGG_NEUROACTIVE_LIGAND_RECEPTOR_INTERACTION	-1.9039419	<0.001	0.05041233
KEGG_RETINOL_METABOLISM	-1.4700123	0.03432494	0.27182007
KEGG_TYPE_I_DIABETES_MELLITUS	-1.681616	0.01179245	0.13151285

Suplementary Table S2. Common pathways among alteast 3 studies

Common among	Numbe	Pathway names	Experiments								Similar Pathway
E_GEOD_number			E_GEOD_38836 (space)		E_GEOD_57418 (simulate		E_GEOD_4209 (simulat		E_GEOD_43582 (space)		published with
		**Oncogenic Signature**	p value	FDR q value	p value	FDR q value	p value	FDR q valu	p value	FDR q valu	source data set
		**Upregulated Pathways**									
		KRAS.300_UP.V1_UP	0.036	0.028	0.045	0.102	<0.001	<0.001			This study only
38836, 57418, 4209	2	KRAS.600_UP.V1_UP	0.041	0.429	0.005	0.069	<0.001	<0.001			This study only
		CAHOY_ASTROCYTIC	0.019	0.171			0.003	0.006	0.039	0.064	This study only
		**Downregulated Pathways**									
	1	IL15_UP.V1_UP	<0.001	0.028			0.009	0.031	<0.001	0.01	This study only
		IL2_UP.V1_UP	<0.001	0.005			0.007	0.027	<0.001	0.01	This study only
		**Canonical Pathway**									
		**Upregulated Pathways**									
38836, 4209, 43582	3	REACTOME_OLFACTORY_SIGNALING_PATHWAY	<0.001	0.171			<0.001	0.00389	0.036697	0.43687	This study only
		KEGG_OLFACTORY_TRANSDUCTION	<0.001	0.269			<0.001	0.00376	0.009943	0.30722	This study only
		REACTOME_SLC_MEDIATED_TRANSMEMBRANE_TRANSPORT	0.008	1			0.002123	0.05971	0.036697	0.43687	**E_GEOD_57418**
43582, 4209, 57418	2	REACTOME_MEIOSIS			<0.001	<0.001	0.011928	0.16178	0.048533	0.951	This study only
		REACTOME_MEIOTIC_RECOMBINATION			<0.001	<0.001	<0.001	0.02332	0.003478	0.09935	**E_GEOD_4209**
		**Downregulated Pathways**									
38836, 4209, 43582, 57418	7	REACTOME_GENERATION_OF_SECOND_MESSENGER_MOLECUI	0.029411765	0.107111506	0.0037453	0.1277085	<0.001	<0.001	0.028761	0.16804	**E_GEOD_57418**
		KEGG_ALLOGRAFT_REJECTION	<0.001	0.029689314	<0.001	0.0031031	<0.001	<0.001	<0.001	0.05073	This study only
		KEGG_AUTOIMMUNE_THYROID_DISEASE	0.024539877	0.22512302	<0.001	0.0070073	<0.001	0.00223	<0.001	0.06386	This study only
		KEGG_ASTHMA	0.04845815	0.18180504	<0.001	<0.001	<0.001	<0.001	0.002262	0.04989	This study only
		KEGG_HEMATOPOIETIC_CELL_LINEAGE	0.03787879	0.355202	<0.001	0.1062244	0.00211	0.04456	0.002604	0.14569	This study only
		KEGG_TYPE_I_DIABETES_MELLITUS	0.005181347	0.06740264	<0.001	0.0083587	<0.001	<0.001	0.011792	0.13151	This study only
		KEGG_GRAFT_VERSUS_HOST_DISEASE	<0.001	0.037636507	0.0018083	0.0178608	<0.001	<0.001	0.046083	0.32126	This study only
4209, 43582, 57418	1	KEGG_INTESTINAL_IMMUNE_NETWORK_FOR_IGA_PRODUCTION			<0.001	<0.001	<0.001	0.0023	0.00232	0.06836	This study only
4209, 43582, 38836	5	PID_NFAT_TFPATHWAY	<0.001	<0.001			<0.001	<0.001	0.027149	0.25805	This study only
		BIOCARTA_CYTOKINE_PATHWAY	<0.001	0.003			0.006479	0.00323	0.008949	0.1551	**E_GEOD_38836**
		BIOCARTA_INFLAM_PATHWAY	<0.001	0.013			0.002123	0.00164	0.010776	0.10448	**E_GEOD_4209**
		KEGG_CYTOKINE_CYTOKINE_RECEPTOR_INTERACTION	<0.001	0.356			<0.001	0.03049	<0.001	0.06324	This study only
		BIOCARTA_NKT_PATHWAY	<0.001	0.042			<0.001	<0.001	0.002208	0.10063	This study only
4209, 38836, 57418	4	REACTOME_RESPIRATORY_ELECTRON_TRANSPORT_ATP_SYNTH	<0.001	0.114	<0.001	0.0093042	<0.001	<0.001			This study only
		REACTOME_TCA_CYCLE_AND_RESPIRATORY_ELECTRON_TRANS	<0.001	0.127	<0.001	0.0330239	<0.001	<0.001			This study only
		KEGG_LEISHMANIA_INFECTION	0.00729927	0.21167646	<0.001	0.0185478	<0.001	0.00378			This study only
		REACTOME_PD1_SIGNALING	0.003484321	0.026380578	<0.001	0.0047263	0.002169	0.00205			**E_GEOD_57418**
		**Cancer modules**									
		**Upregulated Pathways**									
38836, 57418 , 4209	1	MODULE_47	<0.001	0.36220282	0.0288714	0.2569136	<0.001	0.05232			This study only
		**Downregulated Pathways**									
4209, 43582, 57418, 38836	2	MODULE_75	<0.001	0.3264356	0.0183206	0.2728171	0.014	0.16536	0	0.08074	This study only
		MODULE_46	<0.001	0.36869243	0.0110236	0.2379103	0.003876	0.09939	0	0.07051	This study only
38836, 43582, 57418	1	MODULE_6	<0.001	0.59472054	0.0029718	0.1919518			0	0.0364	This study only
4209, 38836, 43582	1	MODULE_123	<0.001	0.3386426			0.00381	0.08929	0.018182	0.28747	This study only
4209, 57418, 38836	3	MODULE_114	<0.001	0.089578636	<0.001	0.1394381	<0.001	0.13944			This study only
		MODULE_151	<0.001	0.07395996	0.0030441	0.1389451	0.003044	0.13895			This study only
		MODULE_22	0.024242423	0.23292293	0.0069686	0.0378852	0.006969	0.03789			This study only
		**Chemical and Genetic perturbation**									
		**Upregulated Pathways**									
4209, 43582, 57418, 38836	1	RICKMAN_HEAD_AND_NECK_CANCER_C	0.018478261	0.75015676	<0.001	0.0170042	<0.001	0.03	0.038526	0.19122	This study only
38836, 57418 , 4209	4	DOANE_BREAST_CANCER_ESR1_DN	0.001908397	0.006619871	0.0085288	0.064161	0.001908	0.00662			This study only
		BOSCO_EPITHELIAL_DIFFERENTIATION_MODULE	<0.001	0.002441023	0.0159453	0.1027523	<0.001	0.00244			This study only
		SENGUPTA_NASOPHARYNGEAL_CARCINOMA_WITH_LMP1_DN	<0.001	0.06067855	0.0023753	0.1112074	<0.001	0.06068			This study only
		ROLEF_GLIS3_TARGETS	0.025096525	0.10299574	0.0275862	0.1421221	0.025097	0.103			This study only
		**Downregulated Pathways**									
38836, 43582, 4209	23	LU_EZH2_TARGETS_UP	<0.001	0.010796257			<0.001	0.0108	0.015625	0.34704	This study only
		ALTEMEIER_RESPONSE_TO_LPS_WITH_MECHANICAL_VENTILAT	<0.001	0.008097289			<0.001	0.0081	0.00266	0.13733	This study only
		MARTENS_BOUND_BY_PML_RARA_FUSION	<0.001	0.076636165			<0.001	0.07664	<0.001	0.30797	This study only
		GALINDO_IMMUNE_RESPONSE_TO_ENTEROTOXIN	0.016427105	0.07373151			0.016427	0.07373	0.002695	0.08093	This study only
		KUROZUMI_RESPONSE_TO_ONCOCYTIC_VIRUS	<0.001	0.001960704			<0.001	0.00196	0.018433	0.17785	This study only
		VILIMAS_NOTCH1_TARGETS_UP	<0.001	<0.001			<0.001	<0.001	0.011521	0.12424	This study only
		DAIRKEE_TERT_TARGETS_UP	0.007677543	0.11923367			0.007678	0.11923	0.006711	0.31035	This study only
		ZHAN_V1_LATE_DIFFERENTIATION_GENES_DN	0.026258206	0.023380904			0.026258	0.02338	0.046185	0.15197	This study only
		LI_DCP2_BOUND_MRNA	<0.001	<0.001			<0.001	<0.001	0.042714	0.26856	This study only
		QI_PLASMACYTOMA_UP	<0.001	<0.001			<0.001	<0.001	0.012308	0.29098	This study only
		GESERICK_TERT_TARGETS_DN	0.006622517	0.017800089			0.006623	0.0178	<0.001	0.01704	This study only
		AMIT_SERUM_RESPONSE_120_MCF10A	0.008032128	0.047357976			0.008032	0.04736	0.004854	0.13041	This study only
		SMIRNOV_CIRCULATING_ENDOTHELIOCYTES_IN_CANCER_UP	0.017509727	0.118825994			0.01751	0.11883	0.011696	0.20567	This study only
		KIM_ALL_DISORDERS_DURATION_CORR_DN	0.002040816	0.026470037			0.002041	0.02647	<0.001	0.13471	This study only
		PHONG_TNF_TARGETS_UP	0.004149378	0.016296463			0.004149	0.0163	<0.001	<0.001	This study only
		BROCKE_APOPTOSIS_REVERSED_BY_IL6	0.008048289	0.060231566			0.008048	0.06023	0.022161	0.26465	**E_GEOD_38836**
											**E-GEOD-43582**
											**EGEOD_4209**
		BASSO_CD40_SIGNALING_UP	<0.001	<0.001			<0.001	<0.001	0.007772	0.12497	This study only
		TIAN_TNF_SIGNALING_NOT_VIA_NFKB	0.014767933	0.023179647			0.014768	0.02318	0.013072	0.05105	This study only
		SEKI_INFLAMMATORY_RESPONSE_LPS_UP	<0.001	<0.001			<0.001	<0.001	0.016588	0.18593	This study only
		DIRMEIER_LMP1_RESPONSE_EARLY	<0.001	0.001008268			<0.001	0.00101	<0.001	<0.001	This study only

		AMIT_DELAYED_EARLY_GENES	0.023809524	0.05582707			0.02381	0.05583	<0.001	0.02215	This study only
		OSWALD_HEMATOPOIETIC_STEM_CELL_IN_COLLAGEN_GEL_UP	0.036259543	0.16577902			0.03626	0.16578	0.020134	0.32007	This study only
		KOBAYASHI_EGFR_SIGNALING_6HR_DN	0.02844639	0.036916964			0.028446	0.03692	0.021368	0.08795	This study only
38836, 57418, 4209	14	RODWELL_AGING_KIDNEY_UP	0.001872659	0.10017705	<0.001	0.1263286	0.001873	0.10018			This study only
		STARK_PREFRONTAL_CORTEX_22Q11_DELETION_DN	<0.001	0.001144373	0.002907	0.176888	<0.001	0.00114			This study only
		MOOTHA_VOXPHOS	<0.001	<0.001	<0.001	0.0248837	<0.001	<0.001			This study only
		YAO_TEMPORAL_RESPONSE_TO_PROGESTERONE_CLUSTER_17	<0.001	<0.001	0.0048077	0.1929142	<0.001	<0.001			This study only
		GOLDRATH_ANTIGEN_RESPONSE	<0.001	<0.001	<0.001	0.1638586	<0.001	<0.001			This study only
		CHUNG_BLISTER_CYTOTOXICITY_UP	<0.001	<0.001	0.0186625	0.2115011	<0.001	<0.001			This study only
		MORI_MATURE_B_LYMPHOCYTE_UP	<0.001	0.001879968	<0.001	0.0408339	<0.001	0.00188			This study only
		HSIAO_HOUSEKEEPING_GENES	<0.001	0.050137505	0.0344828	0.3536008	<0.001	0.05014			This study only
		WONG_MITOCHONDRIA_GENE_MODULE	<0.001	<0.001	0.0015873	0.1088735	<0.001	<0.001			This study only
		YAO_TEMPORAL_RESPONSE_TO_PROGESTERONE_CLUSTER_13	<0.001	<0.001	0.0313972	0.2641092	<0.001	<0.001			This study only
		GAVIN_FOXP3_TARGETS_CLUSTER_P4	0.004065041	0.023792034	0.0280992	0.2239213	0.004065	0.02379			This study only
		BECKER_TAMOXIFEN_RESISTANCE_UP	0.022403259	0.06684875	0.0178253	0.1337748	0.022403	0.06685			This study only
		WIELAND_UP_BY_HBV_INFECTION	<0.001	<0.001	0.0017007	0.0381877	<0.001	<0.001			This study only
		RUTELLA_RESPONSE_TO_HGF_DN	<0.001	0.006484654	0.0076923	0.2056434	<0.001	0.00648			This study only
		Cancer Gene neighbourhood									
		**Downregulated Pathways**									
38836, 43582, 4209	1	GNF2_IL2RB	0.005405406	0.05146252			<0.001	<0.001	0.03125	0.1091	This study only
		**Immunologic Signature**									
		**Downregulated Pathways**									
57418, 43582, 38836	1	GSE9988_LPS_VS_LPS_AND_ANTI_TREM1_MONOCYTE_DN	<0.001	0.011227942	<0.001	<0.001			0.034483	0.23749	This study only
38836, 43582,4209	12	GSE9006_HEALTHY_VS_TYPE_2_DIABETES_PBMC_AT_DX_UP	<0.001	0.014057427			<0.001	<0.001	<0.001	0.04869	This study only
		GSE9988_LPS_VS_VEHICLE_TREATED_MONOCYTE_UP	<0.001	<0.001			0.002041	<0.001	0.002778	0.04859	This study only
		GSE2706_UNSTIM_VS_2H_LPS_DC_DN	<0.001	<0.001			<0.001	<0.001	0.003155	0.01189	This study only
		GSE9988_LOW_LPS_VS_VEHICLE_TREATED_MONOCYTE_UP	<0.001	<0.001			<0.001	<0.001	<0.001	0.05659	This study only
		GSE37416_CTRL_VS_12H_F_TULARENSIS_LVS_NEUTROPHIL_DN	<0.001	0.00401273			<0.001	0.00332	<0.001	0.0048	This study only
		GSE9988_LOW_LPS_VS_CTRL_TREATED_MONOCYTE_UP	<0.001	<0.001			<0.001	<0.001	0.014493	0.08334	This study only
		GSE2706_UNSTIM_VS_2H_R848_DC_DN	<0.001	<0.001			<0.001	<0.001	0.011204	0.083	This study only
		GSE14769_UNSTIM_VS_40MIN_LPS_BMDM_DN	<0.001	<0.001			0.012474	0.02814	<0.001	0.04781	This study only
		GSE9988_LPS_VS_CTRL_TREATED_MONOCYTE_UP	<0.001	<0.001			<0.001	<0.001	0.008451	0.06786	This study only
		GSE22886_CD4_TCELL_VS_BCELL_NAIVE_UP	<0.001	<0.001			0.001845	0.00119	0.013587	0.09954	This study only
		GSE2706_UNSTIM_VS_2H_LPS_AND_R848_DC_DN	<0.001	<0.001			<0.001	<0.001	<0.001	0.04859	This study only
		GSE9988_ANTI_TREM1_VS_CTRL_TREATED_MONOCYTES_UP	<0.001	<0.001			0.011858	0.0202	0.020833	0.11054	This study only
38836, 57418, 4209	9	GSE22886_NEUTROPHIL_VS_MONOCYTE_DN	<0.001	0.001893921	<0.001	<0.001	<0.001	<0.001			This study only
		GSE360_L_DONOVANI_VS_B_MALAYI_HIGH_DOSE_MAC_DN	<0.001	0.053301018	<0.001	<0.001	<0.001	<0.001			This study only
		GSE17721_0.5H_VS_4H_CPG_BMDM_UP	0.03125	0.14473116	<0.001	<0.001	<0.001	<0.001			This study only
		GSE28237_FOLLICULAR_VS_LATE_GC_BCELL_DN	<0.001	0.059723753	0.024961	0.1022976	<0.001	<0.001			This study only
		GSE29618_BCELL_VS_MDC_DN	<0.001	0.023126367	<0.001	<0.001	<0.001	<0.001			This study only
		GSE29618_PDC_VS_MDC_DAY7_FLU_VACCINE_DN	<0.001	0.020071646	<0.001	<0.001	0.001996	0.00837			This study only
		GSE17580_TREG_VS_TEFF_S_MANSONI_INF_UP	0.027777778	0.05840059	0.0427632	0.1381722	<0.001	<0.001			This study only
		GSE1460_DP_THYMOCYTE_VS_NAIVE_CD4_TCELL_ADULT_BLO	<0.001	0.00340889	0.0446571	0.1619372	<0.001	<0.001			This study only
		GSE11057_NAIVE_CD4_VS_PBMC_CD4_TCELL_DN	0.027027028	0.0479284	<0.001	<0.001	<0.001	0.00157			This study only
		**Hallmark gene set**									
		**Downregulated Pathways**									
4209, 38836, 43582	1	HALLMARK_TNFA_SIGNALLING_VIA_NFKB	<0.001	0.011152694			<0.001	<0.001	<0.001	<0.001	**E_GEOD_38836**
4209, 38836, 57418	1	HALLMARK_OXIDATIVE_PHOSPHORYLATION	0.003282276	0.07486968	0.0117878	0.5916052	<0.001	<0.001			**E-GEOD-43582**
		**Motif gene sets**									
		None									
		**Positional gene sets**									
		None									

Supplementary Table S3. Short description of common gene sets

(Details of all those gene sets can be found in MSigDB http://software.broadinstitute.org/gsea/index.jsp)

Gene Set	Description
**Oncogenic Signature**	
KRAS.300_UP.V1_UP	Genes up-regulated in four lineages of epithelial cell lines over-expressing an oncogenic form of KRAS [Gene ID=3845] gene.
KRAS.600_UP.V1_UP	Genes up-regulated in four lineages of epithelial cell lines over-expressing an oncogenic form of KRAS [Gene ID=3845] gene.
CAHOY_ASTROCYTIC	Genes up-regulated in astrocytes.
IL15_UP.V1_UP	Genes up-regulated in Sez-4 cells (T lymphocyte) that were first starved of IL2 [Gene ID=3558] and then stimulated with IL15 [Gene ID=3600].
IL2_UP.V1_UP	Genes up-regulated in Sez-4 cells (T lymphocyte) that were first starved of IL2 [Gene ID=3558] and then stimulated with IL2 [Gene ID=3558].
**Canonical Pathway**	
REACTOME_OLFACTORY_SIGNALING_PATHWAY	Genes involved in Olfactory Signaling Pathway
KEGG_OLFACTORY_TRANSDUCTION	Olfactory transduction
REACTOME_SLC_MEDIATED_TRANSMEMBRANE_TRANSPORT	Genes involved in SLC-mediated transmembrane transport
REACTOME_MEIOSIS	Genes involved in Meiosis
REACTOME_MEIOTIC_RECOMBINATION	Genes involved in Meiotic Recombination
REACTOME_GENERATION_OF_SECOND_MESSENGER_MOLECULES	Genes involved in Generation of second messenger molecules
KEGG_ALLOGRAFT_REJECTION	Allograft rejection
KEGG_AUTOIMMUNE_THYROID_DISEASE	Autoimmune thyroid disease
KEGG_ASTHMA	Asthma

KEGG_HEMATOPOIETIC_CELL_LINEAGE	Hematopoietic cell lineage
KEGG_TYPE_I_DIABETES_MELLITUS	Type I diabetes mellitus
KEGG_GRAFT_VERSUS_HOST_DISEASE	Graft-versus-host disease
KEGG_INTESTINAL_IMMUNE_NETWORK_FOR_IGA_PRODUCTION	Intestinal immune network for IgA production
PID_NFAT_TFPATHWAY	Calcineurin-regulated NFAT-dependent transcription in lymphocytes
BIOCARTA_CYTOKINE_PATHWAY	Cytokine Network
BIOCARTA_INFLAM_PATHWAY	Cytokines and Inflammatory Response
KEGG_CYTOKINE_CYTOKINE_RECEPTOR_INTERACTION	Cytokine-cytokine receptor interaction
BIOCARTA_NKT_PATHWAY	Selective expression of chemokine receptors during T-cell polarization
REACTOME_RESPIRATORY_ELECTRON_TRANSPORT_ATP_SYNTHESIS_BY_CHEMIOSMOTIC_COUPLING_AND_HEAT_PRODUCTION_BY_UNCOUPLING_PROTEINS_	Genes involved in Respiratory electron transport, ATP synthesis by chemiosmotic coupling, and heat production by uncoupling proteins.
REACTOME_TCA_CYCLE_AND_RESPIRATORY_ELECTRON_TRANSPORT	Genes involved in The citric acid (TCA) cycle and respiratory electron transport
KEGG_LEISHMANIA_INFECTION	Leishmania infection
REACTOME_PD1_SIGNALING	Genes involved in PD-1 signaling
**Cancer modules**	
MODULE_47	Genes in the cancer module 47
MODULE_75	Genes in the cancer module 75

MODULE_46	Genes in the cancer module 75
MODULE_6	Genes in the cancer module 6
MODULE_123	Genes in the cancer module 123
MODULE_114	Genes in the cancer module 114
MODULE_151	Genes in the cancer module 151
MODULE_22	Genes in the cancer module 22
**Chemical and Genetic perturbation**	
RICKMAN_HEAD_AND_NECK_CANCER_C	Cluster c: genes identifying an intrinsic group in head and neck squamous cell carcinoma (HNSCC).
DOANE_BREAST_CANCER_ESR1_DN	Genes down-regulated in breast cancer samples positive for ESR1 [GeneID=2099] compared to the ESR1 negative tumors.
BOSCO_EPITHELIAL_DIFFERENTIATION_MODULE	Genes representing epithelial differentiation module in sputum during asthma exacerbations.
SENGUPTA_NASOPHARYNGEAL_CARCINOMA_WITH_LMP1_DN	Genes down-regulated in nasopharyngeal carcinoma (NPC) positive for LMP1 [GeneID=9260], a latent gene of Epstein-Barr virus (EBV).
ROLEF_GLIS3_TARGETS	Genes down-regulated in nasopharyngeal carcinoma (NPC) positive for LMP1 [GeneID=9260], a latent gene of Epstein-Barr virus (EBV).
LU_EZH2_TARGETS_UP	Genes up-regulated in SKOV3ip1 cells (ovarian cancer) upon knockdown of EZH2 [GeneID=2146] by RNAi.
ALTEMEIER_RESPONSE_TO_LPS_WITH_MECHANICAL_VENTILATION	Genes up-regulated in lung tissue upon LPS aspiration with mechanical ventilation (MV) compared to control (PBS aspiration without MV).
MARTENS_BOUND_BY_PML_RARA_FUSION	Genes with promoters occupied by PML-RARA fusion [GeneID=5371,5914] protein in acute promyelocytic leukemia(APL) cells NB4 and two APL primary blasts, based on Chip-seq data.
GALINDO_IMMUNE_RESPONSE_TO_ENTEROTOXIN	Genes up-regulated in macrophages by aerolysin-related cytotoxic enterotoxin (Act) from Aeromonas hydrophila.

KUROZUMI_RESPONSE_TO_ONCOCYTIC_VIRUS	Inflammatory cytokines and their receptors modulated in brain tumors after treatment with an oncocytic virus, a potential anticancer therapy.
VILIMAS_NOTCH1_TARGETS_UP	Genes up-regulated in bone marrow progenitors by constitutively active NOTCH1 [GeneID=4851].
DAIRKEE_TERT_TARGETS_UP	Genes up-regulated in non-spontaneously immortalizing (NSI) primary breast cancer tumor cultures upon expression of TERT [GeneID=7015] off a retroviral vector.
ZHAN_V1_LATE_DIFFERENTIATION_GENES_DN	The v1LDG down-regulated set: most variable late differentiation genes (LDG) with similar expression patterns in tonsil plasma cells (TPC) and multiple myeloma (MM) samples.
LI_DCP2_BOUND_MRNA	Genes encoding mRNA transcripts specifically bound by DCP2 [GeneID=167227].
QI_PLASMACYTOMA_UP	Up-regulated genes that best disciminate plasmablastic plasmacytoma from plasmacytic plasmacytoma tumors.
GESERICK_TERT_TARGETS_DN	Genes down-regulated in MEF cells (embryonic fibroblasts) with TERT [GeneID=7015] knockout, after expression of the gene off a retroviral vector.
AMIT_SERUM_RESPONSE_120_MCF10A	Genes whose expression peaked at 120 min after stimulation of MCF10A cells with serum.
SMIRNOV_CIRCULATING_ENDOTHELIOCYTES_IN_CANCER_UP	Genes up-regulated in circulating endothelial cells (CEC) from cancer patients compared to those from healthy donors.
KIM_ALL_DISORDERS_DURATION_CORR_DN	Genes whose expression in brain significantly and negatively correlated with the duration of all psychiatric disorders studied.
PHONG_TNF_TARGETS_UP	Genes up-regulated in Calu-6 cells (lung cancer) at 1 h time point after TNF [GeneID=7124] treatment.
BROCKE_APOPTOSIS_REVERSED_BY_IL6	Genes changed in INA-6 cells (multiple myeloma, MM) by re-addition of IL6 [GeneID=3569] after its initial withdrawal for 12h.
BASSO_CD40_SIGNALING_UP	Gene up-regulated by CD40 [GeneID=958] signaling in Ramos cells (EBV negative Burkitt lymphoma).
TIAN_TNF_SIGNALING_NOT_VIA_NFKB	Genes modulated in HeLa cells (cervical carcinoma) by TNF [GeneID=7124] not via NFKB pathway.
SEKI_INFLAMMATORY_RESPONSE_LPS_UP	Genes up-regulated in hepatic stellar cells after stimulation with bacterial lipopolysacharide (LPS).
DIRMEIER_LMP1_RESPONSE_EARLY	Clusters 1 and 2: genes up-regulated in B2264-19/3 cells (primary B lymphocytes) within 30-60 min after activation of LMP1 (an oncogene encoded by Epstein-Barr virus, EBV).
AMIT_DELAYED_EARLY_GENES	Delayed early genes (DEG) which are coordinately down-regulated in multiple epithelial tumor types.

OSWALD_HEMATOPOIETIC_STEM_CELL_IN_COLLAGEN_GEL_UP	Genes up-regulated in hematopoietic stem cells (HSC, CD34+ [GeneID=947]) cultured in a three-dimentional collagen gel compared to the cells grown in suspension.
KOBAYASHI_EGFR_SIGNALING_6HR_DN	Genes down-regulated in H1975 cells (non-small cell lung cancer, NSCLC) resistant to gefitinib [PubChem=123631] after treatment with EGFR inhibitor CL-387785 [PubChem=2776] for 6h.
RODWELL_AGING_KIDNEY_UP	Genes whose expression increases with age in normal kidney.
STARK_PREFRONTAL_CORTEX_22Q11_DELETION_DN	Genes down-regulated in prefrontal cortex (PFC) of mice carrying a hemizygotic microdeletion in the 22q11.2 region.
MOOTHA_VOXPHOS	Genes involved in oxidative phosphorylation; based on literature and sequence annotation resources and converted to Affymetrix HG-U133A probe sets.
YAO_TEMPORAL_RESPONSE_TO_PROGESTERONE_CLUSTER_17	Genes co-regulated in uterus during a time course response to progesterone [PubChem=5994]: SOM cluster 17.
GOLDRATH_ANTIGEN_RESPONSE	Genes up-regulated at the peak of an antigen response of naive CD8+ [GeneID=925;926] T-cells.
CHUNG_BLISTER_CYTOTOXICITY_UP	Genes up-regulated in blister cells from patients with adverse drug reactions (ADR).
MORI_MATURE_B_LYMPHOCYTE_UP	Up-regulated genes in the B lymphocyte developmental signature, based on expression profiling of lymphomas from the Emu-myc transgenic mice: the mature B
HSIAO_HOUSEKEEPING_GENES	Housekeeping genes identified as expressed across 19 normal tissues.
WONG_MITOCHONDRIA_GENE_MODULE	Genes that comprise the mitochondria gene module
YAO_TEMPORAL_RESPONSE_TO_PROGESTERONE_CLUSTER_13	Genes co-regulated in uterus during a time course response to progesterone [PubChem=5994]: SOM cluster 13.
GAVIN_FOXP3_TARGETS_CLUSTER_P4	Cluster P4 of genes with similar expression profiles in peripheral T lymphocytes after FOXP3 [GeneID=50943] loss of function (LOF).
BECKER_TAMOXIFEN_RESISTANCE_UP	Genes up-regulated in a breast cancer cell line resistant to tamoxifen [PubChem=5376] compared to the parental line sensitive to the drug.
WIELAND_UP_BY_HBV_INFECTION	Genes induced in the liver during hepatitis B (HBV) viral clearance in chimpanzees.

RUTELLA_RESPONSE_TO_HGF_DN	Genes down-regulated in peripheral blood monocytes by HGF [GeneID=3082]
**Cancer Gene neighbourhood**	
GNF2_IL2RB	Neighborhood of IL2RB
GSE9988_LPS_VS_LPS_AND_ANTI_TREM1_MONOCYTE_DN	Genes down-regulated in comparison of monocytes treated with 5000 ng/ml LPS (TLR4 agonist) versus monocytes treated with anti-TREM1 [GeneID=54210].
GSE9006_HEALTHY_VS_TYPE_2_DIABETES_PBMC_AT_DX_UP	Genes up-regulated in comparison of peripheral blood mononuclear cells (PBMC) from healthy donors versus PBMCs from patients with type 2 diabetes at the time of diagnosis.
GSE9988_LPS_VS_VEHICLE_TREATED_MONOCYTE_UP	Genes up-regulated in comparison of monocytes treated with 1 ng/ml LPS (TLR4 agonist) versus monocytes treated with vehicle.
GSE2706_UNSTIM_VS_2H_LPS_DC_DN	Genes down-regulated in comparison of unstimulated dendritic cells (DC) at 0 h versus DCs stimulated with LPS (TLR4 agonist) for 2 h.
GSE9988_LOW_LPS_VS_VEHICLE_TREATED_MONOCYTE_UP	Genes up-regulated in comparison of monocytes treated with 1 ng/ml LPS (TLR4 agonist) versus monocytes treated with control IgG.
GSE37416_CTRL_VS_12H_F_TULARENSIS_LVS_NEUTROPHIL_DN	Genes down-regulated in comparison of control polymorphonuclear leukocytes (PMN) at 12 h versus PMN treated with F. tularensis vaccine at 12 h.
GSE9988_LOW_LPS_VS_CTRL_TREATED_MONOCYTE_UP	Genes up-regulated in comparison of monocytes treated with 1 ng/ml LPS (TLR4 agonist) versus monocytes treated with control IgG.
GSE2706_UNSTIM_VS_2H_R848_DC_DN	Genes down-regulated in comparison of unstimulated dendritic cells (DC) at 0 h versus DCs stimulated with R848 for 2 h.
GSE14769_UNSTIM_VS_40MIN_LPS_BMDM_DN	Genes down-regulated in comparison of unstimulated macrophage cells versus macrophage cells stimulated with LPS (TLR4 agonist) for 40 min.
GSE9988_LPS_VS_CTRL_TREATED_MONOCYTE_UP	Genes up-regulated in comparison of monocytes treated with 5000 ng/ml LPS (TLR4 agonist) versus monocytes treated with control IgG.
GSE22886_CD4_TCELL_VS_BCELL_NAIVE_UP	Genes up-regulated in comparison of naive CD4 [GeneID=920] T cells versus naive B cells.
GSE2706_UNSTIM_VS_2H_LPS_AND_R848_DC_DN	Genes down-regulated in comparison of unstimulated dendritic cells (DC) at 0 h versus DCs stimulated with LPS (TLR4 agonist) and R848 for 2 h.
GSE9988_ANTI_TREM1_VS_CTRL_TREATED_MO	Genes up-regulated in comparison of monocytes treated with anti-TREM1

NOCYTES_UP	[GeneID=54210] versus monocytes treated with control IgG.
GSE22886_NEUTROPHIL_VS_MONOCYTE_DN	Genes down-regulated in comparison of neutrophils versusl monocytes.
GSE360_L_DONOVANI_VS_B_MALAYI_HIGH_DOSE_MAC_DN	Genes down-regulated in comparison of macrophages exposed to L. donovani versus macrophages exposed to 50 worms/well B. malayi.
GSE17721_0.5H_VS_4H_CPG_BMDM_UP	Genes up-regulated in comparison of dendritic cells (DC) stimulated with CpG DNA (TLR9 agonist) at 0.5 h versus those stimulated with CpG DNA (TLR9 agonist) at 4 h.
GSE28237_FOLLICULAR_VS_LATE_GC_BCELL_DN	Genes down-regulated in comparison of follicular B cells versus late germinal center (GC) B cells.
GSE29618_BCELL_VS_MDC_DN	Genes down-regulated in comparison of B cells versus myeloid dendritic cells (mDC).
GSE29618_PDC_VS_MDC_DAY7_FLU_VACCINE_DN	Genes down-regulated in comparison of plasmacytoid dendritic cells (DC) from influenza vaccinee at day 7 post-vaccination versus myeloid DCs at day 7 post-vaccination.
GSE17580_TREG_VS_TEFF_S_MANSONI_INF_UP	Genes up-regulated in comparison of regulatory T cell (Treg) from mice infected with S. mansoni versus T effector cells from the infected mice.
GSE1460_DP_THYMOCYTE_VS_NAIVE_CD4_TCELL_ADULT_BLOOD_UP	Genes up-regulated in comparison of CD4 [GeneID=920] CD8 thymocytes versus naive CD4 [GeneID=920] T cells from adult blood.
GSE11057_NAIVE_CD4_VS_PBMC_CD4_TCELL_DN	Genes down-regulated in comparison of naive T cells versus peripheral blood mononuclear cells (PBMC).
**Hallmark gene set**	
HALLMARK_TNFA_SIGNALLING_VIA_NFKB	Genes regulated by NF-kB in response to TNF [GeneID=7124].
HALLMARK_OXIDATIVE_PHOSPHORYLATION	Genes encoding proteins involved in oxidative phosphorylation.
**Motif gene sets**	
**Positional gene sets**	

Supplementary Table S4. Distribution of leading edge genes in NMFC clusters

E-GEOD-38836 (Upregulated)		E-GEOD-43582 (Upregulated)		E-GEOD-43582 (downregulated)		E-GEOD-57418 (Upregulated)		E-GEOD-4209 (Upregulated)		E-GEOD-4209 (Downregulated)	
Cluster	Member genes	Cluster	Member genes	Cluster	Member genes	Cluster	Member genes	Cluster	Member genes	Cluster	Member genes
1	DHRS9	1	OR11H1	1	DUSP2	1	PTPRC	1	SLC4A4	1	CCNC
1	C19orf40	1	CDK2	1	TNNI3	1	GADD45A	1	APP	1	PRPF3
1	UGT2B17	1	SLC22A4	1	HCRT	1	TNFSF4	1	ITPR2	1	ADAR
1	OPN3	1	KIF2A	1	VEGFB	1	RNPS1	1	LYZ	1	U2AF1
1	VEGFA	1	PDE3A	1	TNFRSF14	1	EXOC2	1	SPTBN1	1	INPP5D
1	TAAR9	1	ZNF610	1	GRM7	1	IRS1	1	ALDH5A1	1	RABGGTA
1	PIPOX	1	NUP153	1	MAPK8IP3	1	PSMB2	1	CCR9	1	ICAM1
1	ADRA2B	1	OR2H1	1	MADCAM1	1	PSMA5	1	ABLIM1	1	UBE4A
1	CNDP1	1	AGPS	1	MAPT	1	SRSF10	1	LEF1	1	POLE
1	ABCD1	1	SLC12A6	1	SGCA	1	ZNF566	1	ACTN1	1	SEC24B
1	COL3A1	1	KLHL20	1	STX1A	1	POLR3A	1	COL6A2	1	RFC4
1	OR5L2	1	RAD54L	1	CXCL12	1	BMPR1A	1	HMOX1	1	CENPM
1	ATP1A2	1	TDP1	1	EGR4	1	ZNF596	1	HIST1H2AC	1	PFDN1
1	AKR1D1	1	CDC14B	1	NMB	1	GADD45B	1	HIST1H2BD	1	PIGA
1	CYSLTR2	1	ZNF559	1	RAMP1	1	KLC1	1	S100A9	1	PARP3
1	ADM	1	OIP5	1	CACNG8	1	TYK2	1	CLDN9	1	BCL2L1
1	ATP8A2	1	DLAT	1	MMP15	1	ADIPOR1	1	COL4A5	1	PELI1
1	SNCA	1	NARS	1	COX6A2	1	E2F4	1	MBL2	1	IDH3B
1	SULT1E1	1	SMC1B	1	GABRE	1	PSMD2	1	RXFP3	1	TDRD7
1	SLC26A7	1	SLC35C1	1	CNR1	1	RAD9A	1	CXCL6	1	SRM
1	GABRB3	1	KIAA1598	1	PLA2G6	1	ZNF19	1	FOXN1	1	CYB5R3
1	PTK2B	1	LDHB	1	MUC6	1	IL7R	1	PLA2G6	1	PSMD7
1	CHRNE	1	FANCF	1	LPAR2	1	ZNF420	1	GLRB	1	ANAPC5
1	S1PR3	1	ZNF221	1	PROC	1	DVL3	1	PHKG1	1	CD81
1	ABAT	1	UGDH	1	PPP3R2	1	MAP4K4	1	OMD	1	PSMC2
1	KCNMB2	1	NEK2	1	PTPRN	1	TGS1	1	CLEC4E	1	RCAN2
1	FAN1	1	PTPN11	1	CD70	1	EIF2AK2	1	CRISPLD2	1	CCND3
1	ABCC9	1	DDB1	1	GP1BB	1	PHLPP1	1	SLC7A2	1	CS
1	AGTR1	1	PTPN21	1	GRIN2D	1	AKT1	1	MLN	1	ADD1
1	SLC30A2	1	AMACR	1	THPO	1	ABL1	1	CHRDL1	1	UBA3
1	ATP11B	1	ALDH3A2	1	CX3CL1	1	ZNF584	1	MUC4	1	FPGS
1	ATP2B2	1	GIT2	1	ATP1A2	1	CENPL	1	LAMC2	1	TUBB4B
1	GAD2	1	CASP2	1	GRIK4	1	NRBP1	1	MUC6	1	NT5C
1	KNG1	1	DOCK1	1	EPOR	1	CCNT2	1	PKP2	1	IRF1
1	CCKAR	1	MCM3	1	NR0B1	1	HMGCS1	1	SLC7A10	1	NDUFA9
1	SLC35D2	1	UBE2D1	1	ALOX5	1	CYP51A1	1	ADAM11	1	PTTG1
1	CAMK2B	1	E2F7	1	ARR3	1	RANGAP1	1	PARD3	1	RALA
1	SSTR1	1	OR2M4	1	KIR3DL1	1	CYP1A1	1	SLC8A2	1	POLD1
1	SLC28A2	1	CBX5	1	GRM4	1	ZNF189	1	OR5I1	1	PSMD14
1	AKR1C1	1	TRIP12	1	INHBB	1	HIF1AN	1	COL4A6	1	TOLLIP
1	AKR1C2	1	UBE2G2	1	TNFRSF18	1	PARP14	1	OR10C1	1	NME7
1	EPB41L1	1	GHR	1	PRKACG	1	ARAF	1	HIST1H2BJ	1	CASP8
1	SLC1A3	1	ADCY3	1	GUCY2D	1	BCL2L11	1	CFH	1	SF3B2
1	SYK	1	PSMD12	1	PRLHR	1	ZNF141	1	PVRL1	1	CDK9
1	CALCR	1	PRPF8	1	DNAJC5	1	ZNF230	1	NR1H3	1	NCAPD2
1	CHDH	1	AKT1	1	IL9	1	HNRNPA2B1	1	CSTA	1	NDUFV1
1	MAOB	1	OR6T1	1	PIK3CD	1	POLE	1	ANGPT2	1	TSG101
1	PLA2G5	1	GCH1	1	GNAO1	1	UNG	1	CCR8	1	CYC1
1	NPFF	1	PGM2	1	RAMP2	1	ZNF468	1	GH2	1	PPP2R5E
1	LAMB1	1	OR11H6	1	GRIK3	1	NUPL1	1	SCN2B	1	TRIP12
1	CTNNA1	1	HGSNAT	1	ELANE	1	ZNF614	1	CHRM3	1	RHOA
1	SYN1	1	TUBA1C	1	CCKBR	1	PSMB3	1	HIST1H2AE	1	SNRNP40
1	OR2L2	1	TAF6	1	DUSP4	1	SRSF9	1	UGT1A3	1	CD3D
1	COL2A1	1	AP1M1	1	NPFFR1	1	RASA1	1	ADAMTS5	1	SPCS1
1	GRIN3B	1	SMARCA2	1	TNNI1	1	ATF1	1	CHRM4	1	DHX16
1	HMMR	1	PDK2	1	BLK	1	PCBP1	1	PLXNB3	1	PMPCA
2	HNMT	1	SLC23A2	1	TACR2	1	MAGI2	1	SERPINB7	1	CCR10
2	SLC5A7	1	SLCO3A1	1	ADRA2C	1	CDK9	1	ITGA2	1	GPAA1
2	G6PC2	1	OR13C9	1	CHRNB2	1	ZNF140	1	COL13A1	1	PTP4A1
2	KCNA6	1	PIGG	1	KCNK17	1	MED17	1	COL9A3	1	TAF10
2	SLC4A3	1	ZNF773	1	TNFRSF4	1	RAC3	1	HCN4	1	GNAI3
2	GAST	1	PRPF4	1	ADRA2A	1	NUDC	1	MMP3	1	LDHA
2	SLC30A8	1	CKAP5	1	GABRB3	1	SLC30A5	1	SCUBE2	1	HARS
2	ADCYAP1	1	SNRNP200	1	GRIP2	1	EIF4G1	1	CLDN16	1	RPL23
2	PPYR1	1	DARS	1	CD80	1	RNMT	1	PLBD1	1	GCNT1
2	CTH	1	MAPRE1	1	KCNS2	1	CCT5	1	GABRR1	1	ID2
2	KCNJ5	1	NF2	1	IL13	1	CSTF3	1	ALDH2	1	ERCC1
2	GRIP2	1	RPL23	1	MUC16	1	ILK	1	CALML5	1	TUBA1C
2	HES1	1	MNAT1	1	ATP2A1	1	DCTN1	1	MET	1	CNOT1
2	FGB	1	CCT4	1	CYP2C8	1	EXOC4	1	KCND2	1	DDX23
2	SLC44A4	1	NUP214	1	TACR3	1	NTRK1	1	OR2H1	1	DHX15
2	KCNMB1	1	GAN	1	CYP2D6	1	AGRN	1	TGM4	1	OXA1L
2	ADCYAP1R1	1	MTR	1	P2RX3	1	CHUK	1	SLC22A2	1	CDK4
2	CDH24	1	EIF2S3	1	CCL4	1	DDX39B	1	PAK3	1	RAF1
2	GLRA3	1	LIN54	1	ANPEP	1	GTF2H3	1	RAB3B	1	RNGTT
2	SLC15A1	1	MTMR4	1	CCL25	2	PSMD6	1	SPTA1	1	WDR77
2	KCNJ9	1	PCM1	1	GRM3	2	HNRNPU	1	MMP28	1	CDC27
2	CLCA1	1	KIF2C	1	DGKA	2	POLR2D	1	PDE1C	1	POLR3B

2 PNOC	1 CLASP1	1 GABRR1	2 POLR1C	1 CYP7A1	1 RBBP7
2 SLC6A14	1 FCGR1B	1 MC5R	2 SQSTM1	1 GLI3	1 PSMC1
2 ADH7	1 PXMP4	1 SPN	2 PPIL1	1 KCNC4	1 PSME1
2 ITGA7	1 CSE1L	1 IFNA16	2 RBM8A	1 RASGRF1	1 TUBA1B
2 RDH16	1 DDX20	1 JUN	2 ERCC6L	1 PTCH2	1 ARIH2
2 OR2B6	1 MTMR3	1 CD247	2 DHX8	1 HIST1H3A	1 PARK7
2 PSAT1	1 TXNRD1	1 KCNG1	2 ZNF471	1 OPRM1	1 CTLA4
2 CNR2	1 ZNF614	1 COL6A2	2 RETNLB	1 SYT2	1 EIF2B1
2 FPR3	1 CENPA	1 P2RX2	2 PPARD	1 LHCGR	1 MAP2K4
2 SLC5A3	1 UBA6	1 MAPK11	2 SRF	1 GNG3	1 UBA2
2 SLC26A4	1 STT3A	1 WNT9A	2 CD70	1 LAMA4	1 ATIC
2 SLC6A7	1 CFLAR	1 CNTFR	2 PSENEN	1 CST6	1 TLR7
2 TNN	1 SEPHS1	1 GRIN3B	2 ZNF559	1 ITGA7	1 HLA.DPA1
2 NPB	1 CLCA4	1 GPR132	2 MALT1	1 RYR2	1 CYCS
2 RPS6KA6	1 OR10J5	1 GRIK5	2 ZNF473	1 CFB	1 MINA
2 CCR9	1 ITGA5	1 SYN3	2 FDFT1	1 CHRNB4	1 EIF3A
2 CACNB2	1 YARS2	1 F2RL3	2 HIST1H2BO	1 OPN1SW	1 LSM6
2 SLC3A2	1 ALG10B	1 CACNG4	2 PFN2	1 SLC7A11	1 GRB2
2 OR7E24	1 CCNG1	1 HRH3	2 HIST1H2BB	1 LAMA3	1 NCAPH
2 GNB3	1 PRKAR2B	1 MLNR	2 HIST1H2BE	1 KCNS1	1 TRIM37
2 AGTR2	1 UBA3	1 DEFB123	2 CX3CL1	1 CREB1	1 SEC23A
2 SLC5A5	1 PRIM1	1 LIF	2 IRF3	1 GUCY1A2	1 UBE2G2
2 DARC	1 SLC11A2	1 CTRB1	2 MCM8	1 C1S	1 ADAM10
2 CNGB1	1 CCNB3	1 DRD4	2 TRIM33	1 REN	1 MARCKSL1
2 SLC4A8	1 B2M	1 ADH7	2 ARIH1	1 LPAR3	1 PUF60
2 SLCO1C1	1 MAPK9	1 UTS2R	2 IL1RAP	1 SRPX2	1 ADSS
2 DAB2	1 PSMA6	1 CHAT	2 PPP3CB	1 PTH2R	1 SMC4
2 LPAR1	1 FGFR1OP	1 GALR3	2 SNRNP27	1 ADAMTS13	1 PPM1B
2 MIP	1 RPA2	1 GPR50	2 ZNF530	1 PI3	1 GNB2L1
2 LAMA1	1 SYNE1	1 SLC22A2	2 TRIM28	1 KCNK2	1 IL17A
2 NTSR2	1 USP1	1 CCR5	2 PRPF31	1 CCL1	1 ARPC5
2 GATM	1 CYCS	1 KLK2	2 CCDC99	1 TSHR	1 EDEM3
2 MYL9	1 DIDO1	1 ENO3	2 GRK5	1 UGDH	1 ELAVL1
2 CCL25	1 TRIM21	1 ZAP70	2 PRKCZ	1 IFNA8	1 MGAT5
2 GUCA1B	1 ZNF555	1 STX4	2 CDC23	1 VCAN	1 TDG
2 GHRH	1 HERC2	1 ADRBK1	2 SRSF11	1 OR3A3	1 TNFAIP3
2 INS	1 ZNF30	1 ALDH1A2	2 ZNF226	1 TRPC5	1 NRAS
2 GABRE	1 ARSB	1 KCNC3	2 PAG1	1 PPARA	1 PRKCQ
2 EGFR	1 NUP54	1 FGR	2 PDE3B	1 KCNB2	1 CD44
2 SLC9A7	1 GART	1 GGT5	2 CDC26	1 LIMK1	1 TIAM1
2 LAMB4	1 PSIP1	1 CCR2	2 SATB1	1 RGS4	1 IL6ST
2 ABCA12	1 TPR	1 FGF8	2 ZNF544	1 ADAM18	1 PRPF4
2 ACTN2	1 HDAC1	1 TRAF2	2 TUBA4A	1 GATA4	1 KARS
2 OR1A2	1 EIF2S2	1 CACNA1A	2 NR3C1	1 KDR	1 VARS
2 SST	1 NUP85	1 GALR2	2 LAT	1 MC4R	1 TAF4B
2 GABRB1	1 SNRPB	1 KCNC1	2 CCRL1	1 FRAS1	1 CDC40
2 PTAFR	1 HUWE1	1 TAS1R3	2 NUF2	1 SPAM1	1 SF3B1
2 PTH2	1 CDC14A	1 DRD5	2 UBE2D2	1 OR6A2	1 DLAT
2 CSF2RA	1 MTMR1	1 RELA	2 MED31	1 TNNT1	1 FCGR3B
2 FIGF	1 MCFD2	1 OPN1MW2	2 PRKAR1B	1 EFEMP1	1 KIF4A
2 THBS2	1 SLC35D2	1 ESRRA	2 ZNF337	1 NR0B1	1 TTC37
2 BCMO1	1 OR1L4	1 MKNK2	2 IKBKG	1 GLP1R	1 NCF2
2 ITGB5	1 OR52E6	1 ST6GALNAC4	2 WWOX	1 GRPR	1 PRKCD
2 DEFB129	1 MCL1	1 CACNA1H	2 CEP70	1 CA9	1 HADHA
2 JAM2	1 TXNL4A	1 HTR1B	2 ATF4	1 SEMA5A	1 SSRP1
2 ABCC4	1 AARS	1 TNNI2	2 CASP9	1 EMID1	1 IRS1
2 SLC17A6	1 ANAPC13	1 FOXO3	2 WNT11	1 PLCB4	1 PMEPA1
2 WNT6	1 IL13RA1	1 CNGB1	2 DHX16	1 HIST1H4A	1 HK1
2 UROC1	1 ASB13	1 MUC7	2 ZNF514	1 LIMS3	1 LZTS1
2 UGT2B4	1 CLIP1	1 SLC6A7	2 TNFRSF13B	1 RIMS1	1 ZNF143
2 SLC2A2	1 FUCA1	1 SLC6A2	2 ICOS	1 CTNNA2	1 GAPDH
2 SLC11A1	1 HNRNPA3	1 FGF19	2 PSMB4	1 NODAL	1 MKRN1
2 SERPING1	1 DTX3L	1 IL20	2 FOXO4	1 TRHR	1 VAV1
2 PTH	1 SLC6A9	1 CETP	2 TGIF2	1 KCNJ10	1 ARHGAP10
2 P2RX3	1 YWHAH	1 KNG1	2 AIM2	1 UGT2B17	1 CUX1
2 OR51I1	1 INPP5K	1 PLA2G2C	2 RELA	1 LMAN1L	1 NCBP2
2 MTNR1B	1 OR2M2	1 GRIN2C	2 LSS	1 SLC24A1	1 RPL12
2 KCNMB3	1 RAB1A	1 GPR44	2 PUF60	1 APCS	1 POU2F2
2 HSD11B2	1 TAF4B	1 IL21R	2 SF3B14	1 GABRA2	1 ARFGEF2
2 HSD11B1	1 DSN1	1 PRSS3	2 CCT3	1 ESRRB	1 DPM2
2 GRM7	1 DPM1	1 KCNK13	2 FBXW2	1 P2RY13	1 CENPI
2 GPER	1 OR5AR1	1 A2M	2 RAE1	1 PTH1R	1 ELL
2 FZD9	1 PPP2CB	1 IKBKG	2 TRAF6	1 SEMA3B	1 TXNL4A
2 F11	1 RRM2	1 LTB4R	2 SNW1	1 KCNQ2	1 CSF2
2 EDNRA	1 TIPIN	1 FCER2	2 KLC4	1 OR10J1	1 PAK2
2 DEFB114	1 ZNF583	1 NRTN	2 CCDC12	1 AXIN1	1 TMEM189.UBE2V1
2 DEFA6	1 OR4C46	1 CHRNA1	2 MED20	1 FMO2	1 RPS6KA1
2 CYP11B2	1 CTNS	1 OPRD1	2 NCOA3	1 LOXL3	1 SLC19A1
2 CALML3	1 HNRNPUL1	1 SCTR	2 ZFP1	1 SYN1	1 PHLPP1
2 ADCY4	1 OR2G3	1 IL28B	2 ADD1	1 AQP3	1 SRF
2 APOA1	1 TRIM28	1 RAC3	2 NXF1	1 BPNT1	1 IFIH1
2 WNT2B	1 PPIA	1 FZD10	2 ITPR3	1 ATP12A	1 ACTB
2 SLC1A6	1 USP18	1 CTRB2	2 SLC7A9	1 CCL13	1 ABAT

2 RXFP2	1 ABCG2	1 TIMP2	2 PTPN13	1 HIST1H2BF	1 IKZF1
2 ABP1	1 PSMD3	1 CXCL6	2 ITGA6	1 CSNK2A2	1 RPS2
2 SLC5A11	1 NRAS	1 XCL2	2 PABPN1	1 ADORA1	1 NUP54
2 SLCO1B3	1 TBL1XR1	1 CAMKK1	2 PPP2R5B	1 HEPH	1 RPS6KA2
2 SLC8A2	1 EIF4G2	1 GNB2	2 ZNF773	1 DYRK1A	1 E2F3
2 SLC22A1	1 GK2	1 LTB4R2	2 ZNF12	1 KCNG2	1 NDUFA2
2 SLC14A2	1 OR8A1	1 COL9A1	2 ZKSCAN1	1 PYY	1 MAP2K3
2 RDH12	1 TBL1X	1 CALML3	2 SUGP1	1 ANGPT1	1 NCK2
2 RAMP2	1 RNF41	1 FGFR4	2 PDK2	1 AQP1	1 UBE3B
2 LRAT	1 CTSB	1 MAPK7	2 NUP88	1 FGF4	1 DHX8
2 LAMA3	1 HEMK1	1 PPY	2 NFATC1	1 FGF13	1 SNF8
2 KDR	1 CASP6	1 ADRB1	2 GTF3A	1 GPX5	1 BCAT1
2 KCNA2	1 MTOR	1 KCNC4	2 CDC7	1 SCN5A	1 REL
2 HSD3B2	1 ATP6V0D2	1 FGF17	2 GTF2F1	1 SLC22A4	1 DLG4
2 HMOX1	1 GTF2H5	1 ADORA1	2 SNRPA	1 FCN3	1 GALNT10
2 HCN1	1 RNASEH1	1 LCK	2 CYLD	1 F2RL2	1 PDE3B
2 GRP	1 MED6	1 GNG4	2 NUP155	1 TEX15	1 PIGL
2 GPR77	1 OR10A3	1 TBXA2R	2 ZNF202	1 ATP6V1B1	1 MAP3K14
2 F2RL2	1 OR56A4	1 DUSP5	2 ZNF418	1 PDGFRA	1 DDX39B
2 DEFA5	1 OR9Q2	1 MYL3	2 MIS12	1 HYAL1	1 ST8SIA3
2 APLNR	1 MTHFD2	1 CD2	2 TRIB3	1 CTSE	1 ATF2
2 BCAR1	1 ZNF286A	1 MUC3B	2 ZNF564	1 KCNH1	1 GTF2F1
2 NPY5R	1 KIFC1	1 SSTR2	2 ZNF2	1 SLC35D1	1 PAPOLG
2 RASGRF1	1 GINS4	1 COL27A1	2 TBXA2R	1 BRAF	1 SRSF1
2 SCT	1 PNLIP	1 RGS4	2 SMC3	1 DRD2	1 DHODH
2 HTR2A	1 PARS2	1 ITPKB	2 SMAD3	1 RGS20	1 AKAP9
2 PLA2G2D	1 UGT2B7	1 KCNK18	2 RPA2	1 TAS2R16	1 PRKCA
2 VIPR2	1 RNF123	1 ADAMTS14	2 RNGTT	1 ANGPTL7	1 TXNRD2
2 NPY2R	1 CBS	1 GALR1	2 PRPF6	1 PLA2G10	1 PANK3
2 COMP	1 ERBB2	1 COMT	2 POLR2G	1 CXCL13	1 CHERP
2 LAMC1	1 H3F3A	1 GNAT1	2 OTUD5	1 CETP	1 MEF2C
2 TNC	1 ARF3	1 ADRA1B	2 ORC2	1 GUCY2D	1 DIS3
2 SLC6A5	1 SHC1	1 RXFP3	2 NUP210	1 RGS12	1 IVD
2 KCNK1	1 SLC1A5	1 MAFG	2 NHP2L1	1 SLCO2B1	1 ATP5G2
2 PDE1C	1 MYBL2	1 GAL	2 DFFB	1 TGM2	1 VAV3
2 SLC4A1	1 GPAA1	1 IL11	2 DCLRE1C	1 CFI	1 EIF2AK2
2 RHAG	1 CASP9	1 GNG2	2 CLCF1	1 GP6	1 NFATC1
2 GJA1	1 CTSL2	1 SSTR3	2 CD79A	1 INSL5	1 TRA2B
2 RHCG	1 RARG	1 CSF1	2 CD19	1 FGD1	1 TANK
2 TAAR8	1 PRKACA	1 RELB	2 CBLB	1 MMP13	1 REST
2 OR1E1	1 RFT1	1 CYP2F1	2 ACD	1 SLC22A8	1 TAF4
2 CHRNA1	1 OR4S1	1 SLC17A7	2 BLM	1 CPB2	1 HMGCS1
2 KCND2	1 OR6F1	1 WNT4	2 RFXANK	1 FZD7	1 RNF41
2 TH	1 IFIT1	1 KCNN1	2 BAD	1 PRKCE	1 OAZ2
2 GPR83	1 SPC24	1 COL7A1	2 POLR2H	1 EDNRB	1 PIK3CG
2 F13A1	1 FZD1	1 RGS11	2 CCR7	1 RARG	1 NUP153
2 GNMT	1 RASGRF2	1 COL9A3	2 RRM2	1 SERPINI1	1 UPF3B
2 CYBRD1	1 ABCA8	1 ENO2	2 ZNF287	1 CRHR2	1 NEDD4
2 KCNJ14	1 MCM7	1 NR4A1	2 WASL	1 NTN1	1 XRCC5
2 FMO4	1 MCM4	1 ADCYAP1R1	2 USP11	1 CYP46A1	1 RANBP2
2 SLC28A3	1 HEPH	1 BAG1	2 LCK	1 GNG13	1 PIGV
2 HTR1F	1 OR2D3	1 GPER	2 UBA7	1 GNA11	1 ARHGEF7
2 SLC7A9	1 OR51A4	1 MAP2K7	2 TBC1D4	1 TGM1	1 PRPF18
2 KCNA5	1 PDPK1	1 WNT10B	2 RFXAP	1 CDH2	1 BRCA2
2 TYRP1	1 UGT2B10	1 AVPR1A	2 ZNF235	1 HLA.DOA	1 PPP2R5C
2 ADRA2A	1 GSTA1	1 NPW	2 TUBGCP6	1 NELL1	1 SLC16A1
2 OR5V1	1 OR10A2	1 FGFR3	2 TAF1C	1 HIST1H2BK	1 NPM1
3 GABBR1	1 DNASE2B	1 NR2F1	2 SUPT5H	1 DRD5	1 GIT2
3 PRLHR	1 LOC391764	1 SLC6A20	2 RBM5	1 GNGT1	1 SH3BP2
3 NUP155	1 OR5D14	1 CBR3	2 METTL3	1 PPP1R12B	1 DAPK3
3 PLA2G1B	1 PPM1B	1 RYR1	2 LIPE	1 ABCG4	1 ATF6
3 OR2M4	1 PRDM9	1 KCNA10	2 ATR	1 ANXA8L1	1 PDE8A
3 SLCO2A1	1 AP1B1	1 ATP1B4	2 HNRNPK	1 DSC2	1 DOK1
3 PRSS3	1 NCAPD2	1 PGAM2	2 TRADD	1 CHRNA1	1 AQR
3 TOP3A	1 SEPHS2	1 HSPA1B	2 TGFBR2	1 COL21A1	1 SRSF3
3 RAD1	1 PKMYT1	1 NR2C2AP	2 STK11	1 SLC24A3	1 POLR2L
3 RMI1	1 SLC6A15	1 ECSIT	2 RBM17	1 FZD8	1 COX6C
3 C17orf70	1 OR4N5	1 EMR2	2 NFAT5	1 KCNK3	1 STAG2
3 PTPN1	1 OR52B4	1 NR2F6	2 CD3D	1 WNT16	1 VDAC3
3 KCNJ3	1 ELOVL2	1 TGFB1	2 IGLL1	1 CDH5	1 GTF3C2
3 SLC12A1	1 OR52B6	1 RAB3A	2 ZNF436	1 EMR3	1 MPI
3 GHRL	1 OR13C8	1 STX10	2 ZNF485	1 SEMA6B	1 DCTD
3 OR3A3	1 OR2K2	1 IHH	2 ZNF585B	1 FTL	1 MAP2K1
3 CYP3A7	1 FYN	1 KCNJ14	2 ZNF433	1 LOX	1 MNAT1
3 ITGA2	1 A1CF	1 NPB	2 ZNF529	1 MMP14	1 PSMD6
3 PTPRA	1 ESPL1	1 PRKAG3	2 CTBP1	1 ABCG2	1 IFIT3
3 SSTR4	1 RASA4	1 CXCR3	2 ZNF248	1 CTTN	1 PTPN11
3 TCIRG1	1 HNRNPA0	1 PFKM	2 FCAMR	1 ARR3	1 NFYB
3 ATP6V1F	1 RHOBTB2	1 PTGS1	2 NOTCH3	1 CYP2A6	1 PCNT
3 GP1BA	1 OR4K13	1 COX6B2	2 EHD1	1 KCNJ8	1 FBXW2
3 NUPL2	1 ADCY5	1 ST6GALNAC2	2 CEP250	1 CYP27B1	1 RAC1
3 ALDH7A1	1 PIK3R6	1 PDYN	2 CRADD	1 FERMT2	1 TUBA3C
3 FZD7	1 HNRNPK	1 EPO	2 DHCR7	1 CHAT	1 EIF3J

3 ATP10A	1 ZNF514	1 GADD45A	2 HMGCR	1 OR7C1	1 NEIL3
3 KCNK6	1 ZNF419	1 GAPDHS	2 ZNF37A	1 S1PR4	1 LGMN
3 P2RX1	1 UNG	1 IL8	2 ERCC3	1 COL19A1	1 GMPS
3 GLRA1	1 FANCA	1 CRHR1	2 SF3A1	1 TNN	1 UBE2N
3 SLC30A1	1 H2AFZ	1 GNA15	2 COL5A1	1 ATP6V1G2	1 C1GALT1C1
3 GSTM5	1 SATB1	1 OPRK1	2 BAX	1 KCNMB4	1 COX15
3 SLC5A8	1 MLF1IP	1 ICAM3	2 ZNF212	1 ACSM2B	1 ASB13
3 CYP11A1	1 ZKSCAN5	1 SLC18A3	2 B3GAT3	1 HCRT	1 CCND2
3 EGF	1 LIN9	1 GPR55	2 EBP	1 FBLN1	1 BMI1
3 ALDH3B2	1 PIGK	1 IL28A	2 CD8B	1 SERPINH1	1 GEMIN6
3 KCNB1	1 ARHGDIB	1 CSF2	2 MAP4K1	1 HIST1H4I	1 CD28
3 SLC47A1	1 PTPN13	1 MUC13	2 HNRNPUL1	1 OPN1MW2	1 ACADSB
3 OR1G1	1 GINS2	1 INSL3	2 POLR2B	1 ISL1	1 SNAPC1
3 GHR	1 RPA4	1 OXT	2 HIST1H1E	1 SERPINB10	1 DCPS
3 GUCY2D	1 C19orf40	1 SCT	2 ZNF324	1 SAG	1 ITGAV
3 OR6A2	1 MAN1A2	1 APOE	2 DDX42	1 STEAP3	1 CDC42
3 SLC7A1	1 GTF3C2	1 GNG13	2 HDAC1	1 KCNV2	1 MALT1
3 OR2W1	1 NT5C2	1 COL15A1	2 FDPS	1 CILP	1 SLK
3 SARDH	1 CSTF1	1 MAOB	2 CEP164	1 DAB2	1 TRA2A
3 LEPR	1 CAD	1 ADRA1D	2 SNRNP200	1 KCNA3	1 RPA2
3 SORBS3	1 FANCG	1 FLT3	2 CDK11B	1 TLE2	1 DNAJC8
3 KCNJ2	1 MAT2B	1 CX3CR1	2 FLOT2	1 SERPINA3	1 POLR2H
3 RGR	1 MARS	1 KCNG4	2 CYP2J2	1 UNC5C	1 PPIA
3 FANCM	1 SLC1A4	1 CD4	2 SRSF1	1 CLDN18	1 ABI1
3 SLC6A3	1 RNF6	1 TPSAB1	2 RAG1	1 CLTCL1	1 STAG3
3 AQP9	1 SMC1A	1 GNG8	2 ZNF92	1 TAS2R14	1 SRSF9
3 CNGA4	1 OR6N2	1 HSPA1A	2 HRAS	1 CA5B	1 EIF4E
3 ATRIP	1 SLC27A4	1 SSTR4	2 DIDO1	1 ADH7	1 POLR2D
3 PHGDH	1 TBPL1	1 COL17A1	2 ZNF606	1 TBXA2R	1 ORC3
3 UCHL1	1 CCNA2	1 SLC25A5	2 IP6K2	1 CP	1 TBPL1
3 SLC2A6	1 MLEC	1 PLA2G2F	2 ZNF425	1 MARCKS	1 NCR3
3 ICAM1	1 PDIA3	1 GFRA4	2 ZNF510	1 MAPK8	1 ATP5A1
3 INSL5	1 CALM1	1 KCNV2	2 DEDD	1 ADORA3	1 POLR1E
3 GNGT1	1 VHL	1 KCNG2	2 E2F5	1 BRINP2	1 PSMC5
3 LAMC2	1 ZNF558	1 PTGER4	2 LTB	1 PARD6B	1 ATP5C1
3 ITGA9	1 HNRNPD	1 PLTP	2 IPCEF1	1 HSD11B1	1 CD8B
3 SLC8A1	1 OR2T1	1 SLC6A1	2 DOCK9	1 SLC4A3	1 FANCF
3 KCNH3	1 TAF4	1 PTPN5	2 FYN	1 SNCA	1 STRAP
3 KCNK3	1 UBA7	1 ADRA1A	2 ZNF211	1 GRIN2B	1 SMNDC1
3 GRM4	1 ZNF445	1 KCNQ4	2 MYBL1	1 DRD1	1 SNAPC3
3 PVRL1	1 ZNF430	1 CYP1A2	2 PARP1	1 SERPINA7	1 CASP6
3 GABRA5	1 CENPH	1 OPN1LW	2 PLRG1	1 FGF21	1 CCT6A
3 LTB4R	1 PIGY	1 COL11A2	2 KIF2A	1 RELN	1 MAPK13
3 SCTR	1 KIF23	1 NMS	2 ZNF484	1 CYP39A1	1 POLR3F
3 DEFB4A	1 OR52N2	1 IL29	2 ITK	1 CREB3L1	1 MAN1A2
3 CHRNA5	1 LMNB1	1 CCR6	2 WBP11	1 OR2W1	1 CDCA8
3 ALDH3A1	1 ZNF267	1 CNGA1	2 PRPF3	1 CCK	1 PSIP1
3 SLC18A1	1 ABCA10	1 LAT	2 SPTAN1	1 ABCA8	1 CETN2
3 GNRH1	1 PSMA5	1 PLAU	2 MED25	1 PTGER3	1 DKC1
3 SLC3A1	1 ABCB1	1 FGF5	2 RAC2	1 NR0B2	1 SLC25A13
3 ASPA	1 RPA3	1 GNGT1	2 MED14	1 UGT1A7	1 EIF4A1
3 ATP6V0A4	1 HIST1H4L	1 IL12A	2 NCOA1	1 P2RY4	1 IFITM1
3 CYP26A1	1 MCM5	1 GPR35	2 PHF5A	1 CYP4F3	1 RFC1
3 MDM2	1 LIG4	1 OPN1MW	2 PSMD4	1 BMP10	1 UQCR10
3 GCG	1 OR56A1	1 HNF4A	2 CASP3	1 ERBB2	1 RNMT
3 OR11A1	1 AURKA	1 JAM3	2 GTF3C3	1 SERPINI2	1 YWHAQ
3 FSHB	1 PANK2	1 RHO	2 NSMAF	1 ALOX15B	1 DDX46
3 GRIA4	1 HIST1H4F	1 ALOX15	2 MST4	1 TCHH	1 CAD
3 EMR3	1 TPK1	1 DDIT3	2 ZNF23	1 SEMA4G	1 ATP5B
3 GRIA3	1 TUBA3C	1 EVL	2 PPP2R2A	1 GJB4	1 ATP5G1
3 SLC17A8	1 UBA1	1 FGF21	2 ELL	1 LTBP2	1 POLD3
3 F10	1 SKIL	1 KCNK3	2 FGFR1	1 CTBP2	1 RALB
3 NPFFR2	1 IL4	1 HCN2	2 GNB5	1 SLC9A7	1 ATG7
3 SLC6A9	1 ZWINT	1 CHRNA6	2 ZFP2	1 ADCYAP1	1 PALB2
3 NPBWR1	1 BUB1	1 IL5RA	2 ZNF643	1 COLEC12	1 ATP6V1A
3 KCND1	1 PSMD2	1 DEFB132	2 EZR	1 EMR1	1 LSM5
3 GRID1	1 POLE	1 KCNN3	2 PLCG1	1 SLC34A2	1 IFNAR2
3 HSD17B6	1 RAP1A	1 IL17A	2 ZNF343	1 SLC12A6	1 HNRNPD
3 GALR1	1 ZNF250	1 MYLK2	2 DYRK1A	1 CLCA1	1 NUP155
3 CYP7B1	1 FUS	1 F10	2 ZNF17	1 CYP4A22	1 NBN
3 FZD2	1 ALG1	1 FGF22	2 CCT7	1 SHC3	1 EIF4G1
3 RDH5	1 TIRAP	1 CNTN2	2 POLR2C	1 ADRA1D	1 HLA.DRB1
3 SLC32A1	1 CCNB1	1 ACE	2 CDC5L	1 OR2B6	1 IFITM3
3 DEFB125	1 ZNF354B	1 NGF	2 MVD	1 DSG2	1 KIF18A
3 KCNH8	1 HIST1H3F	1 CYP21A2	2 BTRC	1 CHRNA3	1 PARN
3 ITGB1	1 HNRNPM	1 SST	2 STIM1	1 WASL	1 EIF2B3
3 ABCC11	1 PSMD11	1 MUC5B	2 ACACA	1 SLC17A5	1 OAS1
3 AQP1	1 CDC25A	1 SLC6A11	2 ZNF300	1 CALCB	1 ACTR3
3 GRIN2A	1 ZNF45	1 COL8A2	3 FUS	1 IFNA21	1 SLBP
3 RASGRF2	1 SNRPD1	1 CTSG	3 KPNB1	1 CHAD	1 IL12RB2
3 CHRNB4	1 ZNF354A	1 RDH12	3 MEN1	1 HSD11B2	1 EIF3B
3 ABCA2	1 POLR3H	1 APOC2	3 CEL	1 MASP2	1 LARS
3 CACNA2D3	1 POLR3G	1 SLC8A2	3 ZNF43	1 PRDM9	1 ALG6

3 PIK3R3	1 SLC41A1	1 ESR1	3 ZNF625	1 GPX3	1 NDUFA6
3 ABCG1	1 FEN1	1 NFKBIA	3 IL27RA	1 PGR	1 CNOT2
3 GLRB	1 DOLPP1	1 ALOX15B	3 C8G	1 CYP3A43	1 YWHAH
3 PENK	1 PRF1	1 CD14	3 GRK4	1 PTN	1 SUPT16H
3 TRPV1	1 ACSL4	1 HCN4	3 GTF3C2	1 CPA3	1 PDK1
3 CBS	1 HNRNPU	1 FLNC	3 PRKX	1 FGF20	1 POLE3
3 PRKCB	1 GEMIN6	1 GABBR2	3 MAML1	1 TAS2R3	1 BIRC5
3 CHRNB1	1 CCNB2	1 CYP11B2	3 MKNK2	1 CHRNA10	1 ORC2
3 CYP2D6	1 HIST1H2AB	1 ATP1A3	3 POLD2	1 NPY5R	1 MAGED1
3 PTGER1	1 FANCB	1 MMP25	3 ZNF263	1 AKT3	1 IL21R
3 MET	1 RRN3	1 PRG2	3 IDI1	1 CHP2	1 POU2F1
3 GRIK5	1 STAG2	1 AMN	3 NOP56	1 GNAS	1 CLNS1A
3 SLC9A9	1 ZFP28	1 DHH	3 KIRREL	1 MMP19	1 CNTRL
3 KCNK2	1 DDX23	1 KLB	3 SLC25A20	1 NID1	1 TGFBR1
3 DMGDH	1 UBE2Q2	1 CCL21	3 PRDM9	1 TREM2	1 PANX1
3 SHMT2	1 HIST1H3G	1 TPM1	3 RAP1B	1 SLC28A3	1 ABL1
3 GJB6	1 KPNA5	1 HCK	3 UBTF	1 GH1	1 LARS2
3 ATP7B	1 RIPK1	1 HTR6	3 KIF3C	1 IL19	1 THOC2
3 SLC44A1	1 HSD17B4	2 LHCGR	3 SREBF2	1 CNGB1	1 CDC23
3 CHAT	1 ITGAV	2 MOS	3 NCK2	1 SLC6A7	1 IMPDH2
3 KCNJ11	1 SLC15A2	2 CRH	3 STK24	1 GUCA1A	1 NFKB1
3 PLCB4	1 ZNF643	2 WNT8B	3 ZNF154	1 MFGE8	1 APC
3 SLC9A5	1 KLF8	2 P2RY1	3 ZNF274	1 TNXB	1 ITGA4
3 MYLK	1 TUSC3	2 GP5	3 DAXX	1 INHBB	1 MSH6
3 NUP43	1 MSH6	2 LTA	3 PPP1R13B	1 STAR	1 MCCC2
3 KLK6	1 OR56B4	2 ITGA7	3 ZAP70	1 WNT2	1 SMC3
3 SLC37A4	1 NRG1	2 NR1D1	3 PRPF8	1 EPB41	1 STAT3
3 CHRNA6	1 LARS2	2 PPBP	3 ZNF225	1 ACACA	1 TUBB
3 TAS1R1	1 SORT1	2 KCNA3	3 PARD6A	1 ADRA1A	1 NUP93
3 SLC15A4	1 CCT5	2 MUCL1	3 NUP214	1 MYL10	1 CASP3
3 SLC7A11	1 POLA2	2 FGF10	3 KIFAP3	1 CDH9	1 MAP2K7
3 AQP6	1 PSMC2	2 GABRA1	3 STK36	1 WNT8B	1 METTL3
3 ADCY9	1 ZNF226	2 IL22RA2	3 ENDOD1	1 FGF16	1 MYD88
3 GNAL	2 ABCB4	2 ACTC1	3 TH1L	1 VIPR2	1 ITPA
3 DEFB1	2 UGT2B17	2 SAG	3 CDK7	1 F13A1	1 CD74
3 KCNG4	2 ZNF585A	2 GGT6	3 CTDP1	1 ADRB3	1 IRF2
3 CYP21A2	2 CENPN	2 KCNQ1	3 RPS6KA1	1 RAMP3	1 GANAB
3 ICAM4	2 ZNF304	2 ADCY4	3 POLE2	1 TGFBI	1 CD7
3 HTR4	2 NBN	2 GNRHR	3 APITD1	1 ADAMTS12	1 MTA1
3 GLDC	2 BRCA1	2 OPN3	3 CENPO	1 AKAP5	1 ATP2A3
3 SLC15A3	2 MAT2A	2 EDA	3 MAGOH	1 HIST1H2BO	1 CALR
3 SLC28A1	2 AP3M2	2 LPAR4	3 HIST1H4H	1 SV2B	1 PARVB
3 THBS3	2 RAD51	2 PMM1	3 GZMA	1 TGFA	1 TLN1
3 ABCA3	2 ZNF564	2 C3AR1	3 PTPN1	1 CDH18	1 UCP2
3 AOX1	2 PAFAH1B1	2 COX4I2	3 POLR2J	1 PRL	1 TGS1
3 CYP4A22	2 SGOL1	2 FLT3LG	3 TERT	1 COLEC10	1 NME4
3 PTCH2	2 TFDP2	2 GRK1	3 CYP1A2	1 RDH16	1 CD55
3 OR2B3	2 ACSL5	2 IL6R	3 MAP3K10	1 GSTT2	1 DARS
3 ATP2B3	2 POT1	2 FGF11	3 MVK	1 SLC44A4	1 HLA.DQA1
3 HCN3	2 PIK3CB	2 CYP3A7	3 SF3B3	1 OXT	1 HLA.DRB5
3 GABRG1	2 ZNF480	2 LPAR3	3 ORAI1	1 ARHGEF4	1 CTSO
3 PROS1	2 ZNF222	2 DEFB127	3 PHKA2	1 IFNA16	1 KPNB1
3 SLC44A3	2 PIK3R3	2 MMP8	3 MAPK1	1 IFNA17	1 GALNT12
3 FZD10	2 GTF2B	2 SLC18A1	3 MAPK3	1 PLA2G1B	1 MCM4
3 SLC13A1	2 ZNF398	2 IL3RA	3 TNFRSF1B	1 MUC3A	1 TPX2
3 HSD3B1	2 SMC4	2 RDH8	3 HSD17B7	1 GABRB1	1 MAN1A1
3 GALR3	2 MAD2L1	2 HRH1	3 MTOR	1 OR1D2	1 PSME3
3 SNAP25	2 SHMT1	2 IFNA1	3 MED1	1 NAT2	1 KIT
3 STAT5B	2 CTSS	2 P2RY12	3 KPNA3	1 ROCK2	1 KLRC3
3 GP6	2 POLD3	2 RXRG	3 PIK3R5	1 OR1E2	1 CCR7
3 SLC34A3	2 ZNF92	2 ABCA1	3 HIST2H2AC	1 CA8	1 DSE
3 SLC39A6	2 EIF5	2 DEFB121	3 ZNF169	1 GRP	1 RNF138
3 SLC16A8	2 RAD50	2 CACNG3	3 PPP2R5D	1 SLC5A1	1 SEH1L
3 SLC25A10	2 OR2B6	2 SLC44A5	3 CARD11	1 TCF7L2	1 IDH3A
4 PLA2G6	2 UBE2T	2 PCOLCE2	3 CD247	1 CACNB1	1 STAG1
4 CGA	2 CCNE1	2 PTHLH	3 ZNF3	1 GRIN1	1 MICB
4 MGST3	2 CLSPN	2 CCL27	3 DNM2	1 P2RY2	1 PAK1
4 SLC35A3	2 ZNF311	2 ABAT	3 PRPF4	1 LAMA1	1 PDHX
4 PRKACB	2 CENPQ	2 DEFB118	3 TERF2IP	1 NPY1R	1 USP1
4 TPM3	2 DBF4	2 UGT2B15	3 CCL24	1 SLC2A10	1 DDB1
4 ADH5	2 GTF2H3	2 GLS2	3 EGLN3	1 SLC39A2	1 CCR5
4 CTNND1	2 MOCOS	2 RASGRP4	3 RARG	1 HSP90AA1	1 CTSC
4 AQP4	2 GTF2E1	2 GAD1	3 PXN	1 CHRNA5	1 GRK5
4 IBSP	2 ZNF607	2 NR4A2	3 PPP2R5C	1 KAL1	1 IL27RA
4 NUP133	2 TAS2R10	2 GRIN2B	3 YWHAQ	1 GIP	1 NHP2L1
4 KCNH7	2 DZIP3	2 CYP11A1	3 SH2B1	1 INS.IGF2	1 CD40LG
4 ATP6V1D	2 CSTF3	2 RGS9BP	3 SF3B2	1 L1CAM	1 NOP56
4 SLC38A1	2 KIF15	2 CXCL3	3 PIK3CD	1 OR7A5	1 ITPR3
4 MYL6	2 HIST1H3E	2 KCNMB1	3 FASLG	1 CTRC	1 MDC1
4 FTCD	2 PRKCQ	2 NTSR2	3 GNAS	1 GHRHR	1 EGR1
4 OR1Q1	2 CDC23	2 DRD1	3 ZNF587	1 GLI2	1 SHC1
4 GSTK1	2 RPS6KA3	2 FGF9	3 NR2C2	1 PRELP	1 MGAT4B
4 PRKAR1A	2 PIGW	2 KCNJ6	3 CPT1C	1 COL11A1	1 MOGS

4 COL18A1
4 ITPR2
4 CP
4 FBXW11
4 GNAQ
4 RPA1
4 DDC
4 WNT10A
4 FANCI
4 KCNH2
4 FKBP1A
4 SLC26A1
4 PARK2
4 GPR17
4 GRM2
4 ATP1B3
4 FANCB
4 ADCY1
4 ATP4B
4 OR1J2
4 DNM2
4 LCMT2
4 KCNH6
4 ATP11A
4 ABCA4
4 GRIK4
4 ROCK2
4 SLC30A7
4 TFRC
4 ATP11C
4 PIK3CB
4 CDH1
4 WDR48
4 ART1
4 ANXA1
4 GJB3
4 MCHR1
4 FN1
4 RFC3
4 INSR
4 PTGER3
4 CHRNB2
4 NCALD
5 NBN
5 MC1R
5 CD36
5 RRAS
5 CAMK2D
5 SLC6A11
5 PPP1R12B
5 COL4A6
5 UGT2A3
5 CACNB1
5 SORBS1
5 H2AFX
5 KCNMB4
5 DEFB127
5 ATP2A1
5 CDH11
5 SLC24A1
5 PLA2G12A
5 PRKG1
5 GABRA1
5 SLC40A1
5 ADAM28
5 KCNS2
5 STEAP3
5 PIK3R2
5 CAMKK1
5 CLTB
5 CYP17A1
5 PLCB1
5 SLC4A7
5 ABCB6
5 LMOD1
5 SLC39A5
5 GPR156
5 FANCA
5 ATP6V0D2
5 CDH15
5 COL7A1
5 DNM1
5 DEFB126
2 AGL
2 CROT
2 ALG11
2 ANAPC1
2 LCMT2
2 ZNF175
2 FANCM
2 RPS6KA6
2 CDC7
2 OR2D2
2 SKP2
2 ZNF600
2 ZNF493
2 MRE11A
2 ZNF248
2 MND1
2 ABCD3
2 BLM
2 RFWD2
2 BRIP1
2 CENPI
2 PEX3
2 TAS2R9
2 MED23
2 TAS2R14
2 TAS2R31
2 ZNF595
2 TAS2R3
2 ZNF528
2 PCF11
2 ZNF223
2 ITPR2
2 TTK
2 ZNF655
2 ZNF320
2 ZNF658
2 SPC25
2 TAS2R20
2 OR6A2
2 TAS2R50
2 CDC6
2 FANCL
2 PIGN
2 GPAM
2 ZNF347
2 TAS2R13
2 ZNF234
2 BRCA2
2 ZNF141
2 MBD4
2 SLCO1B3
2 ZNF484
2 CENPE
2 POLE2
2 ZNF225
2 RFC4
2 XRCC2
2 SLC26A2
2 DCLRE1C
2 PTTG2
2 CCNE2
2 CTSK
2 CEP290
2 EIF2AK2
2 TAS2R19
2 APC
2 TAS2R4
2 ACACA
2 HIST1H3A
2 KIF18A
2 TAS2R46
2 MSH2
2 ZNF215
2 PGM3
2 ZNF28
2 SEPSECS
2 GCLM
2 MCM8
2 CDC26
2 CCDC99
2 FANCD2
2 FBXO5
2 MCM10
2 PGR
2 EMR1
2 IL27
2 GFRA1
2 IL3
2 ELMO1
2 GRIA3
2 HLA.DPA1
2 CCL11
2 CXCL10
2 UQCRB
2 DNTT
2 MC2R
2 MAS1
2 GNG3
2 IL2
2 ICAM2
2 RXFP2
2 MUC17
2 SLC6A5
2 DEFB110
2 KCNH6
2 MYL2
2 EDAR
2 IL12B
2 TGFB3
2 NPSR1
2 COX8C
2 AVPR2
2 IFNE
2 DEFB119
2 EDN1
2 CXCR4
2 TSHB
2 FBP2
2 HLA.DQA1
2 RGS18
2 CD1B
2 FCGR3A
2 FGF14
2 GADD45B
2 CD3D
2 COX7B2
2 CCR1
2 NR4A3
2 CALCB
2 COL8A1
2 CYP8B1
2 ITGA10
2 PRL
2 HLA.DMA
2 IFNA5
2 CACNG1
2 TDGF1
2 GYPA
2 PLA2G2A
2 TNFSF11
2 CACNB4
2 CYP2A13
2 DEFB113
2 KCNK10
2 ABCG1
2 PLA2G1B
2 CYP17A1
2 CHP2
2 SCARB1
2 GABRQ
2 GPR68
2 GRK7
2 ITGAX
2 CACNA1I
2 CNR2
2 GRM5
2 THRA
2 GP1BA
2 CCL26
2 ITPR1
2 MAF
2 CYP4B1
2 LCP2
2 AGT
2 GLT25D2
2 GABRD
3 CFLAR
3 CDKN2D
3 AKT2
3 BGN
3 XIAP
3 TBXAS1
3 SUN2
3 SOCS4
3 SLC2A1
3 SLC1A7
3 SF3A2
3 RORA
3 PRKCQ
3 PRF1
3 PREX1
3 PDGFRB
3 PAPOLA
3 NCOR1
3 MED16
3 KIF3B
3 KIF26A
3 HIST1H3I
3 FOXO3
3 EIF4G3
3 DAPK3
3 DAPK2
3 CYP7B1
3 CYP4F2
3 CHST12
3 CDKN1B
3 CD8A
3 CCR4
3 CCL5
3 CCL25
3 B3GAT2
3 BCR
3 EDA
3 LAMC2
3 ACIN1
3 PRKAB1
3 EXOC7
3 CPT1B
3 SHC1
3 MAP2K1
3 TINF2
3 CHPF2
3 DHCR24
3 NRG2
3 CKAP5
3 RXRB
3 FBXW4
3 ZNF434
3 ZNF45
3 RAF1
3 DDX23
3 MCM3
4 SLC6A6
4 TRIP10
4 TRAF2
4 PDK1
4 TNFRSF4
4 HIST1H2BJ
4 LAMC1
4 EHD2
4 H1F0
4 HIST1H2AC
4 MSMO1
4 CXCR6
4 LAMA5
4 NDRG1
4 NQO1
4 CCL17
4 CDH1
4 INHBA
4 TGFA
4 PDGFA
4 ENG
4 PPBP
4 VDR
4 RELB
4 PFKFB3
4 MAP3K4
4 IL3RA
1 GUCY1B3
1 IL36RN
1 PRKAB2
1 SLC5A5
1 THBS1
1 WISP3
1 ABL2
1 ITIH2
1 ASIP
1 ANXA10
1 TACR3
1 GUCY1A3
1 KCNMB1
1 OR2C1
1 IFNA5
1 MAT1A
1 COL4A2
1 IL24
1 TBXAS1
1 UNC13B
1 GPR50
1 CST8
1 GLYAT
1 FLG
1 SLC11A1
1 FGF14
1 IL1B
1 LAMC1
1 FCGR1A
1 SLC4A8
1 S100A5
1 TAS2R9
1 ERBB3
1 F9
1 SLC7A8
1 CACNB4
1 FPR3
1 CYP11A1
1 NPY2R
1 COL11A2
1 BDKRB1
1 USH2A
1 HIST1H2BH
1 SLC7A9
1 TIAM2
1 ACTN3
1 KCNQ3
1 CYP11B1
1 OR1G1
1 PENK
1 LGI1
1 PAMR1
1 TRPV1
1 IFNA14
1 EMCN
1 GABRA4
1 HPSE2
1 NR5A2
1 RYR3
1 TCF7L1
1 SCN4A
1 CD36
1 PRSS1
1 CGA
1 GABBR1
1 FZD3
1 FBN2
1 SLC22A11
1 GABRB2
1 GSTA1
1 IL1RN
1 SLCO1C1
1 S100A7
1 TAS2R1
1 NDNF
1 COL2A1
1 CTRB2
1 EPHX2
1 MMP12
1 EPHX1
1 FRZB
1 HPX
1 NOV
1 PPP1R13B
1 PLEC
1 MYH9
1 SOS1
1 ARPC1A
1 PPP2R5D
1 STAT5A
1 WDR61
1 B4GALT5
1 SRRM1
1 E2F1
1 SIPA1
1 TACC1
1 ENO1
1 CFL1
1 SF3B4
1 PIGT
1 PTBP1
1 NUP210
1 OGDH
1 HDAC3
1 PPP3CB
1 VPS37B
1 ANAPC2
1 UBA1
1 PRPF8
1 PTPN6
1 TGFB1
1 AKT1
1 CISH
1 SNRPA
1 VASP
1 ARPC2
1 ITGB7
2 STAT4
2 CEP76
2 CASP1
2 CD19
2 HNRNPA1
2 XAF1
2 HSPA9
2 IDH3G
2 IL15
2 NME1.NME2
2 MTMR4
2 POLR3C
2 COX7B
2 DHX9
2 EIF3I
2 MOCS2
2 HNRNPM
2 MAPKAP1
2 PPP1CC
2 RBMX
2 ALG8
2 NUP107
2 IRAK4
2 DZIP3
2 USP6NL
2 APH1A
2 NACA
2 HRAS
2 POLR2J
2 PAPD7
2 EDEM1
2 RBCK1
2 SNRPB2
2 AP1B1
2 SRP54
2 ATP6V1H
2 PSMB6
2 HNRNPR
2 RAD51
2 NDUFV2
2 CDK7
2 GTF3C3
2 NDUFA8
2 UBE2E1
2 PSMD12
2 MT2A
2 PSMA7
2 ATP5G3
2 NCL

5 SLC6A20	2 KIF11	2 PLCG1	4 LAMB3	1 SLC18A1	2 SLC25A5
5 RAMP3	2 PCNA	2 SHC2	4 HSPA2	1 CACNG2	2 SLC2A3
5 KCNJ16	2 PDE3B	2 ATP2B3	4 RAB27A	1 SERPINA4	2 PPP3CC
5 CYP3A5	2 SLC30A5	2 CD8B	4 TNIK	1 SLC1A1	2 SNRNP27
5 MRVI1	2 NEIL3	2 CYR61	4 SREBF1	1 THY1	2 APRT
5 GLYCTK	2 RBBP8	2 KCNG3	4 SPHK1	1 FGF23	2 CDC16
5 CADM1	2 NCAPG	2 FGF1	4 SLC7A11	1 NTSR2	2 SF3A3
5 EIF2AK2	2 CENPK	2 HLA.DQB1	4 SLC16A10	1 HIST1H4F	2 FARSA
5 ABCD2	2 DDX58	2 HTR1A	4 SDC2	1 IGFBP3	2 PSMD2
5 PLA2G12B	2 TLR4	2 PDGFRB	4 S100A12	1 CUBN	2 COX7A2
6 CSH1	2 SKA2	2 NOS1	4 NKX3.1	1 HABP2	2 UBE2M
6 OXTR	2 GINS1	2 ST3GAL1	4 MT2A	1 PDE11A	2 DYNC1I2
6 STX6	2 MBOAT1	2 CYP2A6	4 KIFC3	1 OR10H3	2 MAN2A1
6 HCRTR2	2 MAPK14	2 COX5B	4 IL26	1 ALDH1A3	2 NFX1
6 POM121	2 PARP1	2 FMO2	4 IL1B	1 GAD2	2 RPL6
6 CYP7A1	2 ENTPD5	2 DUSP16	4 HIST3H2A	1 LEPR	2 NAMPT
6 KCNV2	2 NUDT12	2 NFKB1	4 HIST2H2AA4	1 HIST1H2BM	2 RUVBL2
6 OR8D1	2 OR2M5	2 PRKX	4 HIST1H2AE	1 ARTN	2 PRPF40A
6 UGT1A6	2 KIF20A	2 FGF3	4 HIST1H2AD	1 ATP4B	2 DPM1
6 ITGA10	2 ALG5	2 PPYR1	4 HIF1A	1 AVPR1A	2 GRPEL1
6 NMUR2	2 RARS	2 NPBWR2	4 FIGF	1 BCL2	2 SDHC
6 CYP2A7	2 TGS1	2 IFNA13	4 EREG	1 CLDN10	2 MLH1
6 FZD6	2 ZNF224	2 GHRL	4 CYP1B1	1 NTNG1	2 GNB1
6 SLC9A3	2 TPMT	2 HSPA6	4 CXCL1	1 SLC38A1	2 NDUFB4
6 OPN4	2 GMNN	2 IL2RA	4 CHST15	1 OR1F1	2 SRP19
6 GPR35	2 HIST1H2BJ	2 NR3C2	4 CCL7	1 ARSB	2 ATP5L
6 MYLK3	2 IDH1	2 NPBWR1	4 CCL23	1 IFNA13	2 CCT7
6 CHRNA2	2 ZDHHC21	2 SLC44A4	4 CCL18	1 C8B	2 CSNK2A1
6 ABCC5	2 KNTC1	2 LRAT	4 CACNA1G	1 TAAR2	2 UQCRFS1
6 CADM2	2 SLC16A7	2 HCRTR1	4 BIRC3	1 ITGA6	2 ASB1
6 FGG	2 HERC4	2 ATF3	4 BHLHE41	1 NRCAM	2 MAPK1
6 AGXT2	2 RNGTT	2 DUSP1	4 BCL6	1 SLC6A12	2 MUT
6 CYP19A1	2 PIGM	2 DUSP10	4 ACSL1	1 SEMA7A	2 FYN
6 OR7A10	2 TOPBP1	2 F2	4 ADM	1 PRKAR2B	2 TPR
6 GNB4	2 ITGB3BP	2 NR1I3	4 WNT5B	1 ADAMTS2	2 ATP6V1C1
6 SLC6A1	2 ZNF273	2 CD40LG	4 VCAN	1 C6	2 MANEA
6 CYP11B1	2 ROCK2	2 DUSP9	4 THBS1	1 NEU3	2 ACTR2
6 BHMT	2 ZWILCH	2 FAS	4 SLC32A1	1 IFNB1	2 DCK
6 KCNH4	2 GK	2 GRIK2	4 NCS1	1 HTR1B	2 SPCS3
6 CDH13	2 ABCA12	2 P2RX7	4 MAMLD1	1 AKR1C2	2 ALG13
6 NEFL	2 CEP70	2 CAMK2D	4 IL8	1 GNA14	2 PMAIP1
6 GSTA1	2 KIF4A	2 NR1D2	4 IL1A	1 SLPI	2 MAP3K5
6 ADH1A	2 TRMT11	2 CACNG2	4 IL17F	1 LNPEP	2 UBE2G1
6 PVR	2 ZNF10	2 HTR7	4 HIST1H3D	1 CYP3A5	2 USP39
6 ATP9B	2 SLC12A2	2 MAP3K4	4 HIST1H2BN	1 COL5A3	2 ATM
6 RFC5	2 ZNF519	2 PLA2G5	4 HIST1H2BH	1 AMBN	2 TGIF2
6 SLC12A4	2 TFAM	2 STX8	4 HIST1H2BG	1 AQP7	2 PSMC6
6 HSD17B2	2 CEP76	2 EDN2	4 HIST1H2BF	1 ACTN2	2 CTSH
6 THRB	2 MFSD8	2 PLCE1	4 HIST1H2BD	1 FZD4	2 GALNT7
6 ADH1B	2 DNA2	2 MYC	4 HIST1H2BC	1 GCG	2 CEP135
6 WNT3	2 CUL3	2 PHKG1	4 HIST1H1C	1 KCNJ14	2 APPL1
6 PLCB3	2 RPE	2 PHPT1	4 GADD45G	1 HTR2C	2 PPP1R12A
6 AVPR2	2 ZNF138	2 IL20RA	4 FLT1	1 TYRP1	2 HDAC1
6 GAL	2 NUF2	2 TNNT1	4 EBI3	1 SRGAP3	2 NPEPPS
6 PDGFB	2 SESN1	2 GCGR	4 CXCL5	1 APOC3	2 IFNGR2
6 SLC27A4	2 SLC33A1	2 CYP4F8	4 CXCL3	1 ADAMTSL3	2 TACC3
6 FBN1	2 MLH3	2 GHRHR	4 AGFG1	1 FGFR2	2 SUCLA2
6 OR2C1	2 UGGT2	2 CRKL	4 CCL20	1 PPY	2 SUPT3H
6 KCNC3	2 ZNF587	2 TLL2	4 HIST2H2BE	1 GNA13	2 PSMD4
6 SLC46A1	2 GNPNAT1	2 RLN3	4 CDK5RAP2	1 HIST1H3J	2 MPG
6 HCN4	2 NEDD4	2 CLCF1	4 JAK3	1 TLL2	2 VPS4A
6 CACNA1B	2 ZNF468	2 ADM	4 HIST3H2BB	1 HTR1F	2 POLR2B
6 COL4A2	2 HIST2H4A	2 COL23A1	4 HIST1H2BM	1 SLC5A7	2 SNRPD2
6 OR6B1	2 ATR	3 GUCA1B	4 HIST1H2BL	1 CD59	2 ATP5J
6 FANCE	2 EDEM3	3 PIK3R5	4 HIST1H2BI	1 CAMK4	2 TOMM40
6 THBS4	2 ZNF492	3 HK2	4 HIST1H2BK	1 VIP	2 TBCB
6 CDH8	2 EXO1	3 NGFR	4 SQLE	1 VWA5A	2 EIF4E2
6 KCNF1	2 GTF2H2	3 TNF	4 CYP27A1	1 SLC17A1	2 NFKBIE
6 LHB	2 ZNF605	3 IL28RA	4 CASP7	1 MYC	2 OAZ1
6 CCR2	2 SGOL2	3 ITGA11	4 TCF7L2	1 CNGA3	2 GTF2H3
6 HTR1E	2 SOS2	3 MLN	4 AICDA	1 MEP1A	2 RASGRP1
6 CLTCL1	2 CHUK	3 KCNK9	4 SDC1	1 TECTA	2 SLC25A6
6 FZD8	2 MOCS2	3 WNT5A	4 VAV1	1 GNAO1	2 NMI
6 SLC17A7	2 PIGF	3 RGS6	4 SRC	1 NMBR	2 POLR2F
6 BRCA2	2 NME7	3 GRM2	4 CEBPB	1 FSHB	2 RELB
6 CDH6	2 TPTE2	3 EDA2R	4 CD44	1 LRP2	2 HLA.DRB4
6 ADRA1B	2 DGKH	3 KCNJ12	4 CXCL16	1 WNT6	2 PPP1CB
6 RIMS1	2 POLR2B	3 HTR2B	4 IL12A	1 SLC6A9	2 NDUFB6
6 GRIK2	2 CASP1	3 OSM	4 PPP1R3B	1 CHRM2	2 ATP5J2
6 SLC2A8	2 CASP8	3 AVPR1B	4 CYP27B1	1 CACNA1H	2 HPRT1
6 OR10C1	2 ANAPC4	3 HTR2C	4 SPRED2	1 CCR3	2 ATP5F1
6 ACHE	2 ATM	3 DARC	5 TBK1	1 PLA2G2D	2 NDUFC1
6 KCNB2	2 MTERF	3 PFKFB1	5 PIM1	1 SLCO1A2	2 PSEN2

6 SLC2A13
6 ALDH1A1
6 CHRM1
6 GAD1
6 DAO
6 CDH7
6 LEP
6 OPRM1
6 P2RY6
6 KCNH1
6 ADCY2
6 CALD1
6 XRCC3
6 SLC2A10
6 SLC22A3
6 OR10J1
6 NPY1R
6 KLKB1
6 KCNJ10
6 HSD17B3
6 GP9
6 GABRP
6 AVP
6 GABRA6
6 SLC24A4
6 ATP2A3
6 TGM2
6 CXCR1
6 F2RL3
6 VCAM1
6 PLCB2
6 SLC7A8
6 CHEK1
6 OR2J3
6 OR2W3
6 CCL2
6 OR2S2
6 OR5K1
6 HRH2
6 OR2C3
6 DEFB118
6 METTL2B
6 GLRA2
6 SSTR2
6 NOS2
6 ALB
6 KCNAB3
6 ATP9A
6 MTNR1A
6 ATP1A4
6 GLS
6 UGT2B15
6 CALCRL
6 OR51M1
6 CSF1R
6 ATP8B1
6 CACNG8
7 ABCC8
7 GNG12
7 PXN
7 KCNG3
7 SLC2A11
7 CD4
7 CHRM2
7 SLC2A5

E-GEOD-33836 (downregulate

1 EEF1A1
1 SOCS3
1 CDKN2C
1 NFATC1
1 TAP2
1 FCGR1A
1 IFNA2
1 CCL4
1 TNFSF9
1 IL5RA
1 PDGFRB
1 AKAP5
1 KIF20A
1 TAF1
1 KIR2DS3
1 IL17A

2 SUCLA2
2 ERCC4
2 POLH
2 ADHFE1
2 ZNF416
2 CTPS
2 RRM1
2 KLHL13
2 OR2AG2
2 RDX
2 ZW10
2 NSL1
2 NUPL2
3 NUP210
3 OR5D16
3 OR10H4
3 PPP2R5A
3 GTF2A1
3 UPRT
3 PPT1
3 FBXO4
3 FUT8
3 ABCG5
3 PIK3R1
3 CEP63
3 DPAGT1
3 MGAT4A
3 SYNJ1
3 HNRNPH1
3 TAF2
3 FANCC
3 ERCC6
3 EDEM2
3 SLC16A1
3 MTHFD2L
3 XRCC4
3 NUP93
3 TPP2
3 SLC22A3
3 FBXO11
3 GTF3C3
3 ZNF33B
3 FAR1
3 RFC5
3 KRAS
3 ZNF611
3 ERCC5
3 POLR1C
3 ZNF256
3 NARS2
3 MAN2A1
3 SOS1
3 OR9A2
3 RFC3
3 RAD54B
3 OCLN
3 PEX12
3 PIGX
3 BUB1B
3 PRIM2
3 NUP133
3 PSMD5
3 AMY2B
3 LOC643997
3 CASC5
3 USP8
3 ERCC8
3 POLR3B
3 PPP3CB
3 ZNF17
3 UBE4A
3 USP9X
3 IARS
3 TAF9B
3 PGAP1
3 SLC7A11
3 MED31
3 SNRPE
3 SLC25A32
3 TAF1B
3 POLA1
3 PNPT1
3 CEP135

3 DEFB130
3 GLP1R
3 MTMR7
3 MTNR1B
3 PRKCB
3 S1PR5
3 KCNK4
3 BMP2
3 CYP2C9
3 DEFB104A
3 RGS1
3 CACNA2D2
3 GNG7
3 RIMS1
3 GADD45G
3 PROKR1
3 ICAM1
3 PLA2G4E
3 PCOLCE
3 PFKFB3
3 CTF1
3 CACNB2
3 COL13A1
3 FGF18
3 ITGB2
3 BDKRB2
3 PTGFR
3 DRD2
3 ACHE
3 GNGT2
3 CXCL2
3 PENK
3 QRFP
3 PFKL
3 CCL3
3 IL24
3 CXCL9
3 PC
3 FGG
3 RARA
3 CALML5
3 COL9A2
3 CYP26C1
3 NFATC4
3 CYP2A7
3 HRH2
3 DES
3 COL5A1
3 CYP26A1
3 AVP
3 TNFRSF25
3 CHRNA7
3 GABRA6
3 PDE1B
3 RGS7
3 RPS6KA2
3 FOSL1
3 JUNB
3 GPR83
3 KCNJ3
3 MAP4K1
3 IFNA4
3 TAS1R2
3 VIP
3 IL18RAP
3 MUC15
3 GJD2
3 NFKBIB
3 SGCG
3 THY1
3 CCL2
3 COL2A1
3 GDF5
3 ATP1A4
3 CCL16
3 CD19
3 KCNS1
3 ATF4
3 SRF
3 CACNA1G
3 ADRA2B
3 IL6
3 COL4A4

5 TANK
5 NFKB1
5 SDC4
5 NFKBIA
5 PSMA6
5 MAP2K3
5 STAT5B
5 SLC1A3
5 BAK1
5 DOCK4
5 NOLC1
5 PSMA7
5 TNFAIP3
5 CSF2RB
5 REC8
5 NFKBIB
5 PSME1
5 CTLA4
5 TAP2
5 CD40
5 NLRC5
5 UBE2L6
5 TXN
5 TAP1
5 SOCS1
5 RIPK2
5 PSMB9
5 PSMA3
5 PML
5 MCL1
5 LTA
5 IRF1
5 GBP4
5 FLT3LG
5 GBP2
5 LMNB1
5 ACSL5
5 CDKN1A
5 AKT1S1
5 CYP3A7
5 CD80
5 PMAIP1
5 IFITM3
5 IRF7
5 TNIP2
5 TRAF4
5 ETS1
5 FAS
5 IL15RA
5 KIF18A
5 KPNA5
5 PSMD11
5 RPS6KA5
6 MED12
6 YWHAZ
6 PPP2R1A
6 DHX15
6 NUP153
6 FZD6
6 HMGA1
6 CHPF
6 NR1H2
6 SRSF4
6 PSMD5
6 ANAPC7
6 CDK2
6 MAP3K8
6 TNFRSF18
6 SOS1
6 HSP90AA1
6 MAFF
6 XAB2
6 ADRBK1
6 SRSF2
6 HNRNPF
6 BCL2L1
6 ZNF267
6 TNFSF14
6 PSMC4
6 IL2RB
6 PIK3R2
6 SYNE2
6 TAF4B

1 COL4A4
1 DSP
1 HSD17B2
1 IHH
1 KCNA5
1 SLC18A3
1 TNNI3
1 CYP19A1
1 TAS2R4
1 PDGFC
1 MMP8
1 FCGR2A
1 MASP1
1 RGS11
1 CHRNB3
1 PTGDS
1 CDH10
1 KCNJ15
1 RDH8
1 SCARB1
1 CD14
1 CTNNA3
1 CXCL12
1 HIST1H2BN
1 DKK1
1 SERPINB2
1 TRIO
1 NMU
1 IAPP
1 ADRA2A
1 CLEC1A
1 PFKFB1
1 GABRA1
1 CYBRD1
1 CCL27
1 COLQ
1 OR1A2
1 ABCC3
1 PTGFR
1 MMP2
1 FGG
1 AKR1C1
1 CCL25
1 PDE2A
1 FZD9
1 GLUL
1 WISP1
1 GPX2
1 ITGA8
1 PZP
1 SRD5A1
1 FGF5
1 CA1
1 RGS7
1 AQP9
1 CCL11
1 ITGB6
1 PLAUR
1 HSD17B3
1 CLDN15
1 SLC30A5
1 SLC4A1
1 GDF9
1 SFRP4
1 KCNB1
1 ITSN1
1 FCN2
1 HTR1E
1 SLC2A9
1 SLC17A7
1 ADAM20
1 SCN2A
1 HIST1H3H
1 GC
1 EDN1
1 ADCY1
1 CASR
1 CYP26A1
1 FSHR
1 PCOLCE2
1 A2M
1 KALRN
1 ADAMTS9

2 CHMP4A
2 NDUFA1
2 PSMA6
2 PTMA
2 BCAS2
2 SUGP1
2 RPL17
2 SMAD7
2 COX5A
2 NDUFC2
2 GSK3B
2 TRIM24
2 PSMF1
2 AP2S1
2 CD2BP2
2 SNRPD3
2 ARHGEF6
2 CTDP1
2 DYNC2H1
2 YARS2
2 NDUFAB1
2 TUBA3D
2 LSM3
2 ANKRA2
2 PNP
2 GPX1
2 UBE2L6
2 CD96
2 MSH3
2 CLASP1
2 HERC1
2 MAGOH
2 IFI6
2 ATP5I
2 RNF7
2 ATP5D
2 POLD4
2 PPIE
2 EIF3K
2 MGAT2
2 SART1
2 NUP43
2 NDUFB2
2 TBCA
2 LSM7
2 ADCY3
2 DAPP1
2 SRPRB
2 PSMB3
2 SEC11A
2 B4GALT2
2 CR1
2 RAP1A
2 SNRPG
2 DCTN3
2 PPP3CA
2 TNFRSF4
2 PDHB
2 C19orf40
2 SH2D2A
2 PPCS
2 RPS26
2 NAE1
2 OFD1
2 ATR
2 NUP133
2 VPS4B
2 ADSL
2 PSMC4
2 CKAP5
2 U2SURP
2 PIK3CA
2 UQCRC1
2 NDUFA10
2 ACAT2
2 DLD
2 PPAT
2 MIS18BP1
2 RAN
2 HSD17B10
2 CENPF
2 FAS
2 EIF2B5

1 CD72	3 RASA1	3 LTC4S	6 TNFRSF12A	1 AKT2	2 CD79A
1 CDC20	3 RARS2	3 PMM2	6 IL23R	1 CA14	2 PFDN2
1 HIST1H4G	3 ZNF567	3 CALML6	6 NFKB2	1 KCNC3	2 PSMB4
1 E2F5	3 JAK2	3 XCR1	6 FASN	1 MXRA5	2 RPS6KB2
1 PDCD1LG2	3 RASA2	3 RGR	6 ZFP37	1 MIP	2 XYLT2
1 ACTN4	3 CTH	3 CACNG5	6 ZFP28	1 MMP16	2 NUP62
1 IFNA4	3 PLK4	3 CSH1	6 YWHAE	1 GRIA4	2 UQCRQ
1 NPPA	3 OFD1	3 PDE6G	6 XRN2	1 IGF1R	2 IP6K2
1 VIP	3 AGK	3 MUC21	6 TP53	1 KCNJ1	2 TIMM17A
1 SPRY2	3 ZNF624	3 GP9	6 TERF2	1 ABCC2	2 EIF2B2
1 FASLG	3 IDH3A	3 PLAUR	6 STAT3	1 HAL	2 ETFDH
1 HIST3H3	3 UBR5	3 FUK	6 SNRPB	1 KCNAB1	2 GSTO1
1 CTF1	3 ACOX1	3 MPI	6 SLC3A2	1 G6PC2	2 POLR3K
1 ELANE	3 ZNF169	3 CD37	6 SF3B4	1 AVPR1B	2 IFI30
1 CD33	3 PIKFYVE	3 PROKR2	6 SERPINE1	1 BMP2	2 SAMM50
1 SPRED1	3 CAPN2	3 EPX	6 RDX	1 JUP	2 ARPC3
1 S100A2	3 EPRS	3 LHB	6 RBM22	1 PLXNB2	2 LCP2
1 LIN9	3 ALG8	3 FLT4	6 RASSF5	1 CACNA1F	2 INPPL1
1 IFNG	3 DET1	3 RGS20	6 RASGRP1	1 PKLR	2 PSMD8
1 IFNA1	3 LARS	3 HTR5A	6 PTK2	1 CLEC7A	2 SNRPF
1 ATP5A1	3 PDHX	3 ADORA2A	6 PTBP1	1 MYL2	2 SAE1
1 CDK5	3 REV3L	3 CXCL1	6 PSMD13	1 MYO10	2 NDUFS3
1 CCND1	3 RNMT	3 MMP17	6 PKM2	1 NR1D1	2 TAF11
1 S100B	3 CDC27	3 APOA1	6 KDM1A	1 RPS6KA6	2 HNRNPF
1 ACTA1	3 PHKB	3 MAP2K3	6 JAK1	1 SLC12A5	2 SF3B5
1 HIST1H4J	3 ALMS1	3 COL6A1	6 ITGA5	1 OR2S2	2 DYNC1H1
1 NDUFA3	3 FCGR1A	3 PYY	6 IRF9	1 IMPG1	2 ISG15
1 BDKRB2	3 ATIC	3 GRID1	6 IRAK2	1 BMP15	2 SEC61B
1 MME	3 CCAR1	3 PRKCG	6 IL2RG	1 CCL7	2 FBXW4
2 RPL31	3 RGNEF	3 PLOD1	6 IL18R1	1 SEMA6D	2 NADSYN1
2 TCEB1	3 CDK8	3 ELK1	6 IL10	1 MEP1B	2 ADAM17
2 ARPC1B	3 SAR1B	3 KCNC2	6 IARS	1 SERPINB5	2 GMPR2
2 S100A12	3 ZNF613	3 IL27RA	6 HSP90AB1	1 ADAMTS6	2 PPP1CA
2 CHP2	3 MAP3K7	3 APOC3	6 FURIN	1 GRIK3	2 GRAP2
2 C7	3 MLH1	3 IL22RA1	6 EP300	1 SEMA3G	2 MIS12
2 CTLA4	3 E2F6	3 TBX21	6 CSK	1 PRKG2	2 ACAA1
2 NUP35	3 NEDD1	3 CYP4A22	6 CSF1	1 GALR2	2 TUBB2A
2 ZNRD1	3 PHKA1	3 WNT16	6 CPSF7	1 AQP4	2 NDUFA7
2 TAF12	3 ZNF383	3 CCL22	6 CD7	1 CYP2A7	2 PSMC3
2 MAFF	3 NUP43	3 FGF23	6 CCND3	1 CA6	2 CCNH
2 HRAS	3 AKR1B1	3 BMP1	6 CCL1	1 KCNS3	2 TUBA4A
2 SRP19	3 KLC1	3 CAMK2B	6 CASP2	1 NR2E1	2 NCF4
2 ARPC3	3 TAF11	3 CYP11B1	6 ACTN4	1 HIST1H4D	2 NUDT21
2 CD3D	3 MDM4	3 BCAR1	6 ADAR	1 MAOB	2 RNASEH2A
2 RBL2	3 MYD88	3 KCNJ11	7 DNM1L	1 MAS1	2 UCKL1
2 EIF3D	3 SLC38A1	3 TNNT2	7 B2M	1 SLC28A1	2 SYCP2
2 TAF10	3 ALG13	3 CACNA1F	7 GCK	1 GPLD1	2 COX17
2 ATP5J	3 NMT2	3 FSHB	7 NDEL1	1 CACNG3	2 DPM3
2 SF3B5	3 CSTF2	3 RDH16	7 SLU7	1 IFNA4	2 COX6B1
2 HNRNPA3	3 PITPNB	3 GABRG3	7 H2AFX	1 PLA2G3	2 COX8A
2 COX6A1	3 ANAPC7	3 KCNB2	7 HIST2H2AB	1 ABCC6	2 DCLRE1C
2 ATP5L	3 TRRAP	3 COL3A1	7 HIST2H4B	1 RHO	2 AUH
2 PTPN7	3 YARS	3 CTLA4	7 POLR3G	1 LRP6	2 NDUFA13
2 IL22	3 POLR1B	3 MYLK3	7 HNRNPH2	1 BAIAP2	2 CASP7
2 OGDH	3 FBXO3	3 VAMP2	7 B9D2	1 LAMB3	2 UQCR11
2 NCR3	3 UBE2J1	3 CACNA1S	7 ADAM17	1 EPB41L3	2 CYBA
2 PQBP1	3 H3F3B	3 CCL3L1	7 SHC2	1 MBOAT2	2 NDUFA4
2 RIT2	3 PIK3C2A	3 FOXP3	7 WHSC2	1 CLDN7	2 POLH
2 TCEB3	3 SMARCA5	3 KCNQ5	7 IKBKB	1 KCNN2	2 RBX1
2 POLR2A	3 GTF2I	3 CCL17	7 ZNF583	1 CPN2	2 NDUFB8
2 PRPF6	3 RRM2B	3 GPR17	7 BCAR1	1 SULF1	2 SMN2
2 RAET1E	3 MAP2K4	3 IFNA8	7 MAVS	1 TLE1	2 SHFM1
2 POLE	3 SMUG1	3 ADAMTS2	7 PRKDC	1 ASH1L	2 LSM2
2 FOXO4	3 DUT	3 KCNQ2	7 ZNF441	1 LGALS13	2 PIN1
2 RBM8A	3 UGP2	4 ITGB7	7 NUPL2	1 SGCG	2 CHUK
2 TNPO1	3 TAF12	4 CXCR5	7 TRA2A	1 SERPINB4	2 DROSHA
2 VAV2	3 UBR1	4 NOS3	7 SMAD4	1 TRH	2 CD72
2 PRKCZ	3 WDR77	4 POMC	7 TGIF1	1 AGTR2	2 NDUFS8
2 RBM17	3 CCT2	4 VAV1	7 PLD2	1 TGFB2	2 DUSP4
2 CALR	3 SLC22A15	4 CHRM1	7 PSMD1	1 MYH7B	2 EXOSC8
2 IFNA16	3 NR5A2	4 FGF4	7 CENPI	1 AGT	2 HLA.DQA2
2 CD28	3 ZNF184	4 GRK4	7 PPIH	1 S100G	2 BCL2A1
2 TGFB1	3 AHCTF1	4 FPR3	7 BIRC2	1 ANGPTL2	2 CTSS
2 RPS21	3 AP4B1	4 CRHR2	7 U2AF1	1 THRB	2 NDUFS1
2 POLD3	3 HSD17B12	4 MS4A2	7 CREBBP	1 HIST1H2AG	2 TNRC6B
2 FAU	3 SEC61A2	4 VAV3	7 PTK2B	1 PTPRF	2 UBE2E3
2 RPS20	3 SEC23A	4 COL1A2	7 TCERG1	1 SST	2 BATF3
2 RPL4	3 PARP2	4 DEFB135	7 SNAPC3	1 MFAP5	2 TNFSF10
2 SF3B4	3 XIAP	4 PFKFB4	7 DHX9	1 LEPREL1	2 RASA1
2 RPS2	3 NCOA3	4 KCNJ10	7 DDX3Y	1 HTR3B	2 RASGRP3
2 RPL28	3 CDK6	4 FMO3	7 TNFRSF25	1 KERA	2 CSGALNACT2
2 RPL39	3 SLC30A7	4 DMP1	7 DNA2	1 JAM2	2 PSMD13
2 HIST1H4H	3 ZNF230	4 MYBPC1	7 FADD	1 GLRA1	2 OSM

2 NUP50	3 RNF34	4 PTGER3	7 PSMA1	1 OR1A1	2 CDKN1A
2 XCR1	3 TEAD1	4 KLKB1	7 UGCG	1 SCN1B	2 EIF1AX
2 MEF2C	3 NFX1	4 COX7A1	7 NEDD1	1 TNFSF18	2 NUPL1
2 CYR61	3 GNPTAB	4 CYP3A5	7 ZNF569	1 CYP2E1	2 SKP2
2 TNFSF8	3 PREB	4 CXCR7	7 CAB39	1 RARA	2 FANCG
2 TNFSF11	3 PDP2	4 GPX5	7 SLC38A2	1 BRS3	2 IFI35
2 SDC1	3 PSMB2	4 FZD7	7 RIPK1	1 GNRHR	2 TNFRSF14
2 IDUA	3 SEC31A	4 GRM1	7 U2AF2	1 CHRM5	2 HLA.DPB1
2 EIF2B5	3 ANAPC5	4 HLA.DPB1	7 PYGM	1 CYP26B1	2 PSMD3
2 GATA4	3 SLC9A9	4 FGF16	7 ZNF20	1 NTF3	2 PRKCZ
2 RPL36	3 PIAS2	4 IFNB1	7 EXOG	1 CAP2	2 TRAF3
2 RPS12	3 ZNF544	4 PCK1	7 CREB1	1 FMO4	2 MAD1L1
2 RPL19	3 CUL1	4 CCBP2	7 CCNE1	1 ATP10B	2 SRPR
2 PAK7	3 ZNF426	4 EDNRB	7 TAF4	1 CLDN17	2 PSMB10
2 EHD4	3 DLD	4 TNFSF18	7 FOXO1	1 F2RL3	2 USP18
2 IKBKAP	3 NPC1	4 CCL23	7 HNRNPM	1 GABRE	2 ETFB
2 TAF4	3 SLC9A6	4 P2RY8	7 ZNF347	1 VIPR1	2 ITPR1
2 RPL24	3 RHOA	4 STX1B	7 SELP	1 GUCY2F	2 PTPRK
2 RPL23	3 ZNF431	4 CHRNB3	7 CD28	1 DOCK1	2 RPA3
2 MAP3K2	3 ZNF37A	4 CXCL5	7 RPS6KB1	1 LGALSL	2 FXN
2 EEF2	3 TRIM32	4 DEFB136	7 HIST1H4F	1 LEFTY1	2 MBD4
2 AGT	3 CUL2	4 CD1A	7 HIST1H4I	1 MYL1	2 POMP
2 CXCL2	3 SLC44A1	4 KL	7 ZNF442	1 GNAT3	2 BIRC3
2 MAP3K6	3 TEX15	4 CD38	7 SLC43A2	1 CBLN1	2 CD79B
2 CAV1	3 OR1J2	4 GPR65	7 SDCCAG8	1 COL10A1	2 UBE2S
2 HLA.DQA1	3 PIK3C2G	4 FGB	7 TAF1A	1 CNTN1	2 DFFB
2 RPL22	3 PAPSS2	4 IFNA21	7 RB1	1 KCNJ2	2 JUN
2 RPL23A	3 SLC5A7	4 TLR1	7 ADA	1 F2	2 HIST1H4C
2 IFNA21	3 OR9K2	4 TLR2	7 ZNF582	1 KCNMB3	2 CREM
2 KIF11	3 ZNF615	4 CYP2C19	7 ZNF101	1 FGA	2 UBE2V2
2 DKK1	3 SLC25A4	4 RHCG	7 TCP1	1 GJB3	2 CSGALNACT1
2 CD19	3 UPF3B	4 CYP24A1	7 NUP205	1 PROL1	2 TJP2
2 CXCL3	3 OR52E8	4 MUC19	7 RFFL	1 CDH7	2 CHMP2A
2 ACTB	3 PIK3CA	4 PDE6A	7 ATF2	1 ABCC9	2 ICOS
2 NFATC3	3 PDHB	4 AWAT2	7 ZNF567	1 TIMP2	2 KIF2C
2 SF3A2	3 MED17	4 FGA	7 PSMC6	1 ESRRG	2 KPNA2
2 FBXW7	3 OR4M2	4 KCNA2	7 SNAPC1	1 HIST2H2BE	2 DBF4
2 POLR2B	3 CDC16	4 BST1	7 ZNF224	1 PRKG1	2 MAVS
2 RBX1	3 RAD17	4 IL5	7 KAT2A	1 C5	2 POLR2K
2 RIPK3	3 WWP1	4 FSHR	7 NUP107	1 VWF	2 CDK8
2 PFDN2	3 MED7	4 DUSP8	7 PCF11	1 CYP2C18	2 RFXANK
2 CSNK2B	3 ZNF382	4 MCHR2	7 ZNF445	1 SERPIND1	2 XPA
2 THOC4	3 UGT2B4	4 CXCL14	7 ZNF552	1 ADAM29	2 BLNK
2 CD55	3 ZNF415	4 KCNA7	7 GSK3A	1 ADCY6	2 CASP4
2 MAL	3 FIG4	4 PF4	7 USP39	1 CNTN2	2 HERC2
2 HNRNPH2	3 PDC	4 CYP4A11	7 ZNF708	1 PLCB1	2 IRF8
2 ETF1	3 LIPF	4 ICAM4	7 ZNF430	1 PMCH	2 CDC20
2 HIST1H2BK	3 ZFP112	4 NCAM1	7 ZNF417	1 S100B	2 TIMM13
2 LCP2	3 PRKDC	4 MMP10	7 ZNF354A	1 UGT2B28	2 CKS1B
2 CCT5	3 ATP6V0A1	4 ADH1B	7 TDP2	1 FMO1	2 SYNE2
2 C12orf5	3 BLMH	4 GPX2	7 RUNX1	1 TAAR5	2 DHFR
2 RPA3	3 L2HGDH	4 NTF4	7 RAD21	1 PARD6A	2 GBP2
2 POLR3GL	3 PPP2R5C	4 CPE	7 PRKAG2	1 PLA2G2E	2 REV1
2 SEC11A	3 SLC7A6	4 PTGIS	7 MED26	1 UNC5B	2 FUS
2 EIF3I	3 AP3M1	4 SLC1A2	7 MAPK8	1 EDNRA	2 MAP4K3
2 SHC1	3 OR2A4	4 IL2RG	7 IL4	1 GRIP2	2 POLR2I
2 HLA.A	3 CPSF3	4 NTS	7 FBXO5	1 WNT2B	2 BAG4
2 EIF4G2	3 BTRC	4 CYP2E1	7 EXOC6	1 CYP2B6	2 TCEB1
2 RPL8	3 RAD1	4 CGA	7 CHERP	1 WASF3	2 CHMP3
2 SF3A3	3 ZNF619	4 HTR2A	7 CEP135	1 NR4A3	2 CCL17
2 LSM7	3 LY96	4 GLRA3	7 CENPT	1 F7	2 HDAC4
2 RPL29	3 ZNF510	4 MMP7	7 CCT4	1 GJD2	2 CCL22
2 MAPKAPK3	3 MTM1	4 GRAP2	7 CDK8	1 OPRK1	2 LONP2
2 FOXO3	3 PIK3R4	4 MUC12	7 CLIP1	1 IL37	2 GTF2A2
2 EEF1B2	3 SEC24D	4 AGTR2	7 EIF4E3	1 CYP17A1	2 PCCB
2 RPL7A	3 UBE4B	4 INHBC	7 CXCL2	1 HTRA1	2 REV3L
2 BCL3	3 RCHY1	4 CR1	7 NUP43	1 TFPI	2 HNRNPH2
2 MARCKSL1	3 UBE2A	4 CXCL13	7 EGLN1	1 SLC6A15	2 PTPN2
2 PRDX1	3 DOCK2	4 UGT2B28	7 PRKACB	1 CYP7B1	2 VBP1
2 EIF4H	3 PDPR	4 ADH4	7 DOCK5	1 NTS	2 BANF1
2 CEBPZ	3 EEF1E1	4 NPY5R	7 PLD1	1 LTF	2 IL7
2 ITPKB	3 ALG14	4 CYP2B6	7 HIST1H2AJ	1 PPBP	2 SNAPC5
2 RPL3L	3 ZNF540	4 KDR	7 STX1A	1 CCKBR	2 DCTN2
2 SLC25A5	3 OR4K15	4 FBP1	7 HK2	1 LPAR1	2 EBI3
2 SMARCA4	3 PRKACB	4 NPFFR2	7 PDE1B	1 RORB	2 C1D
2 NCR2	3 ASB17	4 RPE65	8 CYCS	1 CYP2J2	2 LY96
2 ATM	3 PDK3	4 CYP3A4	8 HIST1H3F	1 CSH2	2 ISG20
2 CHERP	3 UBA5	4 IL21	8 CAMKK1	1 SLC1A7	2 TBCE
2 PTPRC	3 EIF4E3	4 TRPC6	8 CREB5	2 VEGFC	2 NT5C2
2 HNRNPA2B1	3 RNF7	4 CACNA1E	8 CDKN2B	2 GRIA2	2 UGCG
2 EXOSC2	3 B4GALT6	4 DEFB128	8 HCK	2 HIST1H2AI	2 PAPOLA
2 SRSF2	3 RBL2	4 MMP13	8 GTF2E2	2 FPR1	2 TOMM22
2 SMAD3	3 CENPJ	4 ADORA3	8 KIF5B	2 SLC16A8	2 CYFIP2

2 EIF4A2
2 IL4
2 ELF1
2 TNFRSF10A
2 ATP5H
2 MAFK
2 U2AF1
2 IL6R
2 MAP3K14
2 EIF5B
2 IL27RA
2 TGIF2
2 RPL3
2 OAZ1
2 TUBA1B
2 TUBA3D
2 STRAP
2 HMGA1
2 ADAM17
2 BAX
2 RNMT
2 DDX5
2 NFAT5
2 TCP1
2 PSMC1
2 STAM
2 YWHAB
2 ACTN1
2 SNRPG
2 RASSF5
2 PIK3CA
2 ITK
2 SNRPA
2 KLC1
2 RIT1
2 BRAF
2 PUF60
2 COX6C
2 PRPF8
2 EIF1
2 PSMB5
2 CAMK4
2 HSP90AA1
2 ITCH
2 CYFIP2
2 UQCRH
2 EIF3H
2 RPN1
3 IRF7
3 UCP2
3 HIST1H3H
3 IRF1
3 ARF6
3 ICOSLG
3 KIR2DL4
3 SRSF6
3 PAK2
3 ACVR1
3 PSMB3
3 NDUFA8
3 GPC6
3 PDHB
3 SRP14
3 ARID3A
3 IL23A
3 SP1
3 GTF2B
3 STIM1
3 EIF4A3
3 AFP
3 PHF5A
3 IFNGR2
3 GPC2
3 MCL1
3 SRSF3
3 HLX
3 HCK
3 CDC25A
3 FOXP3
3 PPP2CB
3 TNFRSF12A
3 RPL13
3 TAB2
3 IARS2
3 CENPC1
3 PPM1D
3 RBMX
3 YES1
3 ZNF649
3 UBR2
3 ALG2
3 APAF1
3 NUP205
3 GBE1
3 TRAF6
3 HECTD3
3 ENTPD4
3 SLC9A2
3 ZNF26
3 EVI5
3 SLC35D1
3 MED8
3 ZNF254
3 TBP
3 MDM2
3 NFIB
3 PI3
3 TARSL2
3 AP1G1
3 HACL1
3 ESR2
3 ZNF300
3 NUP155
3 CTSO
3 DHX9
3 LNPEP
3 SACM1L
3 ZNF350
3 CDC25C
3 HNRNPR
3 SLC22A5
3 TFDP1
3 ATP6V0A2
3 CHEK1
3 EIF2S1
3 NF1
3 BIRC6
3 CALM2
3 PIP4K2A
3 OR2T6
3 SETD7
3 CTNNB1
3 OR2A7
3 OR5AS1
3 ALDH9A1
3 MCM6
3 RANBP2
3 ACSL1
3 TAF13
3 NUP107
3 GARS
3 SMC3
3 FANCI
3 CPSF2
3 OR9I1
3 SLC1A1
3 GTF2E2
3 PSMA4
3 GTF2H1
3 LCLAT1
3 FARSB
3 GSK3B
3 MED4
3 RNF4
3 ERAP1
3 PIK3C3
3 POLR2D
3 CEP192
3 XYLB
3 SLC35A3
3 ZNF432
3 SDCCAG8
3 CDCA8
3 KLHL9
3 OCRL
3 PPP1CC
4 GIP
4 KCNA4
4 DEFB115
4 SLC6A14
4 MYH8
4 ALDOB
4 DEFB114
8 PRPF18
8 SC5DL
8 TRAF3
8 RAPGEF1
8 SMPD2
8 CSNK1D
8 MCM6
8 SORBS1
8 CHST11
8 HLX
8 CAMKK2
8 CDK1
8 SGOL1
8 CD2BP2
8 PIK3AP1
8 PPP2CA
8 AP3M1
8 PRKAR1A
8 NUP188
8 PGK1
8 CASP8
8 JUNB
8 PSME2
8 MDM2
8 SNRNP40
8 CCNA2
8 RPS6KB2
8 NCOA4
8 CAB39L
8 CSF2RA
9 MAP3K5
9 SLC38A5
9 PI3
9 LDHA
9 SLC7A5
9 GZMB
9 TIAM2
9 TRAF1
9 ITGA1
9 PTPN2
9 FLOT1
9 SOCS3
9 IFITM1
9 ISG20
9 IL18RAP
9 IL12RB2
9 SLC1A1
9 ZNF254
9 XAF1
9 TNFRSF6B
9 PTGS2
9 PLAT
9 MAPK11
9 IL6
9 IL2RA
9 IL24
9 IL19
9 IFNG
9 DCN
9 CCL19
9 CRLF2
9 CFB
9 ITGA3

E-GEOD-57418 (Downregulat

1 CD9
1 FOS
1 LGALS3
1 PSAP
1 SDC3
1 LIPA
1 APOE
1 IL1R2
1 RPS6KA2
1 FN1
1 PPARG
1 ARHGDIG
1 SNCA
1 APOC2
1 VWF
1 HLA.DRB4
1 SMO
1 A2M
2 PLA2G2F
2 SLC24A2
2 DGKI
2 NRP2
2 SERPINA1
2 ALDH1A1
2 GLS2
2 ATP1A2
2 PARM1
2 IL3
2 RHOB
2 CTNND1
2 MUC16
2 MUC5B
2 CREBBP
2 KLK2
2 PTPN1
2 C9
2 SOX17
2 S100A12
2 CDH3
2 TNR
2 TNC
2 OPRL1
2 PLG
2 PSPN
2 TLN2
2 ADRBK1
2 ATP2B3
2 AQP6
2 CHRD
2 GAS1
2 MAPK10
2 CYP24A1
2 GLP2R
2 STARD5
2 TMOD1
2 PRKAG2
2 SSTR4
2 FZD2
2 SLC1A2
2 VDR
2 C7
2 SLC29A3
2 GRM7
2 ABCG5
2 SLIT1
2 CACNA1S
2 ITGA10
2 KCNH6
2 HIST1H2BE
2 HGFAC
2 HIST1H2BG
2 GJB5
2 EDN2
2 FTH1
2 PLCD1
2 COL7A1
2 CACNB2
2 GRM2
2 THBD
2 NR1I3
2 WNT11
2 EHMT2
2 MMP27
2 ABCA7
2 ATP2A1
2 SLC3A2
2 CELA3B
2 HIST2H2AA4
2 FGF7
2 CRK
2 ACE2
2 GDNF
2 SLC38A3
2 SLC13A2
2 SERPINB13
2 P2RX1
2 BTRC
2 HSD17B1
2 LGALS4
2 SLC27A6
2 LAMB4
2 UQCRC2
2 NDUFB1
2 ACP5
2 L2HGDH
2 TIMM8B
2 SNRPE
2 ACADM
2 SSB
2 NCBP1
2 CTNNBL1
2 GALNT1
2 RPL26L1
2 PLCB3
2 PSMA5
2 TERF1
2 TLR1
2 NARS2
2 AHCTF1
2 NDUFS4
3 ACAA2
3 CSTF2
3 QARS
3 ERCC2
3 MLST8
3 PEBP1
3 IL13
3 RAC3
3 BCR
3 SRP72
3 NFATC3
3 CREB3L2
3 RPS6KA5
3 RHOC
3 IRAK1
3 RPN1
3 DUSP6
3 TIMM10
3 CUL4A
3 HTRA2
3 C17orf70
3 DDX42
3 CD3G
3 SEC61G
3 HNRNPUL1
3 SSR2
3 XRCC1
3 FBXW7
3 PDIA3
4 MTX1
4 NUP85
4 CSF2RB
4 MAPKAPK3
4 BMPR1A
4 DDOST
4 HLA.E
4 GMPPB
4 MAP3K8
4 ITGA1
4 NOD2
4 PPP1R14B
4 TYMP
4 CLIP1
4 DCTN6
4 PTPN7
4 SH2B3
4 ERCC3
4 PSME2
4 PPA2
4 SSBP1
4 PSMB9
4 UNG
4 DTYMK
4 EXOSC10
4 NDUFS6
4 ALG5
4 GRK6
4 PMM2
4 BARD1
4 PPCDC
4 EGR2
4 RGS1
4 CCL3L3
4 IL2RA

3 RAB5A	3 ZNF200	1 LPL	2 CALM1	4 TNF
3 ITGA2B	3 AGPAT5	1 CD1C	2 SPARC	4 STAM
3 NFKBIB	3 RACGAP1	1 CHIT1	2 F3	4 CHST11
3 TNFAIP3	3 MAN1A1	1 IL1R1	2 GABRB3	4 DDB2
3 CREB1	3 CCT8	1 CD68	2 GSTM5	4 MX1
3 SUCLG1	3 DARS2	1 HLA.DMB	2 PDE8B	4 GALNT6
3 HNRNPR	3 SF3A3	2 CD40LG	2 SSTR1	4 GZMA
3 KLRK1	3 MAPK8	2 MADCAM1	2 HNF4G	4 SF3B3
3 SOS2	3 NUDT21	2 ARPC4	2 RGS6	4 SRSF2
3 HMGB1	3 TARS	2 ARHGEF11	2 CLDN8	4 IARS2
3 ANAPC7	3 CENPL	2 SUCLG2	2 CDH12	4 UBE3C
3 PCNA	3 CP	2 PCK2	2 COL6A1	4 BLM
3 ATP5B	3 SLC35A1	2 ACAT1	2 COL5A1	4 MIS18A
3 CDK7	3 HNRNPA2B1	2 SMPD3	2 FPR2	4 GMNN
3 RPN2	3 SLC35B2	2 MEF2A	2 PPP2R1B	4 POLA2
3 EXOSC9	3 NCBP1	2 PIK3R1	2 THBS2	4 CDC6
3 TRAF2	3 RBBP7	2 P2RY1	2 ANXA5	4 IFNG
3 NFKBIL1	3 WARS	2 UNC93B1	2 EMR2	4 EIF4G3
3 H3F3B	3 ZNF140	2 VAMP8	2 CHRNA4	4 CDK2
3 NME1	3 NNT	2 PCCB	2 FGFR3	4 FOXM1
3 CASP10	3 PSMC6	2 MBOAT1	2 PTCH1	4 AK4
3 IL2RA	3 PPIL2	2 MCEE	2 HTR2A	4 DSN1
3 PSMD14	3 RUVBL1	2 PLA2G12A	2 MYBPC2	4 PSMD1
3 JMJD1C	3 ZNF266	2 ABAT	2 ST8SIA2	4 ANAPC1
3 SNRPB	3 CCNH	2 GRIN2C	2 COL14A1	4 HLA.DMA
3 CSNK1A1	3 PARP4	2 COX8A	2 FGFBP1	4 SLC25A16
3 SRF	3 FAR2	2 COX6C	2 GFRA4	4 WBP11
3 SMPD1	3 NT5E	2 NET1	2 TGFB3	4 JUNB
3 UBE2N	3 PTS	2 PRR5.ARHGAP8	2 TGM3	4 ZBTB32
3 CSF3R	3 UBE3B	2 PARK7	2 IL11	4 TUBB3
3 EIF3E	3 ALG10	2 SDHD	2 OR2J2	4 CDC37
3 HDAC1	3 SEH1L	2 NDUFA8	2 FGF17	4 CHMP6
3 EXOSC1	3 PEX1	2 MDH1	2 GRIK5	4 SMC1A
3 SF3B2	3 PIGU	2 SDHB	2 ATP10A	4 MYBL2
3 UBE2A	3 KPNA2	2 ATP5I	2 CDK5R1	4 FUT8
3 POLR3G	3 XPO1	2 STX10	2 EGFR	4 CDT1
3 RBM25	3 NCK2	2 DGKH	2 KCNA10	4 PFAS
3 PRKCH	3 SF3B3	2 TPM1	2 ADRA1B	4 CDC45
3 TAB3	3 PTK2	2 NDUFA2	2 MMP15	4 TK1
3 RFWD2	3 SNRNP40	2 PDHA1	2 GNAZ	4 MCM6
3 PSMD7	3 PSMD1	2 ABCG1	2 BDKRB2	4 POLR2E
3 NFKB2	3 CDC40	2 ACADM	2 GBA3	4 VDAC1
3 DCP1A	3 EIF3A	2 ESRRA	2 GHRH	4 CBX5
3 FLNB	3 RB1	2 NDUFS3	2 NRTN	4 MEF2A
3 WAS	3 ZFP90	2 AP1B1	2 SPON2	4 RFC5
3 POLR3H	3 HERC5	2 P4HB	2 SYT5	4 RPA1
3 PSMB4	3 ZNF33A	2 PDK3	2 GSK3A	4 XRCC4
3 RASGRP3	3 MARS2	2 ARSG	2 ENAH	4 SEC63
3 KIF23	3 HIST1H2BK	2 AP2A2	2 NRG1	4 CCNE1
3 PPP2R3B	3 KPNB1	2 CTSA	2 PCSK1	4 TOPBP1
3 TGFBR2	3 TAF1A	2 PLCB1	2 CHRNA9	4 NUP37
3 SLC16A3	3 PSMA2	2 RALBP1	2 ATP9A	4 EP300
3 MAP3K12	3 TUBA4A	2 HLA.DOA	2 GRM3	4 FBXO5
3 IRS1	3 ATG7	2 LOC643454	2 HGF	4 GTF2H5
3 ANAPC11	3 TUBGCP3	2 NDUFB10	2 F13B	4 IL1R2
3 CCL23	3 CCNT1	2 CASP1	2 ITGA9	4 RRM1
3 UBA1	3 FBXW11	2 SUCLG1	2 MDK	4 ANAPC10
3 CCT7	3 SMURF2	2 ATP5J	2 PLOD3	4 MSH2
3 PPM1A	3 CEP57	2 MAPK13	2 HIST1H3E	4 TPK1
3 IL2RG	3 SLC38A2	2 PPP3CA	2 COL4A3	4 HJURP
3 PSMC5	3 PIGS	2 GGA2	2 GDF10	4 RSF1
3 SRSF7	3 PSMD14	2 PNPLA8	2 CXCL1	4 SMAD2
3 PPP3R2	3 E2F3	2 STX8	2 BMP6	4 SNRPB
3 RPL10	3 NUP88	2 C7	2 GRIN2D	4 SLC19A2
3 RHOA	3 PLK1	2 CAPN2	2 NR2F1	4 TAP1
3 POLR2E	3 PPP1R13B	2 GNAI3	2 WNT10B	4 NFKBIA
3 MAP3K11	3 ERCC3	2 OR7E24	2 DMD	4 LIG1
3 FYB	3 SDHA	2 PMM2	2 INSL6	4 MCM5
3 HPSE	3 ZNF621	2 RAB5C	2 MRAS	4 KIF3B
3 USP9X	3 GRB2	2 ARHGEF12	2 CLTC	4 RNASEH1
3 SRSF5	3 TUBA1B	2 TXNDC5	2 PRKACA	4 PSMB7
3 PSMC4	3 CDKN1B	2 CACNG5	2 PLA2G4A	4 TNFRSF1B
3 PSMA4	3 UBE3C	2 CHRNA7	2 CDH4	4 PRKX
3 POLR2J	3 HIST1H4H	2 DCT	2 COL8A2	4 CDC7
3 LCK	3 MAPK1	2 AP1G1	2 GJA4	4 COQ2
3 KIFAP3	3 SLC36A1	2 PRSS2	2 PTGER1	4 PIGB
3 AZIN1	3 MIS12	2 NDUFV2	2 ACAN	4 MGMT
3 EIF1AX	3 PSME4	2 NDUFS6	2 HSD3B2	4 TFDP1
3 XRCC5	3 RAN	2 NDUFS1	2 PLXND1	4 TUBGCP5
3 WBP11	3 EIF4G3	2 NDUFA9	2 TNNC1	4 UPF3A
3 SOCS4	3 POLR2G	2 NDUFA7	2 CDX2	4 EGR3
3 RPS15	3 RAE1	2 NDUFA11	2 GRIA3	4 TFRC
3 NDUFB6	3 AIMP1	2 HIBCH	2 INHBA	4 IFT57

3 ICAM2	3 EIF1AX	2 GALK2	2 KCNH2	4 KIF23
3 CCT3	3 SDHD	2 FZD9	2 GRIN2A	4 SPC25
3 BUB3	3 MAX	2 ATP1B3	2 GPR39	4 SMC2
3 CCT2	3 TAF5	2 ATP5G1	2 CHRND	4 TUBGCP3
3 ORC2	3 PIGQ	2 IDUA	2 PTPRB	4 GBP1
3 CCNH	3 VCL	2 VDAC2	2 TSHB	4 SRSF10
3 SKP1	3 OR5A2	2 UQCRC2	2 THSD4	4 ECT2
3 EXOSC8	3 UBE2G1	2 TBC1D8B	2 ARHGEF17	4 NCAPG
3 PPIL1	3 NCBP2	2 NDUFB2	2 STS	4 DLGAP5
3 NUP93	3 RBL1	2 MAPK7	2 BMP8A	4 KIF15
3 PDP1	3 RBPJ	2 KCNC4	2 SDC3	4 HLA.DRA
3 RAP1A	3 PIGT	2 GPX2	2 SLC6A2	4 NDC80
3 GADD45A	3 TCP1	2 D2HGDH	2 KCNA1	4 SKA1
3 NFKB1	3 TUBA1A	2 CLTA	2 FSTL1	4 PRPS2
3 AP2M1	3 TUBGCP5	2 ABCG2	2 HNF4A	4 LTA
3 PSMD4	3 QARS	2 CHRM3	2 CDH15	4 GINS2
3 TH1L	3 LMAN1	2 NDUFA5	2 MFAP3	4 CXCR3
3 CUL1	3 ZNF154	2 ADCY3	2 TNFAIP6	4 KIF11
3 RAN	3 METTL6	2 APBB1	2 RHAG	4 FANCI
3 POLR1C	3 PSMA3	2 ATP5G2	2 PCOLCE	4 BRIP1
3 H2AFV	3 MANEA	2 COX5B	2 SEMA6A	4 FEN1
3 HNRNPK	3 STAT1	2 COX7A2	2 CHRNB2	4 ITGB3BP
3 NDUFA6	3 FH	2 NDUFB9	2 ECM1	4 TTK
3 PPP2R2A	3 SLC4A7	2 VDAC3	2 SERPINC1	4 POLE2
3 MAP3K1	3 NUP37	2 ATP5L	2 CAV3	4 UCK2
3 ARF4	3 METTL3	2 NDUFS5	2 CCL16	4 BUB1B
3 RPS6KB1	3 SMC2	2 UQCRFS1	2 GJC1	4 PLK4
3 CDC26	3 DHFR	2 SLC25A5	2 AREG	4 IL18RAP
3 PSMB1	3 ZNF551	2 POLR2L	2 C8A	4 MCM7
3 TUBB3	3 CSNK1G3	2 GGCT	2 CD46	4 ZWINT
3 NDUFB9	3 MBD2	2 NDUFA4	2 GP1BA	4 TIPIN
3 PPP2CA	3 TRIM37	2 ATP6V1G1	2 PRSS2	4 IL18R1
3 NCOR2	3 ABCC4	2 GSTP1	2 GAST	4 CDK1
3 ORAI1	3 SUPT16H	2 HADHB	2 PRKCG	4 RRM2
3 NDUFAB1	3 RFC1	2 KCNG4	2 AGPAT1	4 CCNE2
3 ZMAT2	3 SKA1	2 GUSB	2 CRLF1	4 CHEK1
3 CWC15	3 NDC80	2 IFI30	2 COL17A1	4 CHSY1
3 RAB11A	3 TAS2R5	2 ATP5O	2 ELK1	4 FANCL
3 SMNDC1	3 RAP1B	2 NDUFA13	2 FGF9	4 RFC2
3 RAD23B	3 TAF9	2 APAF1	2 ARSJ	4 MAD2L1
3 PARP1	3 ITCH	2 ARPC3	2 FGF18	4 SMURF2
3 PSMA6	3 RAD51C	2 ATP5H	2 CHRNA6	4 TCERG1
3 EIF2S1	3 POLR3F	2 UQCRB	2 TNFSF8	4 KNTC1
3 CD48	3 CCT6A	2 RAC1	2 CYP4B1	4 RBL1
3 SNRPD3	3 LAMP3	2 ATP5C1	2 C4B	4 GINS1
3 ROCK1	3 ELOVL6	2 NDUFC1	2 ADH6	4 GZMB
3 UQCRFS1	3 PPP2R1B	2 UBE2L3	2 TINAGL1	4 XCL2
3 POLR1B	3 CUL5	2 NDUFB1	2 SLC5A2	4 CENPN
3 EIF4G1	3 SLC25A16	2 COX6B1	2 HTR1D	4 BCOR
3 TAOK1	3 PAPOLA	2 AUH	2 LEFTY2	4 PRC1
3 VBP1	3 SLC35B4	2 RRM1	2 DPT	4 AURKB
3 SRSF9	3 CCNC	2 ATP5F1	2 FSTL3	4 BRCA1
3 PDHA1	3 NUP50	2 POLR2E	2 LPCAT4	4 MCM2
3 RAC2	3 MTFMT	2 DLAT	2 PROS1	4 APITD1
3 SDHD	3 PALB2	2 ETFA	2 APOA1	4 ADK
3 CLP1	3 ZNF192	2 SNX5	2 ARHGAP35	4 ZWILCH
3 E2F3	3 AHCYL1	2 NDUFB5	2 BMP4	4 CCNA2
3 SRSF8	3 BARD1	2 PLDN	2 OR7C2	4 OAS3
3 PPP2R1A	3 AKAP9	2 AP3S1	2 SLC6A6	4 ORC1
3 PSMD5	3 AP4E1	2 MCCC1	2 TAC3	4 DUT
3 SRP68	4 ALG6	2 RHOC	2 GABRA5	4 HLA.DMB
3 MYD88	4 ZNF189	2 ARPC1B	2 SLC28A2	4 MTHFD1
3 ARAF	4 SYCP2	2 FZD4	2 ATP2A2	4 POLA1
3 EIF2B1	4 ZNF214	2 VEGFB	2 LAMB1	4 GPHN
4 ATP5G1	4 OR13C4	2 BLOC1S1	2 KCNAB3	4 WARS2
4 NDUFB3	4 OR5T1	2 CALM3	2 P2RX6	4 CRTAM
4 PSMD13	4 SLC1A6	2 NDUFA1	2 SRC	4 CXCL9
4 PSMA8	4 OR6C3	2 UQCRC1	2 CTNNA1	4 RFC3
4 SEC61G	4 OR8H3	2 ITPR1	2 GNAI1	4 KLRC2
4 NDUFA12	4 OR5I1	2 NDUFS7	2 GABRG2	4 MCM10
4 POLA1	4 OR2A12	2 ATP5A1	2 MMP17	4 ETF1
4 XCL1	4 PI4K2B	2 CAMK2D	2 IFNA6	4 SNRPD1
4 CSF2	4 PNLIPRP2	2 AP3B1	2 PRLR	4 ORC6
4 FCER2	4 MOGS	2 PRKCA	2 PXDN	4 ARPC4
4 COX6B1	4 CD28	2 SLC2A4	2 GFRA2	4 FLT3LG
4 E2F1	4 LBP	2 KCNJ12	2 SCN8A	4 UMPS
4 IL12RB2	4 OR4K2	2 OR4A15	2 GJA9	4 POLR3E
4 TNFRSF9	4 OR6C4	2 GNGT2	2 MYLK3	4 RARS
4 SNRPD2	4 OR5M3	2 OR5V1	2 COL4A1	4 EIF4H
4 TIMP1	4 OR4C3	2 PRKCG	2 TACR1	4 IFITM2
4 ERCC5	4 CD36	2 NEFL	2 FMOD	4 PPIH
4 SUV420H1	4 SLCO1B1	2 WNT1	2 KCNG1	4 APEX1
4 RPL13A	4 OR6C76	2 COMT	2 LAMB2	4 CDC25A

4 SUPT4H1
4 TNFRSF4
4 DUSP3
4 ITGA4
4 AP1B1
4 SPCS1
4 PSMD11
4 SOCS2
4 RXRA
4 SNRPB2
4 VASP
4 ACVR1B
4 PFDN5
4 PRKAR2A
4 POLR2I
4 L2HGDH
4 RNF144B
4 ATP5I
4 POLR1D
4 SH3BP2
4 RPL26
4 RPL36A
4 RPSA
4 CCNT1
4 ICA1
4 TAF13
4 SEC61B
4 HLA.DRB1
4 IFNA8
4 TUBB6
4 MAF
4 PRKCE
4 POLR2K
4 HLA.E
4 TGFBR1
4 SMAD7
4 HCST
4 LDHA
4 DUSP4
4 APOBEC3G
4 CDK6
4 TXNL4A
4 SHC3
4 SF3B14
4 CXCR4
4 POLR2L
4 BCL2L14
4 ATP5J2
4 RAC1
4 IKZF1
4 CD79B
4 CDKN1A
4 EHD1
4 DHX8
4 EIF3G
4 WWTR1
4 SSR4
4 XAB2
4 HLA.DRA
4 TGFBR3
4 PSMB8
4 PLK1
4 NDUFA7
4 NAGLU
4 MNAT1
4 CIITA
4 GZMA
4 HIST3H2A
4 LIG4
4 UQCRQ
4 DUSP7
4 ETS2
4 CCDC12
4 HLA.B
4 NDUFS6
4 HGF
4 AP2S1
4 TADA2B
4 DAPK2
4 TAP1
4 D2HGDH
4 PSME1
4 DDB2
4 SLCO1C1
4 OR5J2
4 ABCC9
4 PEX11B
4 OR52A5
4 OR14J1
4 OR1A2
4 OR4C13
4 OR4C16
4 SLC38A4
4 ZNF552
4 ABCD2
4 ZNF599
4 AMY2A
4 UIMC1
4 LIPC
4 OR5R1
4 ZNF708
4 ZNF566
4 ZNF14
4 ZNF506
4 OR2T29
4 TAS2R8
4 OR2T5
4 OR4K5
4 OR2F2
4 OR9G1
4 AMY1A
4 ASB15
4 OR1D4
4 OR51G1
4 OR8K1
4 SLC7A9
4 OR2J3
4 OR8I2
4 UGT2A1
4 OR2L8
4 ROCK1
4 OR4F17
4 UGT2B11
4 OR4M1
4 OR5B2
4 OR7A10
4 OR8B3
4 ACP5
4 CEBPA
4 ZNF20
4 ZNF490
4 OR1C1
4 OR5T2
4 SLCO1A2
4 ZNF563
4 ELL
4 OR13C3
4 OR5W2
5 ERCC6L
5 RNF144B
5 ZNF479
5 LYPLA1
5 PMAIP1
2 HRH3
2 ECHS1
2 HADHA
2 TH
2 OR1E1
2 NDUFV3
2 DHH
2 CCR6
2 TF
3 KCNJ10
3 CALM2
3 HDAC5
3 GNAI1
3 PCCA
3 GSTZ1
3 DAGLB
3 IFNGR1
3 BTK
3 AP1S2
3 POLR2J2
3 ABCC4
3 IGF1R
3 CHP
3 PPP3R1
3 MGLL
3 ATP6V0B
3 ARHGDIB
3 ATP6V1A
3 ARRB1
3 FH
3 SNX2
3 ARHGAP12
3 UQCRQ
3 NDUFA12
3 AP3B2
3 COX7C
3 FTL
3 CLTC
3 FPGT
3 MCOLN1
3 UQCR10
3 VAMP7
3 SMS
3 AGA
3 CREB3L2
3 PGM2
3 AP1S1
3 CD58
3 STX7
3 ECT2
3 SUMF1
3 ATP6V1D
3 SNAP23
3 HEXB
3 LTBR
3 HSP90B1
3 MAPKAPK3
3 IFNGR2
3 MEF2BNB.MEF2
3 ARPC5
3 NPC2
3 NDUFB6
3 MITF
3 MGST3
3 MBOAT2
3 HYAL2
3 HTR7
3 DUSP6
3 CD63
3 CALML3
3 ARHGAP18
3 CACNA2D4
3 MYL3
3 FZD5
3 ATP6V1C1
3 CTSB
3 NANS
3 DEPDC1B
3 RHOA
3 NLRP3
3 ACSS2
3 SDHC
3 UQCRH
2 NR2E3
2 CA5A
2 CYP2U1
2 CNGA1
2 EFEMP2
2 SULT4A1
2 STX1A
2 UGT1A9
2 ACSM1
2 ADH1B
2 GPR17
2 GCLC
2 PODNL1
2 PRKACB
2 FZD1
2 MAOA
2 SMO
2 SPHK1
2 CASC5
2 TNFSF15
2 FGD2
2 PDE6A
2 TAS2R10
2 TYR
2 SCNN1G
2 COLEC11
2 NYX
2 PRLH
2 CACNA1B
2 DAG1
2 RAE1
2 CTSB
2 PDE4C
2 CRH
2 CXCL2
2 SDC1
2 SULT1A4
2 ACTA2
2 NUP88
2 C3
2 CYR61
2 CCL2
2 LRP1
2 COL1A2
2 NUP188
2 RXRG
2 MMP10
2 KCND1
2 ZP2
2 PTPRJ
2 PARVA
2 PDE1A
2 CHRNE
2 ARSE
2 MUC5AC
2 AP2B1
2 GRID2
2 ADCY9
2 FRAT1
2 P2RX2
2 ADCYAP1R1
2 NR1D2
2 CTGF
2 ERBB4
2 EMILIN1
2 TPMT
2 SEMA3F
2 CYP2F1
2 DNM1
2 PF4
2 CELA3A
2 GLRA3
2 CLTA
2 GJA1
2 SCTR
2 PYGO1
2 H2AFX
2 SPOCK3
2 COL18A1
2 SLC6A1
2 ADAM22
2 ADCY2
2 CAV2
4 TRRAP
4 SLC25A12
4 LMNB2
4 ARPC1B
4 UCHL5
4 CUL5
4 GALNT2
4 ESPL1
4 EXOSC9
4 AK2
4 MTX2
4 WARS
4 ACAT1
4 RAP1GAP2
4 FOXO1
4 MS4A1
4 PSMA3
4 BID
4 CEP290
4 TLE4
4 RMI1
4 RUVBL1
4 NNT
4 PSMA4
4 DOCK2
4 STAT1
4 SNRPA1
4 STK39
4 CENPQ
4 RCOR1
4 CUL2
4 DNA2
4 NME1
4 DLG1
4 ETFA
4 CDKN2C
4 GTF2F2
4 MAPK9
4 NHP2
4 EIF5B
4 POLD2
4 PSMB8
4 TUBG1
4 GNG10
4 PRIM1
4 ZEB1
4 ERCC6L
4 CCR6
4 PGAP1
4 TFAM
4 ACTL6A
4 CXCL10
4 UBE2C
4 DPYD
4 CCL20
4 CCNB1
4 PSMA2
4 CCNB2
4 KIF20A
4 NEK2
4 RACGAP1
4 SGMS1
4 JAK2
4 OIP5
4 XYLT1
4 SMS
4 ENTPD1
4 LYN
4 RBBP8
4 IL26
4 SLC25A32
5 PIGH
5 TNFSF13
5 ITGB2
5 IL5
5 IL4
5 TNFRSF11A
5 GAB2
5 LIF
5 FURIN
5 ITGAL
5 KLRD1
5 ZYX

4 MCM4
4 SH2D1B
4 STAT4
4 LOC646626
4 CD2
4 PPP3CC
4 HIST2H2AA3
4 GRAP2
4 CD3G
4 IRF8
4 PSME2
4 GBP2
4 KIR2DL3
4 NCOA1
4 KIR2DL2
4 SRPR
4 GZMB
4 IFNA7
4 IL2RB
4 TYROBP
4 PRMT1
4 PSIP1
4 BCL10
4 SKP2
4 NDUFA9
4 CABIN1
4 EXOSC7
4 FBXO5
4 PSMB10
4 FAM120B
4 PDIA3
4 PTPN11
4 PPIH
4 PIK3R5
4 RPS24
4 KIR2DS5
4 GTF2H4
4 RAC3
4 ZNF385A
4 TNFSF13
4 CDK9
4 RPLP2
4 ADAR
4 RIPK2
4 PARD3
4 YWHAH
4 GTF2A1
4 HLA.F
4 ASAH1
4 CD247
4 NUP37
4 RPS6KA4
4 EEF2K
4 SEC11C
4 NDUFS3
4 HLA.G
4 STK39
4 IDH3G
4 ZNF473
4 POLR2H
4 RCHY1
4 CD2BP2
4 GAB2
4 UQCRC1
4 BANF1
4 TSG101
4 SMAD1
4 PAPOLA
4 PSMD2
4 RNPS1
4 RPL26L1
5 HOXA7
5 TNFRSF10C
5 EIF6
5 TICAM1
5 COX8A
5 IFITM3
5 IL8
5 MAP3K8
5 TNFSF14
5 IFITM2
5 SDC4
5 XPA

3 RAP1A
3 SRGN
3 STARD8
3 CDK5R1
3 KIF15
3 ADRBK2
3 SORT1
4 ADCY7
4 NAGLU
4 FZD2
4 NCF4
4 IDH2
4 DLG4
4 OR10J5
4 RHOBTB2
4 OR10A4
4 BACE1
4 SYK
4 C4BPA
4 HAP1
4 KCNK9
4 SLC17A7
4 IVD
4 ADHFE1
4 BCL2
4 GIPR
4 GLG1
4 KCNAB2
4 NLRX1
4 GSTA4
4 BCKDHA
4 HADH
4 CTSS
4 KCNK13
4 DRD2
4 DUSP3
4 PAK1
4 TNFSF13
4 HLA.DRB1
4 HLA.DRB5
4 HLA.DPB1
4 SLC8A1
4 HLA.DPA1
4 HLA.DRA
4 KCNK6
4 PRCP
4 LY86
4 MGST2
4 KCNQ1
4 CFD
4 CYBRD1
4 TLR5
4 PECAM1
4 CD36
4 CSF3R
4 TXNIP
4 WNT10B
4 GLB1
4 PLA2G15
4 ACAA2
4 MANBA
4 SERPINF2
4 TAB1
4 CDH3
4 KCNJ1
4 ARHGAP26
4 CACNA2D3
4 ALDH2
4 LRP1
4 PLBD1
4 RNF135
4 CD33
4 PYCARD
4 ITGAM
4 CD4
4 C5
4 CD1D
4 NAGA
4 PLA2G2A
4 GAA
4 CHRM1
4 FGD4
4 MEFV

2 CTF1
2 GHSR
2 NPFFR1
2 WISP2
2 SV2C
2 ATP1A3
2 ARSA
2 SGCB
2 CNTN6
2 TRPC1
2 MYH11
2 THRA
2 CYP4F12
2 CYP2D6
2 SLC6A3
2 SEMA6C
2 CAMK2A
2 MSH4
2 F10
2 DIAPH1
2 ATP6V0A4
2 HRH1
2 KCNQ4
2 SLC15A1
2 CACNA1G
2 FGB
2 LOXL2
2 SLC15A2
2 GABRR2
2 GAD1
2 CRELD1
2 CTH
2 GUCA1B
2 SNAP25
2 MYH8
2 MYL4
2 SGCD
2 SLC43A1
2 SLC1A6
2 SLC3A1
2 IFNW1
2 SLC38A2
2 SEMG1
2 OR7A10
2 BCAN
2 HYAL3
2 CACNA1I
2 VWA1
2 LAMC3
2 CYP4F8
2 S100A3
2 HMOX2
2 PDPK1
2 CACNG1
2 OR2F2
2 CDH6
2 OPLAH
2 CYP3A4
2 GREM1
2 DES
2 PAPPA
2 GRIK2
2 NR1I2
2 CACNA2D3
2 COL9A1
2 MMP9
2 CYP21A2
2 FCN1
2 KNG1
2 MTNR1A
2 HTR5A
2 KCNF1
2 AGRN
2 RRAS
2 ALDH3B2
2 AR
2 OR10H2
2 HIST2H4B
2 PLCB2
2 PNLIP
2 PDE1B
2 PFN2
2 GCKR

5 DGCR8
5 PTK2B
5 CCR1
5 CENPB
5 CHST12
5 CCL4
5 RABGGTB
5 SARS2
5 CD8A
5 IL4R
5 XCL1
5 FLNA
5 IDH2
5 ATP6V0E2
5 FASLG
5 ZBTB16
5 CD244
5 MAP4K1
5 ARRB2
5 B3GAT3
5 CAPN1
5 MYO18A
5 HIC1
5 CAMK2G
5 FKBP1A
5 NUP205
5 BUB3
5 PCNA
5 RBM25
5 TOMM20
5 MCM3
5 TRAF1
5 HLA.F
5 IL2RB
5 PSMD11

5	FOSL1	4	WNT9A	2	ALDH1A2
5	CCND2	4	HK3	2	COMT
5	NDRG1	4	OR6N1	2	PDGFB
5	USH1C	4	OR6K2	2	TJP1
5	TIAM1	4	HTR1E	2	CRNN
5	PDK1	4	MAN2B1	2	CYP4A11
5	JUND	4	OR8H1	2	LTBP1
5	HIC1	4	CD1A	2	UTS2
5	FYN	4	TAAR8	2	GSTM4
5	CR2	4	OR1S2	2	LMNA
5	ERCC2	4	GBA3	2	PTGER2
5	TUBA3C	4	MYH10	2	HIST2H2AA3
5	PPP3CA	4	RAB3A	2	COL9A2
5	SHFM1	4	OR10J3	2	FMO3
5	NDUFA11	4	MAP2K6	2	SMPD1
5	PAK4	4	ADRB2	2	GNAT1
5	LIF	4	ALDH3A2	2	PLA2G4B
5	CDC45	4	GALK1	2	CST7
5	NFKBIE	4	CREB3L4	2	ADAM19
5	REL	4	OR4D2	2	STX16
5	TNFRSF1B	4	FZD1	2	SSTR2
5	SERPINE1	4	TLR4	2	P4HTM
5	PPP2R5C	4	ATP6V0A1	2	NPR1
5	PLK3	4	HYAL1	2	KCNA6
5	PAG1	4	DEFA5	2	ADAM23
5	LTA	4	HLA.DQA1	2	CYP2C9
5	IRF4	4	HLA.DQA2	2	CSNK1E
5	IL2	4	HLA.DMA	2	PLA2G4C
5	HSP90B1	4	TRIO	2	GGT5
5	GADD45B	5	DAGLA	2	MTNR1B
5	CREM	5	PIK3R6	2	KLKB1
5	BCL2A1	5	TLR7	2	BMP1
5	CCL20	5	EEA1	2	RXRB
5	USP6NL	5	CTSD	2	ANK1
5	TRAF1	5	ACP5	2	EMD
5	SPRY1	5	GNPDA1	2	MYH14
5	SH2D2A	5	NANP	2	GCLM
5	PTPN6	5	SLC18A3	2	CSNK1G1
5	POMC	5	SLC16A1	2	NR4A1
5	JUNB	5	CAMK1	2	PLOD2
5	ICOS	5	CD276	2	SFRP1
5	FOSB	5	ARHGAP31	2	SLC7A5
5	DUSP5	5	RILP	2	COMP
5	DDIT4	5	ATP6V1F	2	SERPINA6
5	BTLA	5	CTSZ	2	NEU1
5	CD40LG	5	COX5A	2	SYNE1
5	TNF	5	YWHAH	2	CSPG4
5	IFITM1	5	NNT	2	F12
5	IL3	5	GNPTAB	2	YES1
5	NFKBIA	5	GM2A	2	ITGB1
5	NR4A1	5	G6PD	2	GALR1
5	FOSL2	5	CYC1	2	PXN
5	FOS	5	ATP2A2	2	NGF
5	EGR3	5	BCAT2	2	GJA8
5	EGR2	5	ATP6V1B2	2	HTR7
5	EGR1	5	GPX4	2	ADRA2C
5	BTG2	5	PLA2G4C	2	PTGES
5	DOCK2	5	ARHGAP10	2	CLDN4
5	ZFP36	5	IDH3G	2	KCNK7
5	C4BPA	5	AP2S1	2	NOS2
5	MYC	5	CD74	2	COL1A1
5	ARHGEF18	5	ND3	2	GP9
5	TUBA1C	5	CANX	2	PLAU
5	UCP3	5	SUMF2	2	CXXC4
5	YWHAG	6	RENBP	2	LHB
5	ETFB	6	HLA.DQB1	2	SULT2B1
5	TUBA4A	6	HLA.DRB3	2	NR1H2
5	NDUFA1	6	GNB4	2	SLC26A3
5	IL21R	6	ATP1B1	2	HPGDS
5	EIF4A1	6	OPLAH	2	C1QA
5	TUBB2A	6	KCNMB1	2	DNM2
5	CSK	6	CD1E	2	EGFL7
5	TUBA1A	6	CIITA	2	MC1R
5	HNRNPA0	6	TAAR2	2	PAK7
5	BCAS2	6	C3	2	SLC1A5
5	EGR4	6	PTPRF	2	SORBS1
5	ANP32A	6	TNFSF13B	2	IL16
5	CCR4	6	AMDHD2	2	KCNMB2
5	LIMK1	6	PDCD1LG2	2	SEMA3A
5	PTTG1	6	SERPING1	2	PVR
5	SNRPF	6	IL2	2	RGS2
5	NDUFA13	6	GNG4	2	FBLN2
5	SMAD2	6	CTSC	2	EPO

5 HSPG2
5 GTF2H2
5 PSMA7
5 HIST1H3B
5 MAP2K3
5 TROVE2
5 SH2B3
5 BIRC2
5 LIN7A
5 POMP
5 NDUFV3
5 PSMD8
5 MAP4K2
5 BIRC3
5 MAPK6
5 CD7
5 PDPK1
5 RASGRP2
5 EIF3C
5 HSPA2
5 PRPF4
5 TAF9

6 C2
6 C1QC
6 C1QA
6 C1QB
6 NAGK
6 PDGFB
7 AP1M2
7 DRD3
7 OR5L2
7 CREB3L1
7 GPX3
7 CD86
7 PLA2G1B
7 ARHGAP44
7 ICA1
7 HGSNAT
7 OR1N2
7 GNAQ
7 OR10G8
7 PCK1
7 DGKD
7 PRKCB
7 BACE2
7 CD180
7 KCNH8
7 ITGA4
7 ARHGEF4
7 CTSG
7 DEFA4
7 MEF2C
8 CTSK
8 STARD13
8 GP9
8 ASAH1
8 PPT1
8 NPL
8 RHOB
8 EPB41L1
8 CTSL2
8 ITSN1
8 HMOX1
8 OSBPL1A
8 PLTP
8 OPHN1
8 SLC46A1
8 PDK4
8 F13A1
8 KCNMB4
8 VIPR1
8 TPM2
8 NRCAM
8 ARHGAP22
8 FUCA1
8 FAM13A
8 HEXA
8 GPX1
8 GCLC
8 GP1BB
8 HGF
8 SLC40A1
8 OCRL
8 GSTT2
8 DEFA3
8 DEPDC7
9 SIGLEC1
9 KCNJ9
9 LGMN
9 UCHL1
9 CXCL12
9 GRIK3
9 UNC13B
9 DNASE2
9 IDH1

2 POMC
2 NEFL
2 CADM1
2 PTAFR
2 TNFSF11
2 ADAMTS7
2 FNDC8
2 PDE7B
2 NPR2
2 KCNJ12
2 ADM2
2 GLI1
2 FCGR2B
2 SLC1A3
2 PRNP
2 GJA3
2 CYP11B2
2 ABCB6
2 CCKAR
2 KCNK1
2 AOX1
2 BMP8B
2 GAS6
2 CLDN6
2 IL2
2 PTGDR
2 MLLT4
2 PLA2G2A
2 PRKACG
2 FGF6
2 HTR6
2 ADRA2B
2 CST5
2 CYP2C8
2 C4BPA
2 ITGB4
2 ITGB8
2 PLA2G5
2 GNAT2
2 SLC7A7
2 CA7
2 LLGL1
2 HTR2B
2 KITLG
2 GALR3
2 PRG3
2 SGCA
2 MFAP4
2 PDE10A
2 CYP3A7
2 FFAR3
2 GJB1
2 SYT1
2 HIST1H2AM
2 MITF
2 CMA1
2 MCF2
2 MMP11
2 MMP26
2 NR5A1
2 P2RY6
2 PIK3CB
2 TALDO1
2 ITIH3
2 ARHGEF11
2 NR2F6
2 CACNA2D1
2 TIMP4
2 SLC6A20
2 CELA2A
2 KCNK10
2 GJA5
2 IBSP
2 RHCG
2 ALOX15
2 ATP2B2
2 FIGF
2 APOE
2 AVPR2
2 CLEC4A
2 GNAL
2 CALCA
2 WNT4

2	DMP1
2	NCAM1
2	DCC
2	CLEC4M
2	GAL
2	MUC2
2	VCAM1
2	GPR4
2	IL9
2	SHH
2	SRGAP2
2	SPOCK1
2	S100A1
2	CHRNA2
2	PLXNA3
2	CACNG4
2	SFRP5
2	TPO
2	GSTA3
2	SCN3B
2	COL8A1
2	CLDN5
2	ALB
2	WNT3
2	SPARCL1
2	MGP
2	CALCR
2	INHA
2	HIST1H2BI
2	SERPINA5
2	EPB41L1
2	REG1A
2	PTGS2
2	SLC2A4
2	MPDZ
2	BMP5
2	IGFBP7
2	APLNR
2	CYSLTR1
2	CXCL11
2	KCNH4
2	NCAN
2	CLDN11
2	DRD3
2	SEMA3C
2	DAGLA
2	CAMK2B
2	RGR
2	DGKG
2	AQP5
2	HRH2
2	LAMA5
2	ITGA5
2	VEGFA
2	IMPG2
2	MCHR1
2	OR7E24
2	SCNN1B
2	CRHBP
2	KCNN1

Supplementary Table S5: Functional Enrichment of PPA networks

(All unique functions are in bold and italics)

EXPERIMENT: E-GEOD-4209, Downregulated Canonical Pathways

Maximum stable clusters : 5

Functional Enrichment p -value $<10^{-5}$

cluster	KEGG pathway	p-value	# of proteins	Reactome pathway	p-value	# of proteins
1	Spliceosome	1.759e^-23	31	Spliceosomal B complex	1.410e^-17	24
1	T cell receptor signaling pathway	1.759e^-23	29	Formation of the Spliceosomal B complex	1.410e^-17	24
1	Metabolic pathways	1.209e^-16	70	mRNA splicing- major pathway	3.230e^-17	25
1	***Neurotrophin signaling pathway***	9.749e^-15	23	Spliceosomal active C complex with lariat containing 5'-end	1.190e^-16	24
1	***Natural killer cell mediated cytotoxicity***	2.729e^-13	22	cleaved pre-mRNP:CBC complex		
1	RNA transport	3.880e^-13	23	Lariat formation and 5'- splice site cleavage	1.190e^-16	24
1	***Chronic myeloid leukemia***	4.599e^-13	17	Formation of an intermediate spliceosomal C complex	1.239e^-16	24
1	***Fc epsilon RI signaling pathway***	1.800e^-12	17	Exon junction complex	1.239e^-16	24
1	***Measles***	2.279e^-12	21	Cleavage at the 3'-splice site and exon ligation	1.239e^-16	24
1	Cell cycle	4.360e^-12	20	Spliceosomal intermediate C complex	7.859e^-16	23
1	B cell receptor signaling pathway	1.009e^-11	16	Spliceosomal active C complex	7.859e^-16	23
1	Pyrimidine metabolism	1.049e^-11	18	mRNA splicing-minor pathway	4.580e^-12	14
1	Huntington's disease	1.730e^-11	23	ATAC B complex	4.580e^-12	14
1	***Pancreatic cancer***	1.799e^-11	15	ATAC C complex with lariat containing 5'-end cleaved mRNA	4.580e^-12	14
1	Pathways in cancer	1.799e^-11	30	***ATAC C complex***	4.580e^-12	14
1	Oocyte meiosis	3.780e^-11	18	***Formation AT-AC C complex***	4.580e^-12	14
1	***ErbB signaling pathway***	6.739e^-11	16	ATAC spliceosome mediated 3' splice site cleavage, exon	4.580e^-12	14
1	***Pathogenic Escherichia coli infection***	1.949e^-10	13	ligation		
1	***Fc gamma R-mediated phagocytosis***	2.210e^-10	16	Spliceosomal A complex	4.809e^-12	18
1	Focal adhesion	4.780e^-10	22	***Transport of mature Mrna derived from an Intron-containing***	4.809e^-12	15
1	Purine metabolism	4.980e^-10	20	***Docking of the TAP:EJC complex with the NPC***	7.590e^-11	14
1	Alzheimer's disease	5.980e^-10	20	RNA polymerase 2 pre-transcription events	1.080e^-8	13
1	Chemokine signaling pathway	6.870e^-10	21	Degradation of multiubiquitinated Securin	5.860e^-8	13
1	MAPK signaling pathway	7.090e^-10	25	Formation of the Spliceosomal E complex	5.860e^-8	13
1	***Influenza A***	8.890e^-10	20	Spliceosomal E complex	5.860e^-8	13
1	***Regulation of actin cytoskeleton***	8.890e^-10	22	Degradation of multiubiquitinated cell cycle proteins	1.290e^-7	13
1	Parkinson's disease	3.359e^-9	17	***APC/C:Cdh1 mediated degradation of Cdc20 and other***	1.509e^-7	13
1	Nucleotide excision repair	6.139e^-9	11	APC/C:Cdh1 targeted proteins in late mitosis/early G1		
1	***Glioma***	1.480e^-8	12	snRNP assembly	1.790e^-7	11
1	***Non-small cell lung cancer***	3.019e^-8	11	***Release from the NPC and disassembly of the mRNP***	2.519e^-7	10
1	***Hepatitis C***	3.369e^-8	16	Degradation of multiubiquitinated Cdh1	2.820e^-7	12
1	***Progesteron-mediated oocyte maturation***	4.540e^-8	13	Autodegradation of Cdh1 by Cdh1:APC/C	2.820e^-7	12
1	***Renal cell carcinoma***	4.799e^-8	12	***RNA pol 3 simple start sequence initiation at type 3***	3.060e^-7	9
1	***Acute myeloid leukemia***	5.009e^-8	11	***promoters***		
1	***Jak-STAT signaling pathway***	5.359e^-8	17	***RNA pol 3 promoter opening at type 3 promoters***	3.060e^-7	9
1	***Leukocyte transendothelial migration***	2.179e^-7	14	***RNA pol 3 transcription initiation from type 3 promoter***	3.060e^-7	9
1	***Toxoplasmosis***	2.320e^-7	15	***RNA pol 3: TF3B:SNAPc:type 3 open promoter complex***	3.060e^-7	9
1	***GnRH signaling pathway***	2.990e^-7	13	***RNA pol 3 :TF3B:SNAPc:type 3 promoter complex***	3.060e^-7	9
1	Ubiquitin mediated proteolysis	2.990e^-7	15	Intron-containing complex	3.590e^-7	10
1	***Toll-like receptor signaling pathway***	3.730e^-7	13	Formation of RNA pol 2 elongation complex	4.529e^-7	10
1	Osteoclast differentiation	5.770e^-7	14	Formation of HIV-1 elongation complex in the absence of	4.529e^-7	10
1	***Colorectal cancer***	7.190e^-7	10	HIV-1 Tat		
1	***Shigellosis***	7.190e^-7	10	***APC/C:Cdh1-mediated degradation of Skp2***	4.899e^-7	12
1	Oxidative phosphorylation	1.089e^-6	14	***RNA pol 3 abortive and retractive initiation***	5.689e^-7	10
1	***Epithelial cell signaling in Helicobacter pyl***	2.430e^-6	10	***Transport of the SLBP dependent mature mRNA***	7.579e^-7	9
1	***Viral myocarditis***	3.660e^-6	10	Cdc20:phospho-APC/C mediated degradation of Cyclin A	7.859e^-7	12
1	***Gap junction***	3.750e^-6	11	***Degradation multiubiqitinated Cyclin A***	7.859e^-7	12
1				***CD28 dependent Vav1 pathway***	8.129e^-7	6
1				***3'-polyadenylated, capped mRNA complex***	9.159e^-7	8
1				ATAC A complex	9.350e^-7	9
1				***TAP:3'-polyadenylated, capped mRNA complex***	1.289e^-6	8
1				***Transport of the export-competent complex through the NPC***	2.129e^-6	9
1				***Signaling by SCF-KIT***	2.129e^-6	9
1				mRNA capping	2.600e^-6	8
1				***RNA pol 2 transcription elongation***	3.289e^-6	9
1				Recruitment of elongation factors to form HIV-1 elongation	3.289e^-6	9
1				complex		
1				Addition of nucleotides leads to transcript elongation	3.289e^-6	9
1				Elongation complex	3.289e^-6	9
1				HIV-1 elongation complex	3.289e^-6	9
2	Metabolic pathways	4.34E-30	83	***Respiratory electron transport***	2.290e^-29	30
2	Alzheimer's disease	7.53E-40	44	Exon Junction Complex	7.959e^-20	26
2	Huntington's disease	1.19E-37	44	Cleavage at the 3'-Splice site and Exon Ligation	7.959e^-20	26
2	Oxidative phosphorylation	9.59E-35	37	mRNA Splicing- Major Pathway	1.039e^-19	26
2	Parkinson's disease	3.06E-32	35	Spliceosomal Intermediate C Complex	3.549e^-19	25
2	Spliceosome	5.33E-14	20	Spliceosomal Active C Complex	3.549e^-19	25
2	***Proteasome***	1.53E-22	19	Spliceosomal Active C Complex with lariat containing, 5'-end	3.549e^-19	25
2	Pathways in cancer	4.11E-06	19	Cleaved pre-Mrnp:CBC complex		

2	Purine metabolism	3.49E-09	17	Lariat Formation and 5'-Splice Site Cleavage	3.549e^-19	25
2	B cell receptor signaling pathway	9.96E-14	16	Spliceosomal A Complex	3.730e^-19	23
2	RNA transport	6.46E-09	16	Formation of an Intermediate Spliceosomal C Complex	3.730e^-19	25
2	T cell receptor signaling pathway	4.47E-09	14	***Degradation of ubiquitinated p27/p21 by the 26s proteasome***	2.370e^-18	19
2	Osteoclast differentiation	2.86E-08	14	***SCF(Skp2)-mediated degradation of p27/p21***	2.370e^-18	19
2	Ubiquitin mediated proteolysis	9.56E-08	14	***APC/C:Cdh1 mediated degrdation of Cdc20 and other***	3.240e^-18	21
2	Cell cycle	1.96E-07	13	APC/C:Cdh1 targeted proteins in late mitosis/early G1		
2	Pyrimidine metabolism	1.50E-07	12	Spliceosomal B Complex	3.829e^-18	23
2	Focal adhesion	1.89E-04	12	Formation of the Spliceosomal B Complex	3.829e^-18	23
2	***Long-term potentiation***	2.24E-08	11	***Complex 1 - NADH: Ubiquinone oxidoreductase***	5.799e^-18	18
2	***Apoptosis***	2.80E-07	11	***NADH enters the respiratory chain at Complex 1***	5.799e^-18	18
2	Oocyte meiosis	2.89E-06	11	***APC/C: Cdh 1 - mediated degradation of Skp2***	8.690e^-18	20
2	***Cardiac muscle contraction***	6.50E-07	10	Degradation of multiubiquitinated Securin	8.690e^-18	20
2	***Valine, leucine and isoleucine degrada***	8.51E-07	8	***APC/C:Cdc20 mediated degradation of Securin***	8.690e^-18	20
2	***Protein export***	6.58E-08	7	Formation of the Spliceosomal E complex	8.690e^-18	20
2	***RNA polymerase***	5.41E-07	7	Spliceosomal E Complex	8.690e^-18	20
2				***26s proteosome degrades ODC holoenzyme complex***	1.139e^-17	18
2				***26s proteasome***	1.290e^-17	17
2				***Cdc20: Phospho-APC/C mediated degradation of Cyclin A***	2.159e^-17	20
2				***Degradation of multiubiquinated Cyclin A***	2.159e^-17	20
2				***Degradation of multiubiquinated cell cycle proteins***	2.930e^-17	20
2				***Autodegradation of the E3 ubiquitin ligase COP1***	3.519e^-17	18
2				***Ubiquitin mediated degradation of phosphorykated Cdc25A***	3.519e^-17	18
2				Degradation of multiubiquitinated Cdh1	3.980e^-17	19
2				Autodegradation of Cdh1 by Cdh1:APC/C	3.980e^-17	19
2				***Destruction of AUF1 and Mrna***	4.340e^-17	17
2				***Proteasomal cleavage of exogenous antigen***	4.340e^-17	17
2				***Antigen processing : Ubiquitination and Proteasome degradation***	6.460e^-17	18
2				***SCF-mediated degradation of Emi 1***	6.460e^-17	18
2				***SCF-beta-***	6.460e^-17	18
2				***Cross-presentation of soluble exogenous antigens(endosomes)***	1.600e^-16	17
2				***Activation of NF-kappaB in B cells***	2.029e^-16	18
2				***Proteasomal cleavage of substrate***	2.320e^-16	17
2				***Regulation of activated PAK-2p34 by proteasome mediated***	2.989e^-16	17
2				***Proteasome mediated degradation of COP1***	2.989e^-16	17
2				***Proteolytic degradation of ubiquitinated-Cdc25A***	2.989e^-16	17
2				***Ubiquitinated Cdc6 is degrated by the proteasome***	2.989e^-16	17
2				***Ubiquitinated Orc1 is degrated by the proteasome***	2.989e^-16	17
2				***Proteasome mediated degradation of PAK-2p34***	2.989e^-16	17
2				***Proteasome mediated degradation of Cyclin D1***	2.989e^-16	17
2				***Ubiquitinated geminim is degraded by the proteasome***	2.989e^-16	17
2				***Ubiquitin - dependent degradation of Cyclin D1***	4.380e^-16	17
2				***CDK-mediated phosphorylation and removal of Cdc6***	4.380e^-16	17
2				***Destabilization of mRNA by AUF1(hnRNP DO)***	9.659e^-16	17
2				***Degradation of beta-catenin by the destruction complex***	2.009e^-15	17
2				***Degradation of ubiquitinated-beta catenin by the proteasome***	2.009e^-15	17
2				***CDT1 association with the CDC6: ORC: origin complex***	5.850e^-15	17
2				Orc1 removal from chromatin	6.089e^-15	18
2				***ER- phagosome pathway***	8.099e^-15	18
2				***HP subcomplex***	3.139e^-14	14
2				Intron-containing complex	1.320e^-10	12
2				ATAC A Complex	3.759e^-10	11
2				mRNA splicing - Minor pathway	5.960e^-9	11
2				ATAC B Complex	5.960e^-9	11
2				ATAC C Complex with lariat containing 5'-end cleaved mRNA	5.960e^-9	11
2				***ATAC C Complex***	5.960e^-9	11
2				***Formation of AT-AC C Complex***	5.960e^-9	11
2				ATAC spliceosome mediated 3' splice site cleavage, exon ligatior	5.960e^-9	11
2				***Capped, methylated pre-mRNP:CBC complex***	1.460e^-8	10
2				RNA polymerase 2 pre-transcription events	1.619e^-8	12
2				***Hypophosphorylation of RNA pl 2 CTD by FCP1P protein***	1.790e^-8	9
2				snRNP assembly	2.279e^-8	11
2				***U2 snRNP***	3.669e^-8	8
2				***snRNP nuclear import and release***	6.490e^-8	10
2				***Formation of the Early Elongation Complex***	8.239e^-8	9
2				***Formation of the HIV-1 Early Elongation Complex***	8.239e^-8	9
2				Formation of RNA pol 2 elongation complex	8.239e^-8	10
2				**Formation of HIV-1 elongation complex in the absence of HIV-1 Tat**	8.239e^-8	10
2				Microtubule-bound kinetochore	6.330e^-7	12
2				Kinetochore	6.330e^-7	12
2				mRNA capping	6.959e^-7	8
2				***RNA polymerase 2 transcription elongation***	6.959e^-7	9
2				***Recruitment of elongation factors to form HIV-1 elongation complex***	6.959e^-7	9
2				***Addition of nucleotides 5 through 9 on the growing transcript***	6.959e^-7	9
2				Addition of nucleotides leads to transcript elongation	6.959e^-7	9
2				Elongation complex	6.959e^-7	9
2				***HIV-1 transcription complex containing 4 nucleotide long transcript***	6.959e^-7	9

2	*Addition of nucleotides 5 through 9 on the growing HIV-1 transcript*	6.959e^-7	9
2	*pol 2 transcription complex containing 4 nucleotide long transcript*	6.959e^-7	9
2	HIV-1 elongation complex	6.959e^-7	9
2	*snRNP Sm core complex*	6.959e^-7	5
2	*U11 snRNP*	6.959e^-7	5
2	*U4 ATAC snRNP*	6.959e^-7	5
2	*U4 ATAC snRNP: U6 ATAC snRNP*	6.959e^-7	5
2	*Spliceosomal m3G capped snRNA loaded with the SM complex*	6.959e^-7	5
2	*U6 ATAC snRNP*	6.959e^-7	5
2	*RNA polymerase 2 transcription initiation*	6.959e^-7	9
2	*RNA polymerase 2 transcription pre-initiation and promoter opening*	6.959e^-7	9
2	*RNA polymerase 2 promoter opening: first transition*	6.959e^-7	9
2	*newly formed phosphodiester bond stabilized and Ppi released*	6.959e^-7	9
2	*RNA polymerase 2 promoter escape*	6.959e^-7	9
2	*pol 2 promoter escape complex*	6.959e^-7	9
2	*HIV-1 transcription initiation*	6.959e^-7	9
2	*Transcription of the HIV genome*	6.959e^-7	9
2	*Addition of the fourth nucleotide on the nascent transcript: second t*	6.959e^-7	9
2	*HIV-1 transcription complex containing 3 nucleotide long transcript*	6.959e^-7	9
2	**HIV-1 initiation complex with phosphodiester-Ppi intermediate**	6.959e^-7	9
2	**HIV-1 open pre-initiation complex**	6.959e^-7	9
2	**HIV-1 promoter escape complex**	6.959e^-7	9
2	**RNA polymerase 2 HIV-1 promoter escape**	6.959e^-7	9
2	**NTP binds active site of RNA polymerase 2**	6.959e^-7	9
2	**Nucleophillic attack by 3'- hydroxyl Oxygen of nascent transcript on**	6.959e^-7	9
2	**Addition of the third nucleotide on the nascent transcript**	6.959e^-7	9
2	**HIV-1 transcription complex**	6.959e^-7	9
2	**pol 2 transcription complex containing 3 nucleotide long transcript**	6.959e^-7	9
2	**pol 2 initiation complex with phosphodiester-Ppi intermediate**	6.959e^-7	9
2	**pol 2 colsed pre-initiation complex**	6.959e^-7	9
2	**pol 2 initiation complex**	6.959e^-7	9
2	**pol 2 open pre-initiation complex**	6.959e^-7	9
2	**HIV-1 promoter opening: first transition**	6.959e^-7	9
2	**Addition of the third nucleotide on the nascent HIV-1 transcript**	6.959e^-7	9
2	**Addition of the fourth nucleotide on the nascent HIV-1 transcript: se**	6.959e^-7	9
2	*Fall back to closed pre-initiation complex*	6.959e^-7	9
2	*Nucleophillic attack by 3'- hydroxyl Oxygen of nascent HIV-1 transcri*	6.959e^-7	9
2	*newly formed phosphodiester bond stabilized and Ppi released pre-i*	6.959e^-7	9
2	*NTP binds active site of RNA polymerase 2 in HIV-1 open*	6.959e^-7	9
2	*HIV-1 closed pre-initiation complex*	6.959e^-7	9
2	*pol 2 transcription complex*	6.959e^-7	9
2	*HIV-1 initiation complex*	6.959e^-7	9
2	*U12 snRNP*	1.419e^-6	6
2	*Addition of nucleotides between position +11 and +30*	2.349e^-6	7
2	*pol 2 transcription complex with (ser5) phosphorylated CTD containi*	2.349e^-6	7
2	*pol 2 transcription complex containing extruded transcript to +30*	2.349e^-6	7
2	*pol 2 transcription complex containing 11 nucleotide longtranscript*	2.349e^-6	7
2	*pol 2 transcription complex containing 9 nucleotide long transcript*	2.349e^-6	7
2	*Phosphorylation (ser5) of RNA pol 2 CTD*	2.349e^-6	7
2	*Addition of nucleotides 10 and 11 on the growing transcript:third tra*	2.349e^-6	7
2	*Addition of nucleotides between position +11 and +30 on HIV-1 tran*	2.349e^-6	7
2	*RNA polymerase 2: NTP: TF2F complex*	2.349e^-6	7
2	*pol 2 transcription complex containing transcript to +30*	2.349e^-6	7
2	*HIV-1 transcription complex containing 9 nucleotide long transcript*	2.349e^-6	7
2	*HIV-1 transcription complex containing 11 nucleotide long transcript*	2.349e^-6	7
2	*Addition of nucleotides 10 and 11 on the growing HIV-1 transcript: t*	2.349e^-6	7
2	*HIV-1 transcription complex containing extruded transcript to +30*	2.349e^-6	7
2	*HIV-1 transcription complex containing transcript to +30*	2.349e^-6	7
2	*HIV-1 transcription complex with (ser5) phosphorylated CTD contain*	2.349e^-6	7
2	*RNA polymerase 2 CTD(phosphorylated) binds to CE*	3.279e^-6	7
2	*RNA pol 2 with phosphorylated CTD: CE complex with activated GT*	3.279e^-6	7
2	*RNA pol 2 with phosphorylated CTD: CE complex*	3.279e^-7	7
2	*cNAP-1 depleted centrosome*	3.539e^-6	10
2	*Nlp-depleted centrosome*	3.539e^-6	10
2	*Plk-1-mediated phosphorylation of Nlp*	3.850e^-6	10
2	*Loss of C-Nap-1 from centrosome*	3.850e^-6	10
2	*Centrosome containing recruited CDK11p58*	3.850e^-6	10
2	*Centrosome*	3.850e^-6	10
2	*Centrosome associated Plk1*	3.850e^-6	10
2	*Loss of Nlp from mitotic centrosomes*	3.850e^-6	10
2	*Centrosome containing phosphorylated Nlp*	3.850e^-6	10
2	*U1 snRNP*	3.850e^-6	5
2	*u6 snRNP*	3.850e^-6	5
2	*U4 snRNP:U6 snRNP complex*	3.850e^-6	5
2	*Transfer of GMP from the capping enzyme GT site to 5'-end of mRNA*	3.850e^-6	7
2	*Methylation of GMP-cap by RNA methyltransferase*	3.850e^-6	7
2	*SPT5 subunit of pol 2 binds the RNA triphosphatase (RTP)*	3.850e^-6	7

2				***Capping complex (initial)***	3.850e^-6	7
2				***Hydrolysis of the 5'- end of the nascent transcript by the capping en***	3.850e^-6	7
2				***RNA pol 2 CTD phosphorylation and interaction with CE***	3.850e^-6	7
2				***Formation of the CE:GMP intermediated complex***	3.850e^-6	7
2				***pol 2 transcription complex containing 4-9 nucleotide long transcript***	3.850e^-6	7
2				***CE:Pol 2 CTD: Spt5 complex***	3.850e^-6	7
2				***HIV-1 transcription complex containing 4-9 nucleotide long transcrip***	3.850e^-6	7
2				***Capping complex(intermediate)***	3.850e^-6	7
2				***Capping complex(hydrolyzed)***	3.850e^-6	7
2				***Covalent CE: GMP intermediate complex***	3.850e^-6	7
2				***Capping complex (with freed 5'-GMP)***	3.850e^-6	7
2				Capping complex(GpppN..) protein	3.850e^-6	7
3	No match			No match		
4	Cell cycle	1.369e^-24	27	***Activation of the pre-replicative complex***	5.280e^-20	16
4	***DNA replication***	1.139e^-23	18	***Activation of ATR in response to replication stress***	8.130e^-19	16
4	Pyrimidine metabolism	8.950e^-15	18	***DNA polymerase alpha: primase binds at the origin***	8.629e^-19	15
4	Purine metabolism	4.200e^-9	16	Microtubule-bound kinetochore	2.170e^-14	17
4	Metabolic pathways	4.200e^-9	41	Kinetochore	2.170e^-14	17
4	Nucleotide excision repair	4.200e^-9	10	***DNA polymerase epsilon binds at the origin***	2.170e^-14	12
4	***Mismatch repair***	5.149e^-9	8	***RPA: Cdc45:CDK:DDK:Mcm10:pre-replicative complex***	4.289e^-13	11
4	Chemokine signaling pathway	1.559e^-7	15	***Cdc45:CDK:DDK:Mcm10:pre-replicative complex***	2.099e^-12	10
4	Cytokine-cytokine receptor interaction	4.309e^-7	17	***Activation of claspin***	3.189e^-12	10
4	Oocyte meiosis	1.480e^-6	11	***Cdc45:CDK:DDK:Mcm10:claspin:pre-replicative complex***	3.189e^-12	10
4	***p53 signaling pathway***	1.839e^-6	9	***Cdc45:CDK:DDK:Mcm10:activated claspin:pre-replicative complex***	3.189e^-12	10
4				***Activation of E2F target genes at G1/S***	***1.310e^-11***	***9***
4				Orc1 removal from chromatin	1.790e^-11	14
4				***Removal of licensing factors from origins***	2.569e^-11	9
4				***CDK:DDK:Mcm10:pre-replicative complex***	5.069e^-11	9
4				***Mcm10:pre-replicative complex***	9.900e^-10	8
4				***Cdt1 displaced from the pre-replicative complex***	9.900e^-10	8
4				***Unwinding of DNA***	1.499e^-9	7
4				***Unwinding complex at replication fork***	1.499e^-9	7
4				Cdc20:phospho-APC/C mediated degradation of Cyclin A	5.470e^-9	12
4				***Degradation multiubiquitinated Cyclin A***	5.470e^-9	12
4				***Orc1 is phosphorylated by Cyclin A/CDK2***	8.830e^-9	8
4				***Pre-replicative complex(Orc1-minus)***	1.750e^-8	7
4				***RSF Complex binds the centromere***	2.070e^-8	8
4				Deposition of new CENPA-containing nucleosomes at the centro	2.070e^-8	8
4				***Mcm10:active pre-replicative complex***	2.619e^-8	7
4				***Polymerase switching***	2.619e^-8	7
4				***Removal of the Flap Intermediate***	2.619e^-8	7
4				***Polymerase switching on the C-strand of the telomere***	2.619e^-8	7
4				***Pre-replicative complex***	2.619e^-8	7
4				***RFC Heteropentamer:RNA primer-DNA primer:origin duplex***	5.589e^-8	6
4				***RFC dissociates after sliding clamp formation***	1.270e^-7	6
4				***Loading of PCNA-sliding clamp formation***	1.270e^-7	6
4				***RFC Heteropentamer : RNA primer-DNA primer: origin duple:PCNA h***	1.270e^-7	6
4				***CDT1 association with the CDC6:ORC:origin complex***	2.010e^-7	10
4				Deposition of New CENPA-containing nucleosomes at the centro	2.949e^-7	7
4				***Centromeric chromatin: New CENPA nucleosome:Mis18: HJURP com***	2.949e^-7	7
4				***Centromeric chromatin : CENPH-1:Mis18:HJURP:CENPA complex***	2.949e^-7	7
4				***Regulation of APC/C activators between G1/S and early anaphase***	4.379e^-7	7
4				***G0 and early G1***	6.360e^-7	7
4				***Removal of RNA primer and dissociation of RPA and Dna2 active pre***	8.099e^-7	6
4				***Processive complex: Okazaki fragment:Flap:RPA heterotrimer:dna2***	8.099e^-7	6
4				Degradation of multiubiquitinated cell cycle proteins	1.036e^-6	10
4				***APC/C:Cdh1 mediated degradation of Cdc20 and other***	1.190e^-6	10
4				APC/C:Cdh1 targeted proteins in late mitosis/early G1	2.210e^-6	7
4				***Mcm2-7 is phosphorylated by DDK***	2.210e^-6	5
5	Cytokine-cytokine receptor interaction	8.689e^-10	11	No match		

EXPERIMENT: E-GEOD-38836, Upregulated Canonical Pathways

Maximum stable clusters : 7

Functional enrichment p -value <10^{-5}

cluster	KEGG pathway	p-value	# of proteins	Reactome pathway	p-value	# of proteins
1	Neuroactive ligand-receptor interactior	8.47E-10	10	No Match		
1	Calcium signaling pathway	3.86E-06	6			
2	Neuroactive ligand-receptor interactior	4.88E-22	26	G alpha (i) signalling events	2.23E-15	17
2	Focal adhesion	6.66E-14	17	The Ligand:GPCR:Gi complex dissociates	2.67E-15	16
2	ECM-receptor interaction	5.52E-10	10	***The Ligand:GPCR:Gs complex dissociates***	1.67E-08	8
2	Retinol metabolism	4.15E-06	6	***G alpha (s) signalling events***	1.95E-08	9

2				The Ligand:GPCR:Gq complex dissociates	2.72E-06	8
2				G alpha (q) signalling events	6.16E-06	8
3	Neuroactive ligand-receptor interactior	2.57E-26	30	No Match		
3	Glutamatergic synapse	2.39E-10	12			
3	***Glycine, serine and threonine metabolism***	4.00E-09	7			
3	Cholinergic synapse	1.45E-08	10			
3	Calcium signaling pathway	9.66E-08	11			
3	***Pathways in cancer***	1.20E-06	13			
3	Focal adhesion	2.94E-06	10			
3	ECM-receptor interaction	4.17E-06	7			
3	Pancreatic secretion	8.74E-06	7			
4	Glutamatergic synapse	1.89E-09	8			
4	Vascular smooth muscle contraction	2.44E-08	7			
4	Gastric acid secretion	4.23E-08	6			
4	***GnRH signaling pathway***	2.76E-07	6			
4	Cholinergic synapse	6.14E-07	6			
4	***Salivary secretion***	2.75E-06	5			
4	Pancreatic secretion	6.42E-06	5			
5	Vascular smooth muscle contraction	1.31E-10	8	No match		
6	Neuroactive ligand-receptor interactior	6.22E-22	24	G alpha (q) signalling events	3.77E-10	11
6	Calcium signaling pathway	4.95E-09	11	***Activation of voltage gated Potassium channels***	4.14E-09	7
6	***Steroid hormone biosynthesis***	2.56E-08	7	***Octamer of Voltage gated K+ channels***	4.14E-09	7
6	Gastric acid secretion	1.77E-07	7	The Ligand:GPCR:Gq complex dissociates	3.52E-08	9
6	Retinol metabolism	1.15E-06	6	G alpha (i) signalling events	3.89E-07	9
6	***Drug metabolism - cytochrome P450***	3.19E-06	6	The Ligand:GPCR:Gi complex dissociates	1.19E-06	8
6	Cholinergic synapse	3.74E-06	7	***OR - G Protein Trimer Complex***	***2.82E-05***	***10***
6	Vascular smooth muscle contraction	3.97E-06	7	***Olfactory Receptor - G Protein olfactory trimer complex formation***	***2.82E-05***	***10***
6	Glutamatergic synapse	8.22E-06	7			
6	***Olfactory transduction***	***1.14E-05***	***11***			
7	No match			No match		

EXPERIMENT: E-GEOD-38836, Downregulated Canonical Pathways

Maximum stable clusters : 5

Functional enrichment p -value <10^{-5}

cluster	KEGG pathway	p-value	# of proteins	Reactome pathway	p-value	# of proteins
1	Jak-STAT signaling pathway	1.75E-11	9	None		
1	Cytokine-cytokine receptor interaction	7.91E-11	10			
1	Natural killer cell mediated cytotoxicity	2.03E-07	6			
1	***Systemic lupus erythematosus***	1.42E-06	5			
1	Measles	6.02E-06	5			
2	***Ribosome***	2.72E-19	17	GTP hydrolysis and joining of the 60S ribosomal subunit	3.02E-32	26
2	Spliceosome	9.90E-18	18	L13a-mediated translational silencing of Ceruloplasmin expressi	1.11E-30	25
2	T cell receptor signaling pathway	7.32E-15	15	Formation of a pool of free 40S subunits	1.50E-28	23
2	Natural killer cell mediated cytotoxicity	2.57E-12	14	SRP-dependent cotranslational protein targeting to membrane	2.96E-27	23
2	***RNA transport***	2.17E-10	13	Translated mRNA Complex with Premature Termination Codon I	1.61E-26	22
2	Measles	1.20E-06	9	80S:Met-tRNAi:mRNA:eIF5B:GTP	1.95E-26	21
2	***Renal cell carcinoma***	1.33E-06	7	eIF5B:GTP is hydrolyzed and released	1.95E-26	21
2	Cytokine-cytokine receptor interaction	1.55E-06	12	The 60S subunit joins the translation initiation complex	1.95E-26	21
2	B cell receptor signaling pathway	1.97E-06	7	Signal peptide cleavage from ribosome-associated nascent prote	2.09E-26	22
2	Chemokine signaling pathway	2.51E-06	10	eRF3-GDP:eRF1:80S Ribosome:mRNA:peptidyl-tRNA Complex	2.63E-26	21
2	***Neurotrophin signaling pathway***	7.13E-06	8	eRF3-GTP:eRF1:80S Ribosome:mRNA:peptidyl-tRNA Complex	2.63E-26	21
2	***Regulation of actin cytoskeleton***	7.23E-06	10	GTP Hydrolysis by eRF3 bound to the eRF1:mRNA:polypeptide:8	2.63E-26	21
2				Translocation of ribosome by 3 bases in the 3' direction	2.63E-26	21
2				eRF3-GDP:eRF1:80S Ribosome:mRNA:tRNA Complex	2.63E-26	21
2				Peptide chain elongation	2.63E-26	21
2				Eukaryotic Translation Termination	2.63E-26	21
2				Polypeptide release from the eRF3-GDP:eRF1:mRNA:80S Riboso	2.63E-26	21
2				Translocation of signal-containing nascent peptide to Endoplasn	3.50E-26	22
2				SMG1:Phosphorylated UPF1:EJC:Translated mRNP	4.52E-26	22
2				SMG1 Phosphorylates UPF1 (Enhanced by Exon Junction Comple	4.52E-26	22
2				SMG1:UPF1:EJC:Translated mRNP	4.52E-26	22
2				SRP:polypeptide+signal:ribosome	6.26E-26	21
2				Translated mRNA Complex with Premature Termination Codon I	8.28E-26	21
2				Phosphorylated UPF1:SMG5:SMG7:SMG6:PP2A:Translated mRN	9.48E-26	22
2				SMG6 Cleaves mRNA with Premature Termination Codon	9.48E-26	22
2				UPF1:eRF3 Complex on Translated mRNA	1.09E-25	21
2				SRP receptor:SRP:ribosome:polypeptide+signal	1.09E-25	21
2				Nonsense Mediated Decay Enhanced by the Exon Junction Com	1.94E-25	22
2				Phosphorylated UPF1 Recruits SMG5, SMG7, SMG6, and PP2A	1.94E-25	22

2				cleaved polypeptide:Translocon	2.45E-25	21
2				polypeptide+signal:Translocon	2.45E-25	21
2				80S Ribosome:mRNA:peptidyl-tRNA with elongating peptide	7.15E-25	20
2				Peptide transfer from P-site tRNA to the A-site tRNA	7.15E-25	20
2				Elongation complex with growing peptide chain	7.15E-25	20
2				80S ribosome	7.15E-25	20
2				membrane-bound ribosome:mRNA:cleaved polypeptide	7.15E-25	20
2				80S:Met-tRNAi:mRNA:aminoacyl-tRNA	7.15E-25	20
2				ribosome:mRNA:polypeptide+signal	7.15E-25	20
2				80S:Met-tRNAi:mRNA	7.15E-25	20
2				membrane-bound ribosome:mRNA:polypeptide+signal	7.15E-25	20
2				Hydrolysis of eEF1A:GTP	9.51E-25	20
2				80S:aminoacyl tRNA:mRNA:eEF1A:GTP	9.51E-25	20
2				***60s ribosomal complex lacking L13a subunit***	1.49E-20	15
2				***60S ribosomal complex***	2.14E-20	15
2				mRNA Splicing - Major Pathway	8.63E-18	17
2				Spliceosomal B Complex	1.61E-17	16
2				Formation of the Spliceosomal B Complex	1.61E-17	16
2				Formation of an intermediate Spliceosomal C complex	1.38E-16	16
2				Exon Junction Complex	1.63E-16	16
2				Cleavage at the 3'-Splice Site and Exon Ligation	1.63E-16	16
2				Spliceosomal Intermediate C Complex	2.52E-15	15
2				Spliceosomal Active C Complex	2.52E-15	15
2				Spliceosomal A Complex	2.60E-15	14
2				Spliceosomal active C complex with lariat containing, 5'-end clea	2.95E-15	15
2				Lariat Formation and 5'-Splice Site Cleavage	2.95E-15	15
2				***48S complex***	7.59E-12	10
2				***43S:mRNA:eIF4F:eIF4B:eIF4H***	7.59E-12	10
2				***Ribosomal scanning***	7.59E-12	10
2				***Ribosomal scanning and start codon recognition***	9.38E-12	10
2				***eIF2:GTP is hydrolyzed, eIFs are released***	9.38E-12	10
2				***Formation of translation initiation complexes yielding circularized C***	9.38E-12	10
2				***43S: Ceruloplasmin mRNA:eIF4F:eIF4B:eIF4H:PABP***	9.38E-12	10
2				***phospho-L13a associated wth the 3' UTR GAIT element of ceruloplas***	1.15E-11	10
2				Formation of the Spliceosomal E complex	7.31E-11	10
2				Spliceosomal E Complex	7.31E-11	10
2				mRNA Splicing - Minor Pathway	8.08E-10	8
2				ATAC B Complex	8.08E-10	8
2				ATAC C Complex with lariat containing 5'-end cleaved mRNA	8.08E-10	8
2				ATAC C Complex	8.08E-10	8
2				Formation of AT-AC C complex	8.08E-10	8
2				ATAC spliceosome mediated 3' splice site cleavage, exon ligatior	8.08E-10	8
2				***40S:eIF3:eIF1A***	1.24E-09	8
2				***Formation of the ternary complex, and subsequently, the 43S compl***	2.25E-09	8
2				***Formation of the 43S pre-initiation complex***	2.25E-09	8
2				***43S complex***	2.25E-09	8
2				intron-containing complex	1.75E-08	7
2				ATAC A Complex	1.65E-07	6
2				***U2 snRNP***	2.64E-07	5
2				RNA Polymerase II Pre-transcription Events	2.67E-07	7
2				Docking of the TAP:EJC Complex with the NPC	2.16E-06	6
2				HIV-1 promoter:TFIID:TFIIA:TFIIB:Pol II:TFIIF complex*	2.34E-06	5
2				pol II promoter:TFIID:TFIIA:TFIIB:Pol II:TFIIF complex	2.34E-06	5
2				Transport of Mature mRNA derived from an Intron-Containing T	2.45E-06	6
2				pol II promoter:TFIID:TFIIA:TFIIB:Pol II:TFIIF:TFIIE complex	3.40E-06	5
2				***40S ribosomal complex***	4.05E-06	5
2				***40S:Met-tRNAi:mRNA***	4.05E-06	5
2				***capped, methylated pre-mRNP:CBC complex***	5.65E-06	5
3	Spliceosome	8.96E-18	18	Cdc20:Phospho-APC/C mediated degradation of Cyclin A	3.32E-18	15
3	MAPK signaling pathway	4.93E-13	19	Degradation multiubiquitinated Cyclin A	3.32E-18	15
3	Cell cycle	9.70E-13	14	Degradation of beta-catenin by the destruction complex	4.13E-18	14
3	Proteasome	1.41E-12	10	Degradation of ubiquitinated -beta catenin by the proteasome	4.13E-18	14
3	Huntington's disease	2.44E-10	14	Degradation of multiubiquitinated Cdh1	2.27E-17	14
3	***Hepatitis C***	6.19E-10	12	Autodegradation of Cdh1 by Cdh1:APC/C	2.27E-17	14
3	***TGF-beta signaling pathway***	1.20E-09	10	APC/C:Cdh1-mediated degradation of Skp2	6.33E-17	14
3	Natural killer cell mediated cytotoxicity	9.23E-08	10	Degradation of multiubiquitinated Securin	6.33E-17	14
3	***RNA polymerase***	1.04E-07	6	APC/C:Cdc20 mediated degradation of Securin	6.33E-17	14
3	Measles	1.07E-07	10	Degradation of ubiquitinated p27/p21 by the 26S proteasome	8.27E-17	13
3	***Ubiquitin mediated proteolysis***	1.33E-07	10	SCF(Skp2)-mediated degradation of p27/p21	8.27E-17	13
3	***Oocyte meiosis***	2.19E-07	9	SCF-mediated degradation of Emi1	1.48E-16	13
3	Cytosolic DNA-sensing pathway	4.80E-07	7	SCF-beta-TrCP mediated degradation of Emi1	1.48E-16	13
3	Parkinson's disease	8.81E-07	9	Degradation of multiubiquitinated cell cycle proteins	1.64E-16	14
3	Chagas disease (American trypanosomi	1.54E-06	8	APC/C:Cdh1 mediated degradation of Cdc20 and other APC/C:C	2.06E-16	14
3	Wnt signaling pathway	3.18E-06	9	Regulation of activated PAK-2p34 by proteasome mediated degr	2.20E-15	12
3	***mRNA surveillance pathway***	4.21E-06	7	Proteasome mediated degradation of COP1	2.20E-15	12
3	Osteoclast differentiation	6.44E-06	8	Proteolytic degradation of ubiquitinated-Cdc25A	2.20E-15	12

3	Alzheimer's disease	7.05E-06	9	Proteasome mediated degradation of PAK-2p34	2.20E-15	12
3	Oxidative phosphorylation	9.76E-06	8	Autodegradation of the E3 ubiquitin ligase COP1	3.82E-15	12
3				Ubiquitin Mediated Degradation of Phosphorylated Cdc25A	3.82E-15	12
3				Destabilization of mRNA by AUF1 (hnRNP D0)	4.99E-15	12
3				Antigen processing: Ubiquitination & Proteasome degradation	6.49E-15	12
3				26S proteasome	9.74E-15	11
3				CDT1 association with the CDC6:ORC:origin complex	1.74E-14	12
3				Destruction of AUF1 and mRNA	2.44E-14	11
3				Proteasomal clevage of exogenous antigen	2.44E-14	11
3				Cross-presentation of soluble exogenous antigens (endosomes)	5.66E-14	11
3				Proteasomal cleavage of substrate	7.38E-14	11
3				26S proteosome degrades ODC holoenzyme complex	7.38E-14	11
3				Ubiquitinated Cdc6 is degraded by the proteasome	9.57E-14	11
3				Ubiquitinated Orc1 is degraded by the proteasome	9.57E-14	11
3				Proteasome mediated degradation of Cyclin D1	9.57E-14	11
3				Ubiquitinated geminin is degraded by the proteasome	9.57E-14	11
3				Ubiquitin-dependent degradation of Cyclin D1	1.23E-13	11
3				CDK-mediated phosphorylation and removal of Cdc6	1.23E-13	11
3				Orc1 removal from chromatin	1.49E-13	12
3				Activation of NF-kappaB in B Cells	5.01E-13	11
3				Spliceosomal Intermediate C Complex	1.20E-12	13
3				Spliceosomal Active C Complex	1.20E-12	13
3				Spliceosomal active C complex with lariat containing, 5'-end clea	1.37E-12	13
3				Lariat Formation and 5'-Splice Site Cleavage	1.37E-12	13
3				Formation of an intermediate Spliceosomal C complex	1.57E-12	13
3				Spliceosomal A Complex	1.72E-12	12
3				Exon Junction Complex	1.79E-12	13
3				Cleavage at the 3'-Splice Site and Exon Ligation	1.79E-12	13
3				mRNA Splicing - Major Pathway	2.31E-12	13
3				ER-Phagosome pathway	5.17E-12	11
3				Spliceosomal B Complex	6.48E-12	12
3				Formation of the Spliceosomal B Complex	6.48E-12	12
3				Formation of the Spliceosomal E complex	6.92E-11	10
3				Spliceosomal E Complex	6.92E-11	10
3				**TRAF6 mediated NF-kB activation**	7.29E-10	6
3				**Interleukin-1 signaling**	1.11E-08	7
3				ATAC A Complex	1.60E-07	6
3				***mRNA 3'-end processing***	2.86E-07	6
3				***Cleavage of mRNA at the 3'-end***	2.86E-07	6
3				***Ligated exon containing complex***	2.86E-07	6
3				mRNA Splicing - Minor Pathway	6.70E-07	6
3				ATAC B Complex	6.70E-07	6
3				ATAC C Complex with lariat containing 5'-end cleaved mRNA	6.70E-07	6
3				ATAC C Complex	6.70E-07	6
3				Formation of AT-AC C complex	6.70E-07	6
3				ATAC spliceosome mediated 3' splice site cleavage, exon ligatior	6.70E-07	6
3				***3'-polyadenylated, capped mRNA complex***	7.68E-07	5
3				***TAP:3'-polyadenylated, capped mRNA complex***	9.75E-07	5
3				Docking of the TAP:EJC Complex with the NPC	9.75E-07	5
3				***Prefoldin mediated transfer of substrate to CCT/TriC***	1.22E-06	5
3				***Actin/tubulin:prefoldin complex associates with CCT/TriC***	1.22E-06	5
3				Phosphorylated UPF1:SMG5:SMG7:SMG6:PP2A:Translated mRN	1.43E-06	8
3				SMG6 Cleaves mRNA with Premature Termination Codon	1.43E-06	8
3				Nonsense Mediated Decay Enhanced by the Exon Junction Com	1.79E-06	8
3				Phosphorylated UPF1 Recruits SMG5, SMG7, SMG6, and PP2A	1.79E-06	8
3				***mRNA polyadenylation***	2.28E-06	5
3				***3' end cleaved, ligated exon containing complex***	2.28E-06	5
3				Transport of Mature mRNA derived from an Intron-Containing T	2.37E-06	6
3				Respiratory electron transport	2.50E-06	7
3				Formation of the Early Elongation Complex	2.76E-06	5
3				Formation of the HIV-1 Early Elongation Complex	2.76E-06	5
3				Cyclin D associated events in G1	2.76E-06	5
3				RNA Polymerase II Pre-transcription Events	4.80E-06	6
3				***HP subcomplex***	7.50E-06	5
4	Natural killer cell mediated cytotoxicity	1.67E-15	16	SRP-dependent cotranslational protein targeting to membrane	4.81E-13	13
4	Huntington's disease	5.38E-12	15	Signal peptide cleavage from ribosome-associated nascent prote	4.28E-12	12
4	Oxidative phosphorylation	1.95E-10	12	ER-Phagosome pathway	5.32E-11	10
4	Proteasome	2.68E-08	7	Translocation of signal-containing nascent peptide to Endoplasn	1.14E-10	11
4	Parkinson's disease	3.33E-08	10	RNA Polymerase II Pre-transcription Events	2.07E-10	9
4	Alzheimer's disease	3.42E-08	11	cleaved polypeptide:Translocon	9.91E-10	10
4	***Protein export***	4.87E-07	5	polypeptide+signal:Translocon	9.91E-10	10
4	Cytokine-cytokine receptor interaction	5.49E-07	12	Orc1 removal from chromatin	1.16E-09	9
4	***Chronic myeloid leukemia***	7.65E-07	7	APC/C:Cdh1 mediated degradation of Cdc20 and other APC/C:C	1.53E-09	9
4	Jak-STAT signaling pathway	2.08E-06	9	Degradation of ubiquitinated p27/p21 by the 26S proteasome	2.56E-09	8
4	Spliceosome	3.92E-06	8	SCF(Skp2)-mediated degradation of p27/p21	2.56E-09	8
4	Cytosolic DNA-sensing pathway	4.78E-06	6	Spliceosomal Intermediate C Complex	2.89E-09	10

4	Measles	5.57E-06	8	Spliceosomal Active C Complex	2.89E-09	10
4				Spliceosomal active C complex with lariat containing, 5'-end clea	3.19E-09	10
4				Lariat Formation and 5'-Splice Site Cleavage	3.19E-09	10
4				Formation of an intermediate Spliceosomal C complex	3.52E-09	10
4				Exon Junction Complex	3.89E-09	10
4				Cleavage at the 3'-Splice Site and Exon Ligation	3.89E-09	10
4				Respiratory electron transport	4.27E-09	9
4				mRNA Splicing - Major Pathway	4.71E-09	10
4				Activation of NF-kappaB in B Cells	5.73E-09	8
4				***Early elongation complex with hyperphosphorylated Pol II CTD***	7.25E-09	6
4				***HIV-1 early elongation complex with hyperphosphorylated Pol II CTD***	7.25E-09	6
4				***RNA Polymerase II Transcription Elongation***	7.26E-09	7
4				***Recruitment of elongation factors to form HIV-1 elongation complex***	7.26E-09	7
4				***Addition of nucleotides leads to transcript elongation***	7.26E-09	7
4				***Elongation complex***	7.26E-09	7
4				***HIV-1 elongation complex***	7.26E-09	7
4				***Formation of RNA Pol II elongation complex***	1.08E-08	7
4				***Formation of HIV-1 elongation complex in the absence of HIV-1 Tat***	1.08E-08	7
4				***Hyperphosphorylation (Ser2) of RNA Pol II CTD by P-TEFb complex***	1.33E-08	6
4				APC/C:Cdh1-mediated degradation of Skp2	1.77E-08	8
4				Spliceosomal B Complex	1.79E-08	9
4				Formation of the Spliceosomal B Complex	1.79E-08	9
4				Destruction of AUF1 and mRNA	2.26E-08	7
4				Proteasomal clevage of exogenous antigen	2.26E-08	7
4				26S proteasome	2.26E-08	7
4				Degradation of multiubiquitinated cell cycle proteins	2.94E-08	8
4				***Formation of transcription-coupled NER (TC-NER) repair complex***	2.98E-08	6
4				***Dual incision reaction in TC-NER***	2.98E-08	6
4				***Assembly of repair proteins at the site of Pol II blockage***	2.98E-08	6
4				***Displacement of stalled Pol II from the lesion site***	2.98E-08	6
4				Cross-presentation of soluble exogenous antigens (endosomes)	3.73E-08	7
4				Proteasomal cleavage of substrate	4.37E-08	7
4				26S proteosome degrades ODC holoenzyme complex	4.37E-08	7
4				***HIV-1 arrested processive elongation complex***	4.82E-08	6
4				***HIV-1 paused processive elongation complex***	4.82E-08	6
4				***2-4 nt.backtracking of Pol II complex on the template leading to elor***	4.82E-08	6
4				***Resumption of elongation after recovery from pausing***	4.82E-08	6
4				***Abortive termination of HIV-1 elongation after arrest***	4.82E-08	6
4				***Elongation arrest and recovery***	4.82E-08	6
4				***HIV-1 elongation arrest and recovery***	4.82E-08	6
4				***Pausing and recovery of HIV-1 elongation***	4.82E-08	6
4				***Processive elongation complex***	4.82E-08	6
4				***Arrested processive elongation complex***	4.82E-08	6
4				***Paused processive elongation complex***	4.82E-08	6
4				***HIV-1 processive elongation complex***	4.82E-08	6
4				***Resumption of elongation of HIV-1 transcript after recovery from pa***	4.82E-08	6
4				***HIV-1 aborted elongation complex after arrest***	4.82E-08	6
4				***Abortive termination of elongation after arrest***	4.82E-08	6
4				***Elongating transcript encounters a lesion in the template***	4.82E-08	6
4				***Aborted elongation complex after arrest***	4.82E-08	6
4				***Elongation complex prior to separation***	4.82E-08	6
4				***Elongation complex with separated and uncleaved transcript***	4.82E-08	6
4				***2-4 nt.backtracking of Pol II complex on the HIV-1 template leading***	4.82E-08	6
4				Proteasome mediated degradation of Cyclin D1	5.11E-08	7
4				Regulation of activated PAK-2p34 by proteasome mediated degr	5.11E-08	7
4				Proteasome mediated degradation of COP1	5.11E-08	7
4				Proteolytic degradation of ubiquitinated-Cdc25A	5.11E-08	7
4				Ubiquitinated geminin is degraded by the proteasome	5.11E-08	7
4				Ubiquitinated Cdc6 is degraded by the proteasome	5.11E-08	7
4				Ubiquitinated Orc1 is degraded by the proteasome	5.11E-08	7
4				Proteasome mediated degradation of PAK-2p34	5.11E-08	7
4				Ubiquitin-dependent degradation of Cyclin D1	5.94E-08	7
4				CDK-mediated phosphorylation and removal of Cdc6	5.94E-08	7
4				Autodegradation of the E3 ubiquitin ligase COP1	6.89E-08	7
4				Ubiquitin Mediated Degradation of Phosphorylated Cdc25A	6.89E-08	7
4				Destabilization of mRNA by AUF1 (hnRNP D0)	7.96E-08	7
4				Antigen processing: Ubiquitination & Proteasome degradation	9.17E-08	7
4				SCF-mediated degradation of Emi1	9.17E-08	7
4				SCF-beta-TrCP mediated degradation of Emi1	9.17E-08	7
4				Degradation of beta-catenin by the destruction complex	1.05E-07	7
4				Degradation of ubiquitinated -beta catenin by the proteasome	1.05E-07	7
4				Spliceosomal A Complex	1.23E-07	8
4				CDT1 association with the CDC6:ORC:origin complex	1.57E-07	7
4				SRP receptor:SRP:ribosome:polypeptide+signal	2.19E-07	8
4				***Addition of Nucleotides 5 through 9 on the growing Transcript***	2.37E-07	6
4				***HIV-1 transcription complex containing 4 nucleotide long transcript***	2.37E-07	6
4				***Addition of nucleotides 5 through 9 on the growing HIV-1 transcript***	2.37E-07	6

4	***pol II transcription complex containing 4 nucleotide long transcript***	2.37E-07	6
4	***RNA Polymerase II Transcription Initiation***	2.82E-07	6
4	***RNA Polymerase II Transcription Pre-Initiation And Promoter Openir***	2.82E-07	6
4	***RNA Polymerase II Promoter Opening: First Transition***	2.82E-07	6
4	Newly Formed Phosphodiester Bond Stabilized and PPi Releasec	2.82E-07	6
4	***RNA Polymerase II Promoter Escape***	2.82E-07	6
4	***Pol II Promoter Escape Complex***	2.82E-07	6
4	***HIV-1 Transcription Initiation***	2.82E-07	6
4	***Transcription of the HIV genome***	2.82E-07	6
4	***Addition of the third nucleotide on the nascent HIV-1 transcript***	2.82E-07	6
4	***Addition of the fourth nucleotide on the nascent HIV-1 transcript: Se***	2.82E-07	6
4	***Fall Back to Closed Pre-initiation Complex***	2.82E-07	6
4	***Nucleophillic attack by 3'-hydroxyl oxygen of nascent HIV-1 transcrip***	2.82E-07	6
4	Newly formed phosphodiester bond stabilized and PPi released	2.82E-07	6
4	***NTP binds active site of RNA Polymerase II in HIV-1 open pre-initiatic***	2.82E-07	6
4	***Addition of the fourth nucleotide on the Nascent Transcript: Second***	2.82E-07	6
4	***HIV-1 transcription complex containing 3 nucleotide long transcript***	2.82E-07	6
4	***HIV-1 initiation complex with phosphodiester-PPi intermediate***	2.82E-07	6
4	***HIV-1 open pre-initiation complex***	2.82E-07	6
4	***HIV-1 Promoter Escape Complex***	2.82E-07	6
4	***RNA Polymerase II HIV-1 Promoter Escape***	2.82E-07	6
4	***HIV-1 closed pre-initiation complex***	2.82E-07	6
4	***pol II transcription complex***	2.82E-07	6
4	***NTP Binds Active Site of RNA Polymerase II***	2.82E-07	6
4	***Nucleophillic Attack by 3'-hydroxyl Oxygen of nascent transcript on t***	2.82E-07	6
4	***Addition of the third nucleotide on the nascent transcript***	2.82E-07	6
4	***HIV-1 initiation complex***	2.82E-07	6
4	***HIV-1 Promoter Opening: First Transition***	2.82E-07	6
4	***HIV-1 transcription complex***	2.82E-07	6
4	***pol II transcription complex containing 3 Nucleotide long transcript***	2.82E-07	6
4	***Pol II Initiation complex with phosphodiester-PPi intermediate***	2.82E-07	6
4	***pol II closed pre-initiation complex***	2.82E-07	6
4	***Pol II initiation complex***	2.82E-07	6
4	***pol II open pre-initiation complex***	2.82E-07	6
4	Formation of a pool of free 40S subunits	3.13E-07	8
4	Degradation of multiubiquitinated Securin	3.64E-07	7
4	APC/C:Cdc20 mediated degradation of Securin	3.64E-07	7
4	mRNA Splicing - Minor Pathway	3.91E-07	6
4	ATAC B Complex	3.91E-07	6
4	ATAC C Complex with lariat containing 5'-end cleaved mRNA	3.91E-07	6
4	ATAC C Complex	3.91E-07	6
4	Formation of AT-AC C complex	3.91E-07	6
4	ATAC spliceosome mediated 3' splice site cleavage, exon ligatior	3.91E-07	6
4	Translated mRNA Complex with Premature Termination Codon I	4.05E-07	8
4	Cdc20:Phospho-APC/C mediated degradation of Cyclin A	5.06E-07	7
4	Degradation multiubiquitinated Cyclin A	5.06E-07	7
4	SMG1:Phosphorylated UPF1:EJC:Translated mRNP	5.62E-07	8
4	SMG1 Phosphorylates UPF1 (Enhanced by Exon Junction Comple	5.62E-07	8
4	SMG1:UPF1:EJC:Translated mRNP	5.62E-07	8
4	L13a-mediated translational silencing of Ceruloplasmin expressi	7.12E-07	8
4	Phosphorylated UPF1:SMG5:SMG7:SMG6:PP2A:Translated mRN	7.12E-07	8
4	SMG6 Cleaves mRNA with Premature Termination Codon	7.12E-07	8
4	GTP hydrolysis and joining of the 60S ribosomal subunit	7.69E-07	8
4	***Complex I - NADH:Ubiquinone oxidoreductase***	8.20E-07	6
4	***NADH enters the respiratory chain at Complex I***	8.20E-07	6
4	Nonsense Mediated Decay Enhanced by the Exon Junction Comp	8.96E-07	8
4	Phosphorylated UPF1 Recruits SMG5, SMG7, SMG6, and PP2A	8.96E-07	8
4	HIV-1 promoter:TFIID:TFIIA:TFIIB:Pol II:TFIIF complex*	1.45E-06	5
4	pol II promoter:TFIID:TFIIA:TFIIB:Pol II:TFIIF complex	1.45E-06	5
4	Formation of the Early Elongation Complex	1.75E-06	5
4	Formation of the HIV-1 Early Elongation Complex	1.75E-06	5
4	Cyclin D associated events in G1	1.75E-06	5
4	80S Ribosome:mRNA:peptidyl-tRNA with elongating peptide	1.77E-06	7
4	Peptide transfer from P-site tRNA to the A-site tRNA	1.77E-06	7
4	Elongation complex with growing peptide chain	1.77E-06	7
4	80S ribosome	1.77E-06	7
4	membrane-bound ribosome:mRNA:cleaved polypeptide	1.77E-06	7
4	80S:Met-tRNAi:mRNA:aminoacyl-tRNA	1.77E-06	7
4	ribosome:mRNA:polypeptide+signal	1.77E-06	7
4	80S:Met-tRNAi:mRNA	1.77E-06	7
4	membrane-bound ribosome:mRNA:polypeptide+signal	1.77E-06	7
4	Hydrolysis of eEF1A:GTP	1.93E-06	7
4	80S:Met-tRNAi:mRNA:eIF5B:GTP	1.93E-06	7
4	80S:aminoacyl tRNA:mRNA:eEF1A:GTP	1.93E-06	7
4	eIF5B:GTP is hydrolyzed and released	1.93E-06	7
4	The 60S subunit joins the translation initiation complex	1.93E-06	7
4	eRF3-GDP:eRF1:80S Ribosome:mRNA:peptidyl-tRNA Complex	2.10E-06	7

4				eRF3-GTP:eRF1:80S Ribosome:mRNA:peptidyl-tRNA Complex	2.10E-06	7
4				GTP Hydrolysis by eRF3 bound to the eRF1:mRNA:polypeptide:8	2.10E-06	7
4				Translocation of ribosome by 3 bases in the 3' direction	2.10E-06	7
4				eRF3-GDP:eRF1:80S Ribosome:mRNA:tRNA Complex	2.10E-06	7
4				Peptide chain elongation	2.10E-06	7
4				Eukaryotic Translation Termination	2.10E-06	7
4				Polypeptide release from the eRF3-GDP:eRF1:mRNA:80S Riboso	2.10E-06	7
4				pol II promoter:TFIID:TFIIA:TFIIB:Pol II:TFIIF:TFIIE complex	2.11E-06	5
4				SRP:polypeptide+signal:ribosome	2.70E-06	7
4				Translated mRNA Complex with Premature Termination Codon I	2.93E-06	7
4				ATAC A Complex	2.98E-06	5
4				UPF1:eRF3 Complex on Translated mRNA	3.17E-06	7
4				Degradation of multiubiquitinated Cdh1	4.32E-06	6
4				Autodegradation of Cdh1 by Cdh1:APC/C	4.32E-06	6
4				Formation of the Spliceosomal E complex	6.40E-06	6
4				Spliceosomal E Complex	6.40E-06	6
4				Interferon alpha/beta signaling	7.03E-06	6
4				intron-containing complex	7.37E-06	5
5	T cell receptor signaling pathway	2.82E-13	12	Interferon alpha/beta signaling	9.22E-07	6
5	MAPK signaling pathway	1.29E-08	12	Respiratory electron transport	2.60E-06	6
5	Osteoclast differentiation	1.40E-08	9	***Expression of IFN-induced genes***	3.59E-06	5
5	B cell receptor signaling pathway	9.49E-08	7			
5	Cytokine-cytokine receptor interaction	1.16E-07	11			
5	***Pathogenic Escherichia coli infection***	2.89E-07	6			
5	Chagas disease (American trypanosomi	9.15E-07	7			
5	Jak-STAT signaling pathway	1.38E-06	8			
5	Cell cycle	3.12E-06	7			
5	Chemokine signaling pathway	5.02E-06	8			
5	***Toxoplasmosis***	5.31E-06	7			
5	***Apoptosis***	5.41E-06	6			

EXPERIMENT: E-GEOD-43582, Upregulated Canonical Pathways

Maximum stable clusters : 5

Functional enrichment p -value <10^{-5}

	KEGG pathway	p-value	# of proteins	Reactome pathway	p-value	# of proteins
1	Olfactory transduction	7.26E-19	37	Olfactory Receptor - G Protein olfactory trimer complex formati	2.18E-13	29
1	Cell cycle	3.33E-16	21	OR - G Protein Trimer Complex	2.18E-13	29
1	***DNA replication***	1.99E-13	12	DNA polymerase alpha:primase binds at the origin	2.44E-13	11
1	Oocyte meiosis	1.45E-11	16	Activation of the pre-replicative complex	3.88E-13	11
1	Progesterone-mediated oocyte matura	4.38E-11	14	Spliceosomal Intermediate C Complex	3.61E-12	16
1	Metabolic pathways	1.77E-07	42	Spliceosomal Active C Complex	3.61E-12	16
1	***Lysosome***	5.04E-07	12	Spliceosomal active C complex with lariat containing, 5'-end clea	4.24E-12	16
1	N-Glycan biosynthesis	7.05E-07	8	Lariat Formation and 5'-Splice Site Cleavage	4.24E-12	16
1	Ubiquitin mediated proteolysis	1.53E-06	12	Formation of an intermediate Spliceosomal C complex	4.97E-12	16
1	Aminoacyl-tRNA biosynthesis	3.48E-06	7	Exon Junction Complex	5.83E-12	16
1	***Selenocompound metabolism***	5.29E-06	5	Cleavage at the 3'-Splice Site and Exon Ligation	5.83E-12	16
1	Nucleotide excision repair	6.64E-06	7	mRNA Splicing - Major Pathway	7.95E-12	16
1				Spliceosomal B Complex	9.01E-12	15
1				Formation of the Spliceosomal B Complex	9.01E-12	15
1				Microtubule-bound kinetochore	1.02E-11	14
1				Kinetochore	1.02E-11	14
1				DNA polymerase epsilon binds at the origin	6.36E-11	9
1				***Orc1 removal from chromatin***	4.22E-10	12
1				***RPA:Cdc45:CDK:DDK:Mcm10:pre-replicative complex***	1.01E-09	8
1				***Unwinding of DNA***	3.13E-09	6
1				***Unwinding complex at replication fork***	3.13E-09	6
1				***intron-containing complex***	5.04E-09	9
1				Formation of the Spliceosomal E complex	4.81E-08	10
1				Spliceosomal E Complex	4.81E-08	10
1				Activation of ATR in response to replication stress	4.87E-08	8
1				***U4:U5:U6 trisnRNP complex***	6.97E-08	6
1				snRNP nuclear import and release	9.94E-08	8
1				***hnRNP proteins***	1.68E-07	6
1				***FA core complex***	2.36E-07	5
1				snRNP Assembly	4.14E-07	8
1				***Cross-presentation of soluble exogenous antigens (endosomes)***	4.97E-07	8
1				Spliceosomal A Complex	5.07E-07	10
1				***G0 and Early G1***	6.97E-07	6
1				***Proteolytic degradation of ubiquitinated-Cdc25A***	7.05E-07	8
1				***Cyclin A/B1 associated events during G2/M transition***	7.23E-07	5
1				***CDK-mediated phosphorylation and removal of Cdc6***	8.34E-07	8
1				***Ubiquitin Mediated Degradation of Phosphorylated Cdc25A***	9.83E-07	8
1				***Degradation of ubiquitinated p27/p21 by the 26S proteasome***	9.83E-07	8
1				***SCF(Skp2)-mediated degradation of p27/p21***	9.83E-07	8
1				***ER-Phagosome pathway***	9.99E-07	9

1				Degradation of multiubiquitinated cell cycle proteins	9.99E-07	9
1				APC/C:Cdh1 mediated degradation of Cdc20 and other APC/C:C	1.14E-06	9
1				***U5 snRNP***	1.78E-06	5
1				***Removal of the Flap Intermediate***	1.78E-06	5
1				***U4 ATAC:U5:U6 ATAC Complex***	1.78E-06	5
1				***26S proteasome***	2.44E-06	7
1				***Monoubiquitination of FANCI by the FA ubiquitin ligase complex***	2.63E-06	5
1				Degradation of multiubiquitinated Cdh1	3.69E-06	8
1				Autodegradation of Cdh1 by Cdh1:APC/C	3.69E-06	8
1				***Monoubiquitination of FANCD2 by the FA ubiquitin ligase complex***	3.78E-06	5
1				***Destruction of AUF1 and mRNA***	4.12E-06	7
1				***Proteasomal clevage of exogenous antigen***	4.12E-06	7
1				cNAP-1 depleted centrosome	4.21E-06	8
1				Nlp-depleted centrosome	4.21E-06	8
1				Plk1-mediated phosphorylation of Nlp	4.79E-06	8
1				Loss of C-Nap-1 from centrosomes	4.79E-06	8
1				Centrosomes containing recruited CDK11p58	4.79E-06	8
1				centrosome	4.79E-06	8
1				Centrosome associated Plk1	4.79E-06	8
1				Loss of Nlp from mitotic centrosomes	4.79E-06	8
1				Dissociation of Phospho-Nlp from the centrosome	4.79E-06	8
1				centrosome containing phosphorylated Nlp	4.79E-06	8
1				***CDK:DDK:Mcm10:pre-replicative complex***	5.29E-06	5
1				APC/C:Cdh1-mediated degradation of Skp2	6.16E-06	8
1				Degradation of multiubiquitinated Securin	6.16E-06	8
1				APC/C:Cdc20 mediated degradation of Securin	6.16E-06	8
1				***Cdc45:CDK:DDK:Mcm10:pre-replicative complex***	7.23E-06	5
1				***Proteasomal cleavage of substrate***	7.73E-06	7
1				***26S proteosome degrades ODC holoenzyme complex***	7.73E-06	7
1				Cdc20:Phospho-APC/C mediated degradation of Cyclin A	8.81E-06	8
1				Degradation multiubiquitinated Cyclin A	8.81E-06	8
1				***Regulation of activated PAK-2p34 by proteasome mediated degrada***	8.96E-06	7
1				***Proteasome mediated degradation of COP1***	8.96E-06	7
1				***Ubiquitinated Cdc6 is degraded by the proteasome***	8.96E-06	7
1				***Ubiquitinated Orc1 is degraded by the proteasome***	8.96E-06	7
1				***Proteasome mediated degradation of PAK-2p34***	8.96E-06	7
1				***Proteasome mediated degradation of Cyclin D1***	8.96E-06	7
1				***Ubiquitinated geminin is degraded by the proteasome***	8.96E-06	7
1				Activation of claspin	9.68E-06	5
1				Cdc45:CDK:DDK:Mcm10:claspin:pre-replicative complex	9.68E-06	5
1				Cdc45:CDK:DDK:Mcm10:Activated claspin:pre-replicative compl	9.68E-06	5
1				***Orc1 is phosphorylated by cyclin A/CDK2***	9.68E-06	5
1				Mature centrosomes enriched in gamma-TURC complexes	9.89E-06	8
2	***Taste transduction***	5.40E-11	10	Kinetochore	2.78E-20	18
2	***Homologous recombination***	1.83E-10	8	***Meiotic Recombination***	1.02E-15	11
2	Oocyte meiosis	9.22E-10	12	***Centromeric Chromatin:CENPH-I Complex***	2.46E-08	5
2	Progesterone-mediated oocyte matura	1.05E-08	10	***Formation of Meiotic Heteroduplex***	1.23E-07	5
2	Ubiquitin mediated proteolysis	1.13E-08	12	***Centromeric Chromatin:CENPH-I:Centromeric Nucleosome:RSF Comp***	1.23E-07	5
2	***Mismatch repair***	8.81E-08	6	***Centromeric Chromatin:CENPH-I: Mis18 Complex***	2.81E-07	5
2	***Base excision repair***	8.88E-07	6	***Cdc20:phosph-APC/C:Securin complex***	4.06E-07	5
2	p53 signaling pathway	4.66E-06	7	Inactivation of APC/C via direct inhibition of the APC/C complex	7.83E-07	5
2	Nucleotide excision repair	5.90E-06	6	MCC:APC/C complex	7.83E-07	5
2	***Measles***	7.26E-06	9	Activation of APC/C:Cdc20 by dissociation of Cdc20:phospho-AP	7.83E-07	5
2				Activation of claspin	1.05E-06	5
2				***Deposition of New CENPA-containing Nucleosomes at the Centrome***	1.05E-06	5
2				***Centromeric Chromatin: New CENPA Nucleosome:Mis18:HJURP Com***	1.05E-06	5
2				Cdc45:CDK:DDK:Mcm10:claspin:pre-replicative complex	1.05E-06	5
2				Cdc45:CDK:DDK:Mcm10:Activated claspin:pre-replicative compl	1.05E-06	5
2				Nek2A:MCC:APC/C complex	1.05E-06	5
2				Cdc2:Cyclin A:MCC:APC/C complex	1.05E-06	5
2				***Centromeric Chromatin:CENPH-I:Mis18:HJURP:CENPA Complex***	1.05E-06	5
2				Activation of ATR in response to replication stress	1.07E-06	6
2				APC/C:Cdh1-mediated degradation of Skp2	1.39E-06	5
2				***Ubiquitination of Securin by phospho-APC/C:Cdc20 complex***	1.39E-06	5
2				***multiubiquitinated Securin in complex with CDC20:phospho-APC/C***	1.39E-06	5
2				***cell cycle proteins:phospho-APC/C:Cdh1 complex***	1.39E-06	5
2				***Regulation of APC/C activators between G1/S and early anaphase***	1.39E-06	5
2				***multiubiquitinated Skp2:phospho-APC/C:Cdh1 complex***	1.39E-06	5
2				Degradation of multiubiquitinated Securin	1.39E-06	5
2				***RSF Complex Binds the Centromere.***	1.81E-06	5
2				***Kinesins***	1.81E-06	5
2				***Deposition of New CENPA-containing Nucleosomes at the Centrome***	1.81E-06	5
2				***Ubiquitination of Cyclin A by APC/C:Cdc20 complex***	2.95E-06	5
2				***Degradation of multiubiquitinated Nek2A***	2.95E-06	5
2				***Multiubiquitination of Nek2A***	2.95E-06	5
2				***Multiubiquitinated Nek2A***	2.95E-06	5

2				***APC-Cdc20 mediated degradation of Nek2A***	2.95E-06	5
2				***multiubiquitinated Cyclin A associated with MCC:APC/C complex***	2.95E-06	5
2				DNA polymerase epsilon binds at the origin	3.70E-06	5
2				Degradation of multiubiquitinated cell cycle proteins	3.70E-06	5
2				***Ubiquitination of cell cycle proteins targeted by the APC/C:Cdh1com***	3.70E-06	5
2				***multiubiquitinated cell cycle protein:APC/C:Cdh1 complex***	3.70E-06	5
2				DNA polymerase alpha:primase binds at the origin	8.27E-06	5
2				Activation of the pre-replicative complex	9.91E-06	5
3	Ubiquitin mediated proteolysis	1.61E-21	26	***Docking of the Mature intronless derived transcript derived mRNA, T***	5.80E-13	11
3	Aminoacyl-tRNA biosynthesis	2.35E-14	13	***Transport of Mature mRNA Derived from an Intronless Transcript***	8.72E-13	11
3	Metabolic pathways	3.28E-14	54	***Transport of the export-competent complex through the NPC***	1.88E-12	11
3	Cell cycle	4.13E-12	17	Spliceosomal active C complex with lariat containing, 5'-end clea	2.22E-12	16
3	***Basal transcription factors***	5.74E-11	11	Lariat Formation and 5'-Splice Site Cleavage	2.22E-12	16
3	Oocyte meiosis	9.79E-09	13	***Release from the NPC and Disassembly of the mRNP***	2.71E-12	11
3	N-Glycan biosynthesis	3.33E-08	9	Exon Junction Complex	3.06E-12	16
3	***Pyrimidine metabolism***	3.33E-08	12	Cleavage at the 3'-Splice Site and Exon Ligation	3.06E-12	16
3	***Protein processing in endoplasmic reticulu***	1.15E-06	13	mRNA Splicing - Major Pathway	4.18E-12	16
3	***Inositol phosphate metabolism***	2.35E-06	8	Spliceosomal Intermediate C Complex	2.46E-11	15
3	***Phosphatidylinositol signaling system***	2.36E-06	9	Spliceosomal Active C Complex	2.46E-11	15
3	***Wnt signaling pathway***	2.82E-06	12	Formation of an intermediate Spliceosomal C complex	3.32E-11	15
3	***mRNA surveillance pathway***	4.04E-06	9	snRNP Assembly	4.40E-11	11
3	Progesterone-mediated oocyte maturat	4.48E-06	9	***Docking of the TAP:EJC Complex with the NPC***	9.59E-11	11
3	***Purine metabolism***	6.79E-06	12	***Transport of Mature mRNA derived from an Intron-Containing Trans***	1.23E-10	11
3	p53 signaling pathway	7.25E-06	8	***Nuclear export of snRNA transcripts***	1.50E-10	9
3				***Docking of Mature Histone mRNA complex:TAP at the NPC***	2.18E-10	9
3				***Transport of the SLBP independent Mature mRNA***	3.12E-10	9
3				***Docking of Mature Replication Dependent Histone mRNA with the N***	3.12E-10	9
3				***Transport of the SLBP Dependant Mature mRNA***	4.40E-10	9
3				***Nuclear Pore Complex (NPC)***	1.62E-09	8
3				***nucleoporin-associated Rev:Importin-beta:B23 complex***	2.35E-09	8
3				***Addition of Nucleotides 5 through 9 on the growing Transcript***	2.67E-09	9
3				***HIV-1 transcription complex containing 4 nucleotide long transcript***	2.67E-09	9
3				***Addition of nucleotides 5 through 9 on the growing HIV-1 transcript***	2.67E-09	9
3				***pol II transcription complex containing 4 nucleotide long transcript***	2.67E-09	9
3				***Regulation of Glucokinase by Glucokinase Regulatory Protein***	3.36E-09	8
3				***Transport of the Mature Intronless Transcript Derived Histone mRNA***	3.36E-09	8
3				***Transport of the Mature intronless transcript derived mRNA:TAP:Aly***	3.36E-09	8
3				***GCK1:GKRP [cytosol] => GCK1:GKRP [nucleoplasm]***	3.36E-09	8
3				***RNA Polymerase II Transcription Initiation***	3.48E-09	9
3				***RNA Polymerase II Transcription Pre-Initiation And Promoter Openin***	3.48E-09	9
3				***RNA Polymerase II Promoter Opening: First Transition***	3.48E-09	9
3				Newly Formed Phosphodiester Bond Stabilized and PPi Releasec	3.48E-09	9
3				***RNA Polymerase II Promoter Escape***	3.48E-09	9
3				***Pol II Promoter Escape Complex***	3.48E-09	9
3				***HIV-1 Transcription Initiation***	3.48E-09	9
3				***Transcription of the HIV genome***	3.48E-09	9
3				snRNP nuclear import and release	3.48E-09	9
3				***Addition of the fourth nucleotide on the Nascent Transcript: Second***	3.48E-09	9
3				***HIV-1 transcription complex containing 3 nucleotide long transcript***	3.48E-09	9
3				***HIV-1 initiation complex with phosphodiester-PPi intermediate***	3.48E-09	9
3				***HIV-1 open pre-initiation complex***	3.48E-09	9
3				***HIV-1 Promoter Escape Complex***	3.48E-09	9
3				***RNA Polymerase II HIV-1 Promoter Escape***	3.48E-09	9
3				***NTP Binds Active Site of RNA Polymerase II***	3.48E-09	9
3				***Nucleophillic Attack by 3'-hydroxyl Oxygen of nascent transcript on t***	3.48E-09	9
3				***Addition of the third nucleotide on the nascent transcript***	3.48E-09	9
3				***HIV-1 transcription complex***	3.48E-09	9
3				***pol II transcription complex containing 3 Nucleotide long transcript***	3.48E-09	9
3				***Pol II Initiation complex with phosphodiester-PPi intermediate***	3.48E-09	9
3				***pol II closed pre-initiation complex***	3.48E-09	9
3				***Pol II initiation complex***	3.48E-09	9
3				***pol II open pre-initiation complex***	3.48E-09	9
3				***HIV-1 Promoter Opening: First Transition***	3.48E-09	9
3				***Addition of the third nucleotide on the nascent HIV-1 transcript***	3.48E-09	9
3				***Addition of the fourth nucleotide on the nascent HIV-1 transcript: Se***	3.48E-09	9
3				***Fall Back to Closed Pre-initiation Complex***	3.48E-09	9
3				***Nucleophillic attack by 3'-hydroxyl oxygen of nascent HIV-1 transcrip***	3.48E-09	9
3				Newly formed phosphodiester bond stabilized and PPi released	3.48E-09	9
3				***NTP binds active site of RNA Polymerase II in HIV-1 open pre-initiatio***	3.48E-09	9
3				***HIV-1 closed pre-initiation complex***	3.48E-09	9
3				***pol II transcription complex***	3.48E-09	9
3				***HIV-1 initiation complex***	3.48E-09	9
3				***Nuclear import of Rev protein***	4.71E-09	8
3				***Transport of the Mature IntronlessTranscript Derived Histone mRNA***	4.71E-09	8
3				***Release of the Mature intronless derived mRNA, TAP, and Aly/Ref fr***	4.71E-09	8
3				***Release of the SLBP independent Histone mRNA from the NPC***	4.71E-09	8

3				***Release of the Mature intronless transcript derived Histone mRNA:S***	6.50E-09	8
3				***RNA Polymerase II Pre-transcription Events***	7.99E-09	10
3				Microtubule-bound kinetochore	1.59E-08	11
3				Kinetochore	1.59E-08	11
3				Spliceosomal A Complex	3.22E-08	11
3				Formation of the Spliceosomal E complex	3.22E-08	10
3				Spliceosomal E Complex	3.22E-08	10
3				***COPII (Coat Protein 2) Mediated Vesicle Transport***	9.67E-08	5
3				Spliceosomal B Complex	1.01E-07	11
3				Formation of the Spliceosomal B Complex	1.01E-07	11
3				***pol II promoter:TFIID:TFIIA:TFIIB:Pol II:TFIIF:TFIIE complex***	2.13E-07	7
3				cNAP-1 depleted centrosome	2.61E-07	9
3				Nlp-depleted centrosome	2.61E-07	9
3				Plk1-mediated phosphorylation of Nlp	3.04E-07	9
3				Loss of C-Nap-1 from centrosomes	3.04E-07	9
3				Centrosomes containing recruited CDK11p58	3.04E-07	9
3				centrosome	3.04E-07	9
3				Centrosome associated Plk1	3.04E-07	9
3				Loss of Nlp from mitotic centrosomes	3.04E-07	9
3				Dissociation of Phospho-Nlp from the centrosome	3.04E-07	9
3				centrosome containing phosphorylated Nlp	3.04E-07	9
3				***Synthesis of PIPs at the Golgi membrane***	5.86E-07	5
3				Cdc20:Phospho-APC/C mediated degradation of Cyclin A	6.14E-07	9
3				Degradation multiubiquitinated Cyclin A	6.14E-07	9
3				***mRNA 3'-end processing***	6.88E-07	7
3				***Cleavage of mRNA at the 3'-end***	6.88E-07	7
3				***Ligated exon containing complex***	6.88E-07	7
3				***Signaling by SCF-KIT***	6.88E-07	7
3				Mature centrosomes enriched in gamma-TURC complexes	7.01E-07	9
3				***Antigen processing: Ubiquitination & Proteasome degradation***	9.81E-07	8
3				***HIV-1 promoter:TFIID:TFIIA:TFIIB complex***	1.44E-06	5
3				***Biosynthesis of the N-glycan precursor (dolichol lipid-linked oligosac***	1.44E-06	5
3				***Cleavage of Intronless Pre-mRNA at 3'-end***	1.44E-06	5
3				***pol II promoter:TFIID:TFIIA:TFIIB complex***	1.44E-06	5
3				***Cleavage and polyadenylation of Intronless Pre-mRNA***	1.44E-06	5
3				***intronless pre-mRNA cleavage complex***	1.44E-06	5
3				***Processing of Intronless Pre-mRNAs***	1.44E-06	5
3				***PPARG:Fatty Acid:RXRA:Mediator:Coactivator Complex***	1.84E-06	7
3				***Expression of CEBPA***	1.84E-06	7
3				***TRAP coactivator complex***	2.14E-06	5
3				***Expression of FABP4 (aP2)***	2.20E-06	7
3				***Expression of Phosphoenolpyruvate carboxykinase 1 (PEPCK-C)***	2.20E-06	7
3				***Expression of Lipoprotein lipase (LPL)***	2.20E-06	7
3				***Expression of Leptin***	2.20E-06	7
3				***Expression of Adiponectin***	2.20E-06	7
3				***Expression of ANGPTL4***	2.20E-06	7
3				***Expression of CD36 (platelet glycoprotein IV, FAT)***	2.20E-06	7
3				***Expression of Perilipin (PLIN)***	2.20E-06	7
3				Degradation of multiubiquitinated Cdh1	2.69E-06	8
3				Autodegradation of Cdh1 by Cdh1:APC/C	2.69E-06	8
3				***HIV-1 promoter:TFIID:TFIIA:TFIIB:Pol II:TFIIF complex****	2.74E-06	6
3				***pol II promoter:TFIID:TFIIA:TFIIB:Pol II:TFIIF complex***	2.74E-06	6
3				***PPARG:RXRA Heterodimer Binds to Fatty Acid-like Ligands***	3.66E-06	7
3				***Elongation of pre-rRNA transcript***	4.30E-06	5
3				***RNA Polymerase I promoter escape complex***	4.30E-06	5
3				APC/C:Cdh1-mediated degradation of Skp2	4.50E-06	8
3				Degradation of multiubiquitinated Securin	4.50E-06	8
3				APC/C:Cdc20 mediated degradation of Securin	4.50E-06	8
3				***RNA Polymerase I:rRNATranscript:TTF-1:Sal Box Complex***	5.87E-06	5
3				***RNA Polymerase I Transcription Initiation complex***	5.87E-06	5
3				Inactivation of APC/C via direct inhibition of the APC/C complex	5.87E-06	5
3				MCC:APC/C complex	5.87E-06	5
3				Activation of APC/C:Cdc20 by dissociation of Cdc20:phospho-AP	5.87E-06	5
3				***Loss of Rrn3 from RNA Polymerase I promoter escape complex***	5.87E-06	5
3				***Mediator Complex (consensus)***	6.48E-06	6
3				Degradation of multiubiquitinated cell cycle proteins	7.25E-06	8
3				***RNA Polymerase I Transcription Termination***	7.87E-06	5
3				***Dissociation of PTRF:Polymerase I/Nascent Pre rRNA Complex:TTF-I:***	7.87E-06	5
3				Nek2A:MCC:APC/C complex	7.87E-06	5
3				Cdc2:Cyclin A:MCC:APC/C complex	7.87E-06	5
3				***PTRF:Polymerase I/Nascent Pre rRNA Complex:TTF-I:Sal Box***	7.87E-06	5
3				***capped, methylated pre-mRNP:CBC complex***	7.88E-06	6
3				APC/C:Cdh1 mediated degradation of Cdc20 and other APC/C:C	8.13E-06	8
4	Olfactory transduction	1.45E-38	33	Olfactory Receptor - G Protein olfactory trimer complex formati	9.28E-42	34
4				OR - G Protein Trimer Complex	9.28E-42	34

5	No match			No match		

EXPERIMENT: E-GEOD-43582, Downregulated Canonical Pathways

Maximum stable clusters : 4

Functional enrichment p -value <10^{-5}

cluster	KEGG pathway	p-value	# of proteins	Reactome pathway	p-value	# of proteins
1	Neuroactive ligand-receptor interactior	1.56E-52	60	G alpha (i) signalling events	2.41E-34	38
1	MAPK signaling pathway	4.31E-28	40	The Ligand:GPCR:Gi complex dissociates	9.30E-32	34
1	Cytokine-cytokine receptor interaction	3.55E-27	39	G alpha (q) signalling events	4.25E-20	25
1	Calcium signaling pathway	8.83E-21	28	The Ligand:GPCR:Gq complex dissociates	4.42E-20	24
1	Chemokine signaling pathway	4.29E-20	28	***Activation of voltage gated Potassium channels***	5.95E-10	10
1	Glutamatergic synapse	9.92E-20	24	***Octamer of Voltage gated K+ channels***	5.95E-10	10
1	Cholinergic synapse	1.42E-11	16	Collagen biosynthesis and modifying enzymes	2.02E-08	10
1	Hematopoietic cell lineage	5.94E-11	14	Association of procollagen chains	1.20E-08	9
1	Jak-STAT signaling pathway	2.22E-10	17	The Ligand:GPCR:Gs complex dissociates	4.78E-07	9
1	***T cell receptor signaling pathway***	7.86E-10	14	G alpha (s) signalling events	1.09E-05	9
1	***Protein digestion and absorption***	1.29E-09	12	***Termination of O-glycan biosynthesis***	2.61E-08	7
1	Toll-like receptor signaling pathway	3.86E-09	13	***NR-MED1 Coactivator Complex***	1.29E-06	7
1	***Chagas disease (American trypanosomias***	4.38E-09	13	***Activation of GIRK/Kir3 Channels***	3.35E-08	6
1	Pathways in cancer	5.08E-09	22	***GABA B receptor G-protein beta-gamma and Kir3 channel complex***	3.35E-08	6
1	Arachidonic acid metabolism	2.02E-08	10	***GABA B receptor G-protein beta-gamma complex***	3.35E-08	6
1	***Salivary secretion***	3.79E-08	11	***ST6GALNAC3/4 can add a sialic acid to the sialyl T antigen to form tl***	8.70E-08	6
1	***Toxoplasmosis***	1.07E-07	13	***GalNAc alpha-2,6-sialyltransferase II can add a sialic acid to the T ar***	8.70E-08	6
1	Rheumatoid arthritis	1.17E-07	11	***The Ligand:GPCR:G12/13 complex dissociates***	4.85E-09	5
1	Vascular smooth muscle contraction	1.55E-07	12	***HDL-mediated lipid transport***	3.51E-07	5
1	***Fc epsilon RI signaling pathway***	2.14E-07	10	***G-protein beta-gamma subunits***	9.53E-07	5
1	***Osteoclast differentiation***	3.62E-07	12	***G-beta:G-gamma dimer***	1.46E-06	5
1	Amoebiasis	5.82E-07	11	***Glucagon:GCGR mediates GTP-GDP exchange***	2.17E-06	5
1	Gastric acid secretion	1.35E-06	9	***G-protein with G(s) alpha:GDP***	2.17E-06	5
1	Cardiac muscle contraction	1.93E-06	9	***C1GALT1 transfers galactose to the Tn antigen forming Core 1 glyco***	2.17E-06	5
1	***Pancreatic secretion***	1.99E-06	10	***Addition of galactose to Core 6 glycoprotein***	2.17E-06	5
1	***Linoleic acid metabolism***	5.38E-06	6	***Addition of GalNAc to the Tn antigen via an alpha-1,6 linkage forms***	2.17E-06	5
				Sialyltransferase I can add sialic acid to the T antigen at the alpha 6	2.17E-06	5
				ST3GAL1-4 can add a sialic acid to the T antigen at the alpha 3 positi	2.17E-06	5
				Addition of galactose to the Tn antigen via an alpha-1,3 linkage forr	2.17E-06	5
				Sialyltransferase I can add sialic acid to the Tn antigen at the alpha (	2.17E-06	5
				Addition of GlcNAc to Core 3 forms a Core 4 glycoprotein	2.17E-06	5
				Addition of GlcNAc to the Tn antigen via an alpha-1,3 linkage forms	2.17E-06	5
				Addition of GlcNAc to the Tn antigen via a beta-1,6 linkage forms a (	2.17E-06	5
				Addition of GalNAc to mucins to form the Tn antigen	2.17E-06	5
				Addition of GlcNAc to the Tn antigen forms a Core 3 glycoprotein	2.17E-06	5
				Addition of GlcNAc to the T antigen forms a Core 2 glycoprotein	2.17E-06	5
2	Neuroactive ligand-receptor interactior	1.26E-27	33	G alpha (i) signalling events	1.84E-12	16
2	MAPK signaling pathway	2.40E-25	31	G alpha (s) signalling events	1.13E-13	14
2	Cytokine-cytokine receptor interaction	4.58E-23	29	G alpha (q) signalling events	2.83E-11	14
2	Calcium signaling pathway	4.41E-13	17	The Ligand:GPCR:Gi complex dissociates	2.83E-11	14
2	Chemokine signaling pathway	1.33E-11	16	The Ligand:GPCR:Gs complex dissociates	1.60E-14	13
2	Cardiac muscle contraction	7.63E-11	11	The Ligand:GPCR:Gq complex dissociates	9.39E-11	13
2	Jak-STAT signaling pathway	1.54E-10	14			
2	Dilated cardiomyopathy	6.68E-10	11			
2	Glutamatergic synapse	1.87E-09	12			
2	***Regulation of actin cytoskeleton***	7.14E-09	14			
2	Hematopoietic cell lineage	7.48E-09	10			
2	Pathways in cancer	4.73E-08	16			
2	Hypertrophic cardiomyopathy (HCM)	7.18E-08	9			
2	GnRH signaling pathway	2.79E-07	9			
2	***Alzheimer's disease***	2.66E-06	10			
2	Natural killer cell mediated cytotoxicity	2.92E-06	9			
2	***Measles***	3.32E-06	9			
2	***Melanoma***	3.33E-06	7			
2	***Long-term depression***	3.33E-06	7			
2	***Autoimmune thyroid disease***	6.36E-06	6			
3	Cytokine-cytokine receptor interaction	9.53E-21	25	G alpha (i) signalling events	3.73E-15	17
3	Calcium signaling pathway	3.96E-17	19	Collagen biosynthesis and modifying enzymes	1.76E-11	10
3	Neuroactive ligand-receptor interactior	8.66E-16	21	The Ligand:GPCR:Gi complex dissociates	2.56E-11	13
3	Cholinergic synapse	4.37E-15	15	The Ligand:GPCR:Gq complex dissociates	1.08E-10	12
3	MAPK signaling pathway	1.03E-13	19	G alpha (q) signalling events	3.90E-10	12
3	Chemokine signaling pathway	4.90E-13	16	***CCBP2:CCBP2 ligands***	2.60E-09	5
3	GnRH signaling pathway	7.66E-12	12	Association of procollagen chains	2.36E-08	7
3	***Fructose and mannose metabolism***	1.53E-10	8			
3	Hypertrophic cardiomyopathy (HCM)	5.85E-10	10			
3	Vascular smooth muscle contraction	7.35E-10	11			
3	***Focal adhesion***	2.67E-09	13			
3	Amoebiasis	5.49E-09	10			

3	Dilated cardiomyopathy	2.30E-08	9			
3	***Arrhythmogenic right ventricular cardiom***	6.89E-08	8			
3	Rheumatoid arthritis	3.27E-07	8			
3	***Long-term potentiation***	6.50E-07	7			
3	Gastric acid secretion	9.69E-07	7			
3	Jak-STAT signaling pathway	2.18E-06	9			
3	***ECM-receptor interaction***	3.30E-06	7			
3	***Neurotrophin signaling pathway***	3.63E-06	8			
3	***African trypanosomiasis***	4.23E-06	5			
3	***Influenza A***	4.49E-06	9			
3	Retinol metabolism	4.94E-06	6			
3	Natural killer cell mediated cytotoxicity	5.19E-06	8			
3	***Glioma***	6.02E-06	6			
3	***Melanogenesis***	7.97E-06	7			
3	Toll-like receptor signaling pathway	9.12E-06	7			
4	Neuroactive ligand-receptor interactior	9.68E-15	17	The Ligand:GPCR:Gi complex dissociates	4.51E-11	11
4	Cytokine-cytokine receptor interaction	1.68E-12	15	G alpha (i) signalling events	2.83E-10	11
4	Retinol metabolism	3.61E-10	8	The Ligand:GPCR:Gq complex dissociates	1.81E-06	7
4	***Drug metabolism - cytochrome P450***	1.47E-09	8	G alpha (q) signalling events	3.77E-06	7
4	***Metabolism of xenobiotics by cytochrome***	3.42E-08	7			
4	Calcium signaling pathway	1.35E-07	9			
4	Arachidonic acid metabolism	3.67E-07	6			
4	Chemokine signaling pathway	2.57E-06	8			
4	***Glycolysis / Gluconeogenesis***	9.77E-06	5			

EXPERIMENT: E-GEOD-57418, Upregulated Canonical Pathways

Maximum stable clusters : 9

Functional enrichment p -value <10^{-5}

cluster	KEGG pathway	p-value	# of proteins	Reactome pathway	p-value	# of proteins
1	MAPK signaling pathway	2.67E-06	8	Destabilization of mRNA by AUF1 (hnRNP D0)	6.74E-07	5
1				Activation of NF-kappaB in B Cells	9.96E-07	5
2	Spliceosome	1.17E-24	25	mRNA Splicing - Major Pathway	2.05E-22	22
2	***Primary immunodeficiency***	1.55E-15	12	Formation of an intermediate Spliceosomal C complex	2.79E-21	21
2	T cell receptor signaling pathway	6.04E-13	15	Exon Junction Complex	3.48E-21	21
2	Apoptosis	2.31E-09	11	Cleavage at the 3'-Splice Site and Exon Ligation	3.48E-21	21
2	B cell receptor signaling pathway	6.20E-09	10	Spliceosomal B Complex	4.62E-21	20
2	Pathways in cancer	1.70E-08	18	Formation of the Spliceosomal B Complex	4.62E-21	20
2	***RNA polymerase***	6.29E-07	6	Spliceosomal Intermediate C Complex	4.66E-20	20
2	Chronic myeloid leukemia	8.77E-07	8	Spliceosomal Active C Complex	4.66E-20	20
2	***Osteoclast differentiation***	9.14E-07	10	Spliceosomal active C complex with lariat containing, 5'-end clea	5.79E-20	20
2	VEGF signaling pathway	1.51E-06	8	Lariat Formation and 5'-Splice Site Cleavage	5.79E-20	20
2	Natural killer cell mediated cytotoxicity	1.53E-06	10	Spliceosomal A Complex	1.20E-17	17
2	Tuberculosis	3.49E-06	11	***Formation of the Spliceosomal E complex***	1.25E-13	13
2				***Spliceosomal E Complex***	1.25E-13	13
2				mRNA Splicing - Minor Pathway	2.82E-13	11
2				ATAC B Complex	2.82E-13	11
2				ATAC C Complex with lariat containing 5'-end cleaved mRNA	2.82E-13	11
2				ATAC C Complex	2.82E-13	11
2				Formation of AT-AC C complex	2.82E-13	11
2				ATAC spliceosome mediated 3' splice site cleavage, exon ligatior	2.82E-13	11
2				***RNA Polymerase II CTD (phosphorylated) binds to CE***	5.23E-11	8
2				***RNA Pol II with phosphorylated CTD: CE complex with activatec***	5.23E-11	8
2				***RNA Pol II with phosphorylated CTD: CE complex***	5.23E-11	8
2				***Transfer of GMP from the capping enzyme GT site to 5'-end of***	7.95E-11	8
2				***Methylation of GMP-cap by RNA Methyltransferase***	7.95E-11	8
2				***SPT5 subunit of Pol II binds the RNA triphosphatase (RTP)***	7.95E-11	8
2				***Capping complex (initial)***	7.95E-11	8
2				***Hydrolysis of the 5'-end of the nascent transcript by the cappin***	7.95E-11	8
2				***RNA Pol II CTD phosphorylation and interaction with CE***	7.95E-11	8
2				***Formation of the CE:GMP intermediate complex***	7.95E-11	8
2				***CE:Pol II CTD:Spt5 complex***	7.95E-11	8
2				***Capping complex (intermediate)***	7.95E-11	8
2				***Capping complex (hydrolyzed)***	7.95E-11	8
2				***Covalent CE:GMP intermediate complex***	7.95E-11	8
2				***Capping complex (with freed 5'- GMP)***	7.95E-11	8
2				***Capping complex (GpppN..)***	7.95E-11	8
2				***mRNA Capping***	1.72E-10	8
2				***Internal Methylation of mRNA***	1.84E-10	7
2				intron-containing complex	1.89E-10	9
2				***post exon ligation complex***	7.89E-10	7
2				***ATAC A Complex***	1.19E-09	8
2				***capped, methylated pre-mRNP:CBC complex***	1.57E-09	8
2				***Addition of nucleotides between position +11 and +30***	1.79E-09	7
2				***Pol II transcription complex with (ser5) phosphorylated CTD co***	1.79E-09	7

2	***Pol II transcription complex containing extruded transcript to +***	1.79E-09	7
2	***pol II transcription complex containing 11 nucleotide long trans***	1.79E-09	7
2	***pol II transcription complex containing 9 nucleotide long transc***	1.79E-09	7
2	***Phosphorylation (Ser5) of RNA pol II CTD***	1.79E-09	7
2	***Addition of nucleotides 10 and 11 on the growing transcript: Th***	1.79E-09	7
2	***Addition of nucleotides between position +11 and +30 on HIV-1***	1.79E-09	7
2	***RNA Polymearse II:NTP:TFIIF complex***	1.79E-09	7
2	***Pol II transcription complex containing transcript to +30***	1.79E-09	7
2	***HIV-1 transcription complex containing 9 nucleotide long trans***	1.79E-09	7
2	***HIV-1 transcription complex containing 11 nucleotide long tran***	1.79E-09	7
2	***Addition of nucleotides 10 and 11 on the growing HIV-1 transcr***	1.79E-09	7
2	***HIV-1 transcription complex containing extruded transcript to***	1.79E-09	7
2	***HIV-1 transcription complex containing transcript to +30***	1.79E-09	7
2	***HIV-1 transcription complex with (ser5) phosphorylated CTD co***	1.79E-09	7
2	***RNA Polymerase II (unphosphorylated):TFIIF complex***	2.63E-09	6
2	***RNA Polymerase II holoenzyme complex (hyperphosphorylated***	2.63E-09	6
2	***RNA Polymerase II holoenzyme complex (hypophosphorylated)***	2.63E-09	6
2	***HIV-1 Polymerase II (phosphorylated):TFIIF:capped pre-mRNA***	2.63E-09	6
2	***RNA polymerase II (phosphorylated):TFIIF complex***	2.63E-09	6
2	***RNA Polymerase II (phosphorylated):TFIIF:capped pre-mRNA***	2.63E-09	6
2	***pol II transcription complex containing 4-9 nucleotide long tran***	3.70E-09	7
2	***HIV-1 transcription complex containing 4-9 nucleotide long tra***	3.70E-09	7
2	***RNA Polymerase II Transcription Elongation***	4.34E-09	8
2	***Recruitment of elongation factors to form HIV-1 elongation con***	4.34E-09	8
2	***Addition of nucleotides leads to transcript elongation***	4.34E-09	8
2	***Elongation complex***	4.34E-09	8
2	***HIV-1 elongation complex***	4.34E-09	8
2	***Hypophosphorylation of RNA Pol II CTD by FCP1P protein***	5.17E-09	7
2	***Formation of RNA Pol II elongation complex***	6.88E-09	8
2	***Formation of HIV-1 elongation complex in the absence of HIV-1***	6.88E-09	8
2	***Antigen Activates B Cell Receptor Leading to Generation of Sec***	7.12E-09	7
2	***capped, methylated pre-mRNA:CBC Complex***	7.54E-09	6
2	***HIV-1 capped pre-mRNA:CBC:RNA Pol II (phosphorylated) comp***	7.54E-09	6
2	***capped pre-mRNA:CBC:RNA Pol II (phosphorylated) complex***	7.54E-09	6
2	***RNA Pol II (hypophosphorylated):capped pre-mRNA complex***	1.20E-08	6
2	***HIV-1 paused processive elongation complex***	1.70E-08	7
2	***2-4 nt.backtracking of Pol II complex on the template leading t***	1.70E-08	7
2	***Resumption of elongation after recovery from pausing***	1.70E-08	7
2	***Formation of the Early Elongation Complex***	1.70E-08	7
2	***Abortive termination of HIV-1 elongation after arrest***	1.70E-08	7
2	***Elongation arrest and recovery***	1.70E-08	7
2	***HIV-1 elongation arrest and recovery***	1.70E-08	7
2	***Pausing and recovery of HIV-1 elongation***	1.70E-08	7
2	***Formation of the HIV-1 Early Elongation Complex***	1.70E-08	7
2	***Processive elongation complex***	1.70E-08	7
2	***Arrested processive elongation complex***	1.70E-08	7
2	***Paused processive elongation complex***	1.70E-08	7
2	***HIV-1 processive elongation complex***	1.70E-08	7
2	***Resumption of elongation of HIV-1 transcript after recovery fro***	1.70E-08	7
2	***Aborted elongation complex after arrest***	1.70E-08	7
2	***Elongation complex prior to separation***	1.70E-08	7
2	***Elongation complex with separated and uncleaved transcript***	1.70E-08	7
2	***2-4 nt.backtracking of Pol II complex on the HIV-1 template lea***	1.70E-08	7
2	***HIV-1 arrested processive elongation complex***	1.70E-08	7
2	***HIV-1 aborted elongation complex after arrest***	1.70E-08	7
2	***Abortive termination of elongation after arrest***	1.70E-08	7
2	***Elongating transcript encounters a lesion in the template***	1.70E-08	7
2	***RNA Pol II (hypophosphorylated) complex bound to DSIF protei***	1.83E-08	6
2	***Docking of the TAP:EJC Complex with the NPC***	4.06E-08	8
2	***Transport of Mature mRNA derived from an Intron-Containing***	4.83E-08	8
2	***DSIF:NELF:early elongation complex***	5.58E-08	6
2	***Abortive elongation of HIV-1 transcript in the absence of Tat***	5.58E-08	6
2	***DSIF:NELF:early elongation complex after limited nucleotide ac***	5.58E-08	6
2	***Early elongation complex with separated aborted transcript***	5.58E-08	6
2	***Aborted early elongation complex***	5.58E-08	6
2	***Formation of DSIF:NELF:HIV-1 early elongation complex***	5.58E-08	6
2	***Aborted HIV-1 early elongation complex***	5.58E-08	6
2	***RNA Polymerase II holoenzyme complex (unphosphorylated)***	6.67E-08	5
2	***RNA Polymerase II holoenzyme complex (generic)***	6.67E-08	5
2	***RNA Polymerase II holoenzyme complex (phosphorylated)***	6.67E-08	5
2	***Active Pol II complex with repaired DNA template:mRNA hybri***	6.67E-08	5
2	***Stalled Pol II in TC-NER***	6.67E-08	5
2	***Stalled Pol II complex with damaged DNA hybrid***	6.67E-08	5
2	***Active Pol II transcription complex with damaged DNA hybrid***	6.67E-08	5
2	***RNA Polymerase II holoenzyme complex (hyperphosphorylated***	6.67E-08	5
2	***Early elongation complex with hyperphosphorylated Pol II CTD***	7.74E-08	6
2	***HIV-1 early elongation complex with hyperphosphorylated Pol***	7.74E-08	6

2				***Addition of Nucleotides 5 through 9 on the growing Transcript***	1.12E-07	7
2				***HIV-1 transcription complex containing 4 nucleotide long trans***	1.12E-07	7
2				***Addition of nucleotides 5 through 9 on the growing HIV-1 trans***	1.12E-07	7
2				***pol II transcription complex containing 4 nucleotide long transc***	1.12E-07	7
2				***RNA Polymerase II Pre-transcription Events***	1.26E-07	8
2				***RNA Polymerase II Transcription Initiation***	1.36E-07	7
2				***RNA Polymerase II Transcription Pre-Initiation And Promoter O***	1.36E-07	7
2				***RNA Polymerase II Promoter Opening: First Transition***	1.36E-07	7
2				Newly Formed Phosphodiester Bond Stabilized and PPi Releasec	1.36E-07	7
2				***RNA Polymerase II Promoter Escape***	1.36E-07	7
2				***Pol II Promoter Escape Complex***	1.36E-07	7
2				***HIV-1 Transcription Initiation***	1.36E-07	7
2				***Transcription of the HIV genome***	1.36E-07	7
2				***Addition of the fourth nucleotide on the Nascent Transcript: Se***	1.36E-07	7
2				***HIV-1 transcription complex containing 3 nucleotide long trans***	1.36E-07	7
2				***HIV-1 initiation complex with phosphodiester-PPi intermediate***	1.36E-07	7
2				***HIV-1 open pre-initiation complex***	1.36E-07	7
2				***HIV-1 Promoter Escape Complex***	1.36E-07	7
2				***RNA Polymerase II HIV-1 Promoter Escape***	1.36E-07	7
2				***NTP Binds Active Site of RNA Polymerase II***	1.36E-07	7
2				***Nucleophillic Attack by 3'-hydroxyl Oxygen of nascent transcrip***	1.36E-07	7
2				***Addition of the third nucleotide on the nascent transcript***	1.36E-07	7
2				***HIV-1 transcription complex***	1.36E-07	7
2				***pol II transcription complex containing 3 Nucleotide long trans***	1.36E-07	7
2				***Pol II Initiation complex with phosphodiester-PPi intermediate***	1.36E-07	7
2				***pol II closed pre-initiation complex***	1.36E-07	7
2				***Pol II initiation complex***	1.36E-07	7
2				***pol II open pre-initiation complex***	1.36E-07	7
2				***HIV-1 Promoter Opening: First Transition***	1.36E-07	7
2				***Addition of the third nucleotide on the nascent HIV-1 transcrip***	1.36E-07	7
2				***Addition of the fourth nucleotide on the nascent HIV-1 transcri***	1.36E-07	7
2				***Fall Back to Closed Pre-initiation Complex***	1.36E-07	7
2				***Nucleophillic attack by 3'-hydroxyl oxygen of nascent HIV-1 tra***	1.36E-07	7
2				Newly formed phosphodiester bond stabilized and PPi released	1.36E-07	7
2				***NTP binds active site of RNA Polymerase II in HIV-1 open pre-in***	1.36E-07	7
2				***HIV-1 closed pre-initiation complex***	1.36E-07	7
2				***pol II transcription complex***	1.36E-07	7
2				***HIV-1 initiation complex***	1.36E-07	7
2				***Activation of Gene Expression by SREBP (SREBF)***	1.36E-07	7
2				***Hyperphosphorylation (Ser2) of RNA Pol II CTD by P-TEFb comp***	1.41E-07	6
2				***Formation of transcription-coupled NER (TC-NER) repair compl***	3.14E-07	6
2				***Dual incision reaction in TC-NER***	3.14E-07	6
2				***Assembly of repair proteins at the site of Pol II blockage***	3.14E-07	6
2				***Displacement of stalled Pol II from the lesion site***	3.14E-07	6
2				***HIV-1 promoter:TFIID:TFIIA:TFIIB:Pol II:TFIIF complex****	4.00E-07	6
2				***pol II promoter:TFIID:TFIIA:TFIIB:Pol II:TFIIF complex***	4.00E-07	6
2				***pol II promoter:TFIID:TFIIA:TFIIB:Pol II:TFIIF:TFIIE complex***	6.29E-07	6
2				***Downregulation of SMAD2/3:SMAD4 transcriptional activity***	1.16E-06	5
2				***Cholesterol biosynthesis***	1.16E-06	5
2				***Transport of the export-competent complex through the NPC***	1.70E-06	6
2				***Release from the NPC and Disassembly of the mRNP***	2.04E-06	6
2				***Downstream TCR signaling***	2.86E-06	6
2				***MicroRNA (miRNA) Biogenesis***	3.44E-06	5
2				***PPARG:Fatty Acid:RXRA:Mediator:Coactivator Complex***	3.94E-06	6
2				***Expression of CEBPA***	3.94E-06	6
2				***Expression of FABP4 (aP2)***	4.60E-06	6
2				***Expression of Phosphoenolpyruvate carboxykinase 1 (PEPCK-C)***	4.60E-06	6
2				***Expression of Lipoprotein lipase (LPL)***	4.60E-06	6
2				***Expression of Leptin***	4.60E-06	6
2				***Expression of Adiponectin***	4.60E-06	6
2				***Expression of ANGPTL4***	4.60E-06	6
2				***Expression of CD36 (platelet glycoprotein IV, FAT)***	4.60E-06	6
2				***Expression of Perilipin (PLIN)***	4.60E-06	6
2				***PPARG:RXRA Heterodimer Binds to Fatty Acid-like Ligands***	7.10E-06	6
2				***Transport of the Mature Intronless Transcript Derived Histone***	8.30E-06	5
2				***Transport of the Mature intronless transcript derived mRNA:TA***	8.30E-06	5
3	Pathways in cancer	2.14E-13	19	Exon Junction Complex	9.74E-10	10
3	Chemokine signaling pathway	1.39E-16	18	Cleavage at the 3'-Splice Site and Exon Ligation	9.74E-10	10
3	Insulin signaling pathway	1.92E-16	16	mRNA Splicing - Major Pathway	1.18E-09	10
3	Neurotrophin signaling pathway	1.99E-15	15	intron-containing complex	3.31E-09	7
3	T cell receptor signaling pathway	1.45E-13	13	Spliceosomal Intermediate C Complex	1.35E-08	9
3	Focal adhesion	4.28E-10	13	Spliceosomal Active C Complex	1.35E-08	9
3	***ErbB signaling pathway***	1.92E-13	12	Spliceosomal active C complex with lariat containing, 5'-end clea	1.47E-08	9
3	Natural killer cell mediated cytotoxicity	6.37E-10	11	Lariat Formation and 5'-Splice Site Cleavage	1.47E-08	9
3	MAPK signaling pathway	1.06E-06	11	Formation of an intermediate Spliceosomal C complex	1.61E-08	9
3	***Glioma***	5.68E-12	10	Spliceosomal B Complex	9.59E-08	8

3	***Renal cell carcinoma***	1.76E-11	10	Formation of the Spliceosomal B Complex	9.59E-08	8
3	Chronic myeloid leukemia	2.05E-11	10	mRNA Splicing - Minor Pathway	4.82E-06	5
3	Prostate cancer	2.19E-10	10	ATAC B Complex	4.82E-06	5
3	***Chagas disease (American trypanosom***	8.83E-10	10	ATAC C Complex with lariat containing 5'-end cleaved mRNA	4.82E-06	5
3	***Progesterone-mediated oocyte maturc***	2.74E-09	9	ATAC C Complex	4.82E-06	5
3	Influenza A	1.32E-06	9	Formation of AT-AC C complex	4.82E-06	5
3	***Endometrial cancer***	1.16E-09	8	ATAC spliceosome mediated 3' splice site cleavage, exon ligatior	4.82E-06	5
	Non-small cell lung cancer	1.36E-09	8			
	Acute myeloid leukemia	2.17E-09	8			
	Adipocytokine signaling pathway	9.64E-09	8			
	Melanoma	1.38E-08	8			
	B cell receptor signaling pathway	1.95E-08	8			
	VEGF signaling pathway	2.42E-08	8			
	Fc gamma R-mediated phagocytosis	1.14E-07	8			
	Oocyte meiosis	4.39E-07	8			
	Spliceosome	1.35E-06	8			
	Prion diseases	2.18E-09	7			
	mTOR signaling pathway	3.45E-08	7			
	Colorectal cancer	7.63E-08	7			
	Pancreatic cancer	1.73E-07	7			
	Long-term potentiation	2.38E-07	7			
	Fc epsilon RI signaling pathway	5.21E-07	7			
	Gap junction	1.23E-06	7			
	Apoptosis	1.23E-06	7			
	Toll-like receptor signaling pathway	3.44E-06	7			
	Cholinergic synapse	7.36E-06	7			
	Vascular smooth muscle contraction	7.81E-06	7			
	Bladder cancer	1.97E-07	6			
	Type II diabetes mellitus	4.08E-07	6			
4	Cytokine-cytokine receptor interaction	1.55E-21	22	None		
	Systemic lupus erythematosus	2.50E-17	14			
	Chemokine signaling pathway	1.93E-13	14			
	Pathways in cancer	3.97E-09	13			
	Focal adhesion	3.34E-08	10			
	Malaria	1.04E-07	6			
	ECM-receptor interaction	1.63E-07	7			
	Rheumatoid arthritis	2.24E-07	7			
	Amoebiasis	6.52E-07	7			
	Pertussis	9.38E-07	6			
	Tuberculosis	1.76E-06	8			
	Toxoplasmosis	2.93E-06	7			
	Apoptosis	3.22E-06	6			
	MAPK signaling pathway	4.66E-06	9			
5	Pathways in cancer	7.35E-08	9	***26S proteasome***	3.90E-08	5
5	Measles	9.61E-10	8	***Destruction of AUF1 and mRNA***	5.72E-08	5
5	Influenza A	6.99E-09	8	***Proteasomal clevage of exogenous antigen***	5.72E-08	5
5	Toll-like receptor signaling pathway	4.36E-09	7	***Cross-presentation of soluble exogenous antigens (endosomes,***	8.18E-08	5
5	RIG-I-like receptor signaling pathway	1.71E-08	6	***26S proteosome degrades ODC holoenzyme complex***	9.16E-08	5
5	Hepatitis C	7.23E-07	6	***Proteasomal cleavage of substrate***	9.16E-08	5
5	Toxoplasmosis	7.57E-07	6	***Proteasome mediated degradation of Cyclin D1***	1.02E-07	5
5	***Proteasome***	6.46E-08	5	***Ubiquitinated geminin is degraded by the proteasome***	1.02E-07	5
5	***NOD-like receptor signaling pathway***	2.50E-07	5	***Ubiquitinated Cdc6 is degraded by the proteasome***	1.02E-07	5
5	***Cytosolic DNA-sensing pathway***	3.26E-07	5	***Proteolytic degradation of ubiquitinated-Cdc25A***	1.02E-07	5
5				***Ubiquitinated Orc1 is degraded by the proteasome***	1.02E-07	5
5				***Proteasome mediated degradation of PAK-2p34***	1.02E-07	5
5				***Regulation of activated PAK-2p34 by proteasome mediated de***	1.02E-07	5
5				***Proteasome mediated degradation of COP1***	1.02E-07	5
5				***Ubiquitin-dependent degradation of Cyclin D1***	1.14E-07	5
5				***CDK-mediated phosphorylation and removal of Cdc6***	1.14E-07	5
5				***Autodegradation of the E3 ubiquitin ligase COP1***	1.27E-07	5
5				***Ubiquitin Mediated Degradation of Phosphorylated Cdc25A***	1.27E-07	5
5				Destabilization of mRNA by AUF1 (hnRNP D0)	1.40E-07	5
5				***Antigen processing: Ubiquitination & Proteasome degradation***	1.55E-07	5
5				***SCF-mediated degradation of Emi1***	1.55E-07	5
5				***SCF-beta-TrCP mediated degradation of Emi1***	1.55E-07	5
5				***Degradation of beta-catenin by the destruction complex***	1.72E-07	5
5				***Degradation of ubiquitinated -beta catenin by the proteasome***	1.72E-07	5
5				Activation of NF-kappaB in B Cells	2.08E-07	5
5				***Interferon gamma signaling***	2.29E-07	5
5				***CDT1 association with the CDC6:ORC:origin complex***	2.29E-07	5
5				***Degradation of multiubiquitinated Cdh1***	2.99E-07	5
5				***Autodegradation of Cdh1 by Cdh1:APC/C***	2.99E-07	5
5				***APC/C:Cdh1-mediated degradation of Skp2***	4.20E-07	5
5				***Degradation of multiubiquitinated Securin***	4.20E-07	5
5				***APC/C:Cdc20 mediated degradation of Securin***	4.20E-07	5

5				***Degradation multiubiquitinated Cyclin A***	5.33E-07	5
5				***Cdc20:Phospho-APC/C mediated degradation of Cyclin A***	5.33E-07	5
5				***Degradation of multiubiquitinated cell cycle proteins***	5.75E-07	5
5				***ER-Phagosome pathway***	5.75E-07	5
5				***APC/C:Cdh1 mediated degradation of Cdc20 and other APC/C:(***	6.20E-07	5
5				***SCF(Skp2)-mediated degradation of p27/p21***	2.06E-09	6
5				***Degradation of ubiquitinated p27/p21 by the 26S proteasome***	2.06E-09	6
5				***Orc1 removal from chromatin***	1.18E-08	6
6	Pathways in cancer	3.49E-11	13	Spliceosomal Intermediate C Complex	8.48E-07	6
6	Jak-STAT signaling pathway	9.53E-14	12	Spliceosomal Active C Complex	8.48E-07	6
6	Measles	1.52E-11	10	Spliceosomal active C complex with lariat containing, 5'-end clea	9.00E-07	6
6	Cytokine-cytokine receptor interaction	1.33E-08	10	Lariat Formation and 5'-Splice Site Cleavage	9.00E-07	6
6	***Cell cycle***	1.37E-07	7	Formation of an intermediate Spliceosomal C complex	9.55E-07	6
6	Neurotrophin signaling pathway	1.53E-07	7	Exon Junction Complex	1.01E-06	6
6	Spliceosome	1.71E-07	7	Cleavage at the 3'-Splice Site and Exon Ligation	1.01E-06	6
6	Hepatitis C	2.25E-07	7	mRNA Splicing - Major Pathway	1.14E-06	6
6	Chemokine signaling pathway	2.29E-06	7	Spliceosomal A Complex	6.23E-06	5
	Regulation of actin cytoskeleton	5.02E-06	7			
	Prostate cancer	4.19E-07	6			
	Influenza A	1.85E-05	6			
	Focal adhesion	4.56E-05	6			
	Pancreatic cancer	2.19E-06	5			
	Small cell lung cancer	8.92E-06	5			
7				None		
8				None		
9				None		

EXPERIMENT: E-GEOD-57418, Downregulated Canonical Pathways

Maximum stable clusters : 9

Functional enrichment p -value <10^{-5}

cluster	KEGG pathway	p-value	# of proteins	Reactome pathway	p-value	# of proteins
1	none			none		
2	Parkinson's disease	2.43E-67	46	Respiratory electron transport	3.15E-37	24
2	Huntington's disease	1.78E-64	49	***Complex I - NADH:Ubiquinone oxidoreductase***	4.13E-26	16
2	Alzheimer's disease	2.21E-61	46	***NADH enters the respiratory chain at Complex I***	4.13E-26	16
2	Oxidative phosphorylation	5.13E-59	42	***HP subcomplex***	1.25E-15	10
2	Metabolic pathways	5.76E-50	72	***ATPase-ATP complex***	8.76E-11	6
2	***Propanoate metabolism***	1.31E-14	10	***ATPase-ADP and Pi complex***	8.76E-11	6
2	Valine, leucine and isoleucine degradati	1.45E-14	11	***Enzyme-bound ATP is released***	8.76E-11	6
2	Cardiac muscle contraction	6.54E-12	11	***ATP is synthesized from ADP and Pi by ATPase***	8.76E-11	6
2	***Cholinergic synapse***	3.36E-11	12	***Formation of ATP by chemiosmotic coupling***	8.76E-11	6
2	***Calcium signaling pathway***	4.62E-10	13	***ADP and Pi bind to ATPase***	8.76E-11	6
2	***Citrate cycle (TCA cycle)***	1.04E-09	7	***ATPase complex***	8.76E-11	6
2	Melanogenesis	2.46E-09	10	***IP sub-complex***	7.55E-11	5
2	**Gastric acid secretion**	2.46E-09	9	***ATPase CF(0)***	4.49E-10	5
2	Lysosome	1.97E-08	10			
2	***Glutamatergic synapse***	2.91E-08	10			
2	***Pancreatic secretion***	3.35E-08	9			
2	***GnRH signaling pathway***	5.82E-07	8			
2	***Endocrine and other factor-regulated calci***	1.04E-06	6			
2	***beta-Alanine metabolism***	1.41E-06	5			
2	***Vascular smooth muscle contraction***	1.74E-06	8			
2	***Amyotrophic lateral sclerosis (ALS)***	1.93E-06	6			
2	***Salivary secretion***	2.02E-06	7			
2	***Butanoate metabolism***	2.44E-06	5			
3	Lysosome	3.34E-16	13	Respiratory electron transport	8.92E-09	7
3	Oxidative phosphorylation	9.70E-13	11	***Transferrin endocytosis and recycling***	3.09E-08	5
3	Alzheimer's disease	1.29E-11	11	***Rho GTPase cycle***	2.75E-07	6
3	Huntington's disease	8.40E-10	10			
3	Cardiac muscle contraction	2.54E-07	6			
3	Parkinson's disease	3.28E-07	7			
3	***Tuberculosis***	2.77E-06	7			
4	Lysosome	7.81E-11	10	None		
4	Valine, leucine and isoleucine degradati	2.79E-08	6			
4	***Hematopoietic cell lineage***	7.83E-08	7			
4	Melanogenesis	3.27E-06	6			
5	Metabolic pathways	4.63E-06	10	**MHC class II antigen presentation**	2.90E-08	5
5	Lysosome	1.13E-06	5			

5	***Phagosome***	2.68E-06	5	
6	***Pertussis***	7.51E-09	5	None
6	***Complement and coagulation cascades***	7.51E-09	5	
6	***Chagas disease (American trypanosomiasi***	4.93E-08	5	
7	none			None
8	Lysosome	2.40E-08	6	None
9	None			None
